# Supplementary material for: Sex-specific differences in children attending the emergency department: prospective observational study
Source: BMJ Open. 2020 Sep 17;10(9):e035918. doi: 10.1136/bmjopen-2019-035918 (PMC7500294; doi:10.1136/bmjopen-2019-035918)

Zachariasse et al. 2020

APPENDIX 1. PubMed literature search

We conducted a systematic literature search to identify articles describing any sex or gender differences in children visiting the emergency department (ED), including but not restricted to differences in presenting problem, disease presentation, disease severity, diagnostics, management and health care provider attitudes and behaviours.

We searched the Pubmed database for articles published from the year 2000 onwards, using search terms related to 1) age or gender (*gender [ti] or sex [ti]*); 2) emergency care (*emergency medicine [mh] or emergency service, hospital [mh] or emergency medical services [mh] or emergency department [tiab] or emergency room [tiab] or emergency medicine [tiab] or emergency service [tiab] or emergency services [tiab]*); and 3) paediatrics (*child [mh] OR infant [mh] OR adolescent [mh] OR Infant, Newborn [mh] OR child, preschool[mh] OR child* [ti] OR adolescent [ti] OR infant [ti] OR pediatr**)

This search identified a total of 134 papers of which 22 were included in the final selection. Papers were excluded because they did not (separately) address children (n=77), were not related to emergency medicine (n=27), were conducted in developing countries (n=3), or did not assess gender differences in patients (n=5).

Selected papers were divided into categories based on the research topic. The topics most commonly studied were identified: trauma and injuries including (sexual) assault (n=8)[1-8]; mental health issues, including intoxications (n=6)[9-14] and asthma (n=5)[15-19]. Other topics were general resource use (n=2)[20 21] and urinary tract infections (n=1)[22].

Table 1.1 Mental health (n=6)

Author, year	Setting	Period	n	Inclusion	Conclusion
Foto-Ozdemir, 2016	Emergency service at a Turkish Children's Hospital	-	64	Adolescents with a suicide attempt	There were no statistically significant differences between the girls and the boys with respect to the clinical characteristics of the suicide attempt and the rate of psychiatric disorders.
Kupferschmid, 2013	ED of a large university hospital in Germany	2004-2010	257	Adolescents presenting after a suicide attempt	Boys and girls differed in the methods of suicide attempt and the presence of a previous suicide attempt
Levinson, 2006	A national ED database, Israel	1996-2002	25,446	ED admissions classified as attempted suicide	Women do not attempt suicide more than men, except for 13-26 year olds.
Porter, 2016	PED in Spain	2010-2011	328 ED visits	Patients <15 years attending the pediatric emergency department who required	Males and females differ in disease presentation

				consultation to psychiatry service	
Rhodes, 2008	All hospital emergency departments in Ontario, Canada	April 1, 2001–March 31, 2002	+/- 2400 ED visits	All medicinal Self-Poisoning presentations	Females presented more often with poisoning.
Tonezer, 2015	Emergency Psychiatric Consultation (EPC) Service of a Brazilian hospital	2010-2013	259 ED visits	Patients <18 years who were assessed by a psychiatric emergency team	Females and males differ in the rates of psychiatric presentations and diagnoses.<br>Males needed more psychiatric medication during evaluation, but there was no difference in the rate of psychiatric hospitalization.

Table 1.2 Asthma (n=5)

**Author, year**	**Setting**	**Period**	**n**	**Inclusion**	**Conclusion**
Everage, 2010	Rhode Island Emergency Department Data	2005-2008	-	Every ED visit and hospitalization in Rhode Island to a non-federal hospital facility	Overall, women had higher rates of ED visits for asthma than men, except in the age group 0-4years
Fagan 2011	Schools in a Mississippi River community in Illinois	1994	2,693 children	Seventh through twelfth grade students	Female students reported significantly more asthma and allergic symptoms, and reported more severe symptoms and a greater number of emergency room and hospital admissions
Rosychuk, 2017	EDs in Alberta, Canada	1999-2011	115,853	Patients discharged from EDs for acute asthma	Prevalence of asthma in childhood is significantly higher in boys than girls with a higher rate of ED return. There was no difference in follow-up physician visits after ED discharge but time to first physician follow-up was shorter for girls.
Schatz, 2003	Computerized data from Southern California Kaiser-Permanente	1999 - 2000	60,694 subjects	Any patient aged 2 to 64 years, identified as having asthma in a large database	Asthma utilization and severity appear greater in males aged 2 to 13 years, somewhat greater in females aged 14 to 22 years.

Schatz, 2004	140 emergency departments (EDs)	Several periods between 1997 and 2001	1,602	Patients aged 2–13 years who presented to the emergency department (ED) with acute asthma	No material differences exists in acute presentation, chronic asthma characteristics, ED treatment, or ED course in boys compared to girls.

Table 1.3 Urinary tract infection (n=1)

Author, year	Setting	Period	n	Inclusion	Conclusion
Lo, 2013	ED of a general hospital, Brazil	2010	291	Patients younger than 15 years old who had clinical suspicion of UTI and collected quantitative urine culture.	Proteus mirabilis was significantly more prevalent in boys than girls

Table 1.4 Injuries and trauma, including (sexual) abuse (n=8)

Author, year	Setting	Period	n	Inclusion	Conclusion
Greenes, 2001	1) The National Pediatric Trauma Registry (NPTR)<br>2) A prospective cohort in an urban pediatric emergency department (ED)	1990-1999 (NTPR database)	1072 (NTPR database), 88 (cohort)	Patients ≤90 days of age who had been diagnosed with unintentional head trauma	Boys outnumber girls among infants less than 3 months of age with unintentional head trauma. These young infants appear to be passive participants in their injuries, which indicates that differences in parenting practices may account for the observed gender differences.
Hassan, 2015	ED of an urban Midwestern research and teaching hospital	2006-2010	95 children	Children 6 – 14 years old who were treated for alleged sexual abuse at the ED	There are significant differences in type of sexual abuse.
Irie, 2012	Data from 14 regional and tertiary public	2005-2008	282	Patients <= 17 years who met the inclusion criteria for the	Adolescent males were at a significantly higher risk of sustaining assault-related injuries.

	hospitals in Queensland			Queensland Trauma Registry (QTR) and were admitted to hospital for 24 h or more for the acute treatment of an accidental or assault-related injury	
Love, 2009	PED	June 2005- June 2008	3436 visits for falls of which 171 TBI's	ED visits for all injuries treated in the pediatric emergency department in which the word "fall" was listed as part of the chief complaint.	Females have a higher risk of TBI from falls than males.
Matteucci, 2007	24 regional poison centers	2002-2004	204	Pediatric snakebite injuries requiring antivenom therapy	Male children were more likely than female children to suffer Crotaline snakebites that required antivenom therapy.
McQuillan, 2006	Monklands Hospital Accident and Emergency (A&E) Department	1996–1999	19,483	0–17 year old residents of Airdrie and Coatbridge, Lanarkshire, Scotland, who attended the A&E Department with a home/leisure injury	The M:F RRR for non-sports A&E attendances remained constant throughout childhood , whilst that for sports attendances increased sharply with age The results suggest a dose–response relationship between sports participation and injury risk.
Preiss-Farzanegan, 2009	Regional trauma center emergency department.	February to September 2003	260	137 patients < 18 years presenting with sport-related mild TBI	Female minors are not at greater risk for post-concussion symptoms than males.
Ranney, 2011	Hurley Medical Center in Flint, Michigan, an inner-city, Level I trauma center ED	?	190	adolescents aged 14 to 18 years presenting to an urban ED with acute assault-related injury	Male and female adolescents with acute assault-related injuries were very similar. There was a greater prevalence of some risk factors among adolescent females.

Table 1.5 General resource use (n=2)

Author, year	Setting	Period	n	Inclusion	Conclusion
Henneman, 2014	urban, academic ED, USA	2003-2009	523, 882 outpatient ED encounters	Billing and demographic data from all ED visits	Resource utilization and profitability by gender were similar
Siminski, 2008	All major New South Wales EDs	2005	-	All ED visits in the administrative database	The rates of both "potentially primary care" and non-"potentially primary care" attendances are higher among men than among women..

References

1. Greenes DS, Wigotsky M, Schutzman SA. Gender differences in rates of unintentional head injury in the first 3 months of life. *Ambul Pediatr* 2001;1(3):178-80.
2. Hassan M, Killion C, Lewin L, et al. Gender-related sexual abuse experiences reported by children who were examined in an emergency department. *Arch Psychiatr Nurs* 2015;29(3):148-54.
3. Irie F, Lang J, Kaltner M, et al. Effects of gender, indigenous status and remoteness to health services on the occurrence of assault-related injuries in children and adolescents. *Injury* 2012;43(11):1873-80.
4. Love PF, Tepas JJ, 3rd, Wludyka PS, et al. Fall-related pediatric brain injuries: the role of race, age, and sex. *J Trauma* 2009;67(1 Suppl):S12-5.
5. Matteucci MJ, Hannum JE, Riffenburgh RH, et al. Pediatric sex group differences in location of snakebite injuries requiring antivenom therapy. *J Med Toxicol* 2007;3(3):103-6.
6. McQuillan R, Campbell H. Gender differences in adolescent injury characteristics: a population-based study of hospital A&E data. *Public Health* 2006;120(8):732-41.
7. Preiss-Farzanegan SJ, Chapman B, Wong TM, et al. The relationship between gender and postconcussion symptoms after sport-related mild traumatic brain injury. *Pm R* 2009;1(3):245-53.
8. Ranney ML, Whiteside L, Walton MA, et al. Sex differences in characteristics of adolescents presenting to the emergency department with acute assault-related injury. *Acad Emerg Med* 2011;18(10):1027-35.
9. Foto-Ozdemir D, Akdemir D, Cuhadaroglu-Cetin F. Gender differences in defense mechanisms, ways of coping with stress and sense of identity in adolescent suicide attempts. *Turk J Pediatr* 2016;58(3):271-81.
10. Kupferschmid S, Gysin-Maillart A, Buhler SK, et al. Gender differences in methods of suicide attempts and prevalence of previous suicide attempts. *Z Kinder Jugendpsychiatr Psychother* 2013;41(6):401-5.
11. Levinson D, Haklai Z, Stein N, et al. Suicide attempts in israel: age by gender analysis of a national emergency departments database. *Suicide Life Threat Behav* 2006;36(1):97-102.
12. Porter M, Gracia R, Oliva JC, et al. Mental Health Emergencies in Paediatric Services: Characteristics, Diagnostic Stability and Gender differences. *Actas Esp Psiquiatr* 2016;44(6):203-11.

13. Rhodes AE, Khan S, Boyle MH, et al. Sex differences in suicides among children and youth: the potential impact of help-seeking behaviour. *Can J Psychiatry* 2013;58(5):274-82.
14. Tonezer J, Muller T, Rocha GP, et al. Clinical Profile and Sex Differences in Brazilian Children and Adolescents Receiving Psychiatric Services in the Emergency Department. *Pediatr Emerg Care* 2015
15. Everage NJ, Pearlman DN, Sutton N, et al. Disparities by race/ethnicity and sex: asthma hospitalizations and emergency department visit rates in Rhode Island and Healthy People 2010 goals. *Med Health R I* 2010;93(6):177-8, 81-3.
16. Fagan JK, Scheff PA, Hryhorczuk D, et al. Prevalence of asthma and other allergic diseases in an adolescent population: association with gender and race. *Ann Allergy Asthma Immunol* 2001;86(2):177-84.
17. Rosychuk RJ, Ospina M, Zhang J, et al. Sex differences in outcomes after discharge from Alberta emergency departments for asthma: A large population-based study. *J Asthma* 2017:1-9.
18. Schatz M, Camargo CA, Jr. The relationship of sex to asthma prevalence, health care utilization, and medications in a large managed care organization. *Ann Allergy Asthma Immunol* 2003;91(6):553-8.
19. Schatz M, Clark S, Emond JA, et al. Sex differences among children 2-13 years of age presenting at the emergency department with acute asthma. *Pediatr Pulmonol* 2004;37(6):523-9.
20. Henneman PL, Nathanson BH, Ribeiro K, et al. The impact of age and gender on resource utilization and profitability in ED patients seen and released. *Am J Emerg Med* 2014;32(10):1159-67.
21. Siminski P, Bezzina AJ, Lago LP, et al. Primary care presentations at emergency departments: rates and reasons by age and sex. *Aust Health Rev* 2008;32(4):700-9.
22. Lo DS, Shieh HH, Ragazzi SL, et al. Community-acquired urinary tract infection: age and gender-dependent etiology. *J Bras Nefrol* 2013;35(2):93-8.

APPENDIX 2. Description of the different study sites

	**Erasmus MC, Rotterdam, the Netherlands**	**Maasstad Hospital, Rotterdam, the Netherlands**	**St Mary's Hospital, London, United Kingdom**	**Hospital Fernando da Fonseca, Lisbon, Portugal**	**General Hospital, Vienna, Austria**
Hospital characteristics	University hospital<br>60 paediatric beds	Teaching hospital<br>59 paediatric beds	University hospital<br>46 paediatric beds	Community hospital<br>91 paediatric beds	University hospital<br>134 paediatric beds
Catchment area	Urban<br>Mixed high and low socio-economic status	Urban<br>Generally low socio-economic status	Urban<br>Mixed high and low socio-economic status	Mixed urban and rural<br>Generally low socio-economic status	Urban<br>Mixed high and low socio-economic status
Emergency department characteristics	Before October 2014: Paediatric only<br>6500 children/year<br>Major trauma service in separate department	Mixed adult-paediatric<br>9500 children/year<br>Minor trauma service	Paediatric only<br>27,000 children/year<br>Major trauma service	Paediatric only<br>60,000 children/year<br>Major trauma service	Paediatric only<br>22,000 children/year<br>Major trauma service in separate department
Inclusion period	01-01-2012 to 31-12-2014	01-05-2014 to 01-11-2015	01-07-2014 to 28-02-2015	01-03-2014 to 28-02-2015	01-01-2014 to 31-12-2014
Study population	n = 18,594 (16%)	n = 10,584 (9%)	n = 15,556 (13%)	n = 53,175 (45%)	n = 20,300 (18%)

APPENDIX 3. Distinction between clinical presentations based on selected Manchester Triage System flowchart

Clinical presentation	MTS Flowcharts
**Cardiac**	Chest pain, Palpitations
**Dermatological**	Rashes, Bites and stings, Burns and scalds, Abscesses and local infections, Wounds
**Ear, Nose and Throat**	Sore throat, Facial problems, Ear problems
**Gastrointestinal**	Abdominal pain in adults, Abdominal pain in children, Diarrhoea and vomiting, GI bleeding
**Neurologic or Psychiatric**	Headache, Fits, Collapsed adult, Behaving strangely, Overdose and poisoning, Mental illness, Self-harm, Apparently drunk
**Respiratory**	Asthma, Shortness of breath in adults, Shortness of breath in children
**Trauma or muscular**	Limb problems, Major trauma, Neck pain, Back pain, Torso injury, Falls, Assault, Head injury, Limping child
**General malaise**	Unwell adult, Unwell child, Irritable child, Worried parent, Crying baby
**Uro- or gynaecological**	Urinary problems, Testicular pain, PV bleeding, Sexually acquired infection, Pregnancy
**Other**	Major incidents primary, Dental problems, Exposure to chemicals, Foreign body, Diabetes, Eye problems, Allergy, General/Other

The classification in subgroups of clinical presentation according to a previously published study.[1] Based on clinical experience, the flowcharts “Crying baby” and “Worried parent” were moved from the category Other to General malaise.

[1]*Zachariasse JM, Seiger N, Rood PP, et al. Validity of the Manchester Triage System in emergency care: A prospective observational study. PLoS One. 2017;12(2):e0170811*

Zachariasse et al. 2020

APPENDIX 4. Gender differences stratified by triage urgency

Figure 4.1 Differences between boys and girls over the different age categories, stratified by MTS urgency

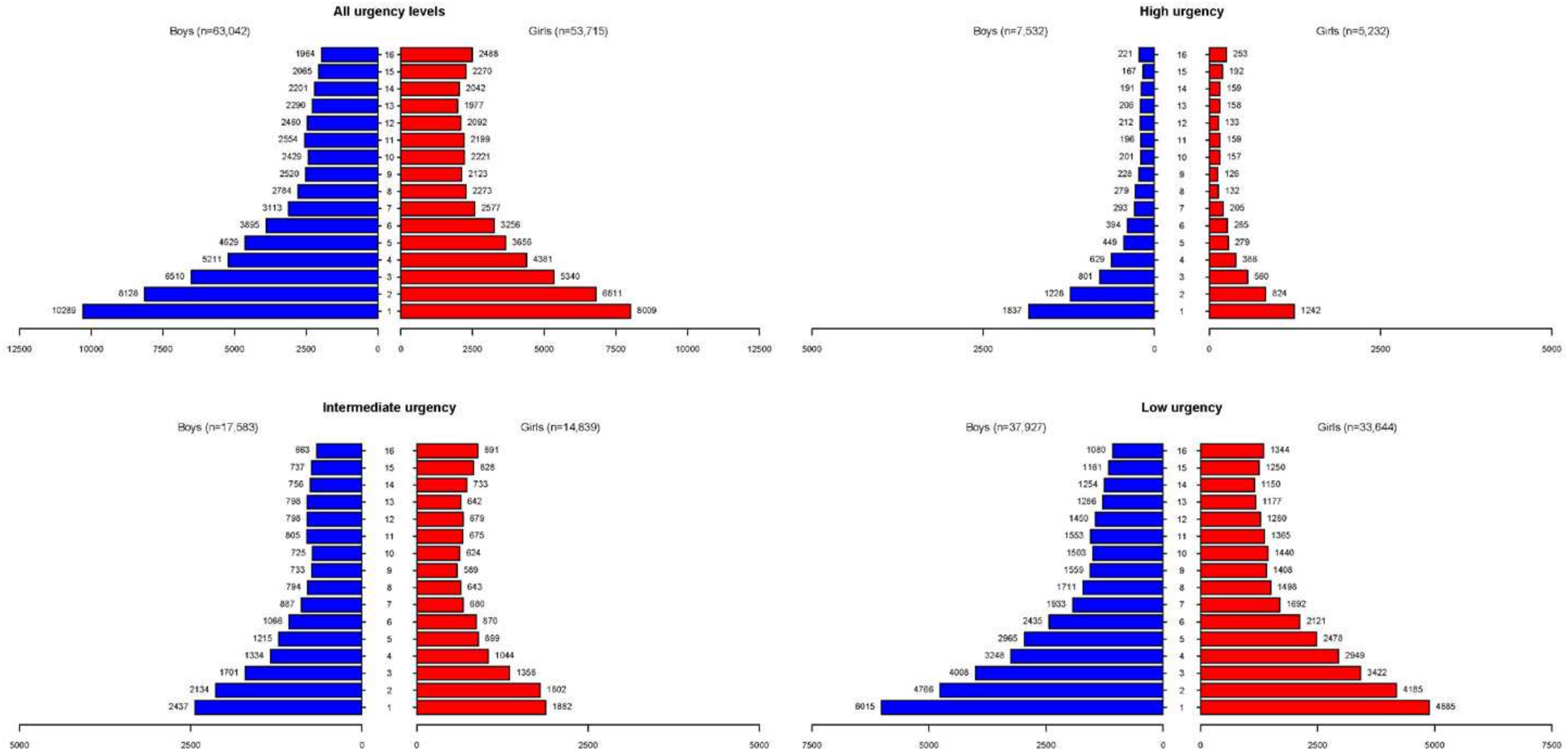

Zachariasse et al. 2020

Figure 4.2 Differences between boys and girls over the different clinical presentations, stratified by MTS urgency

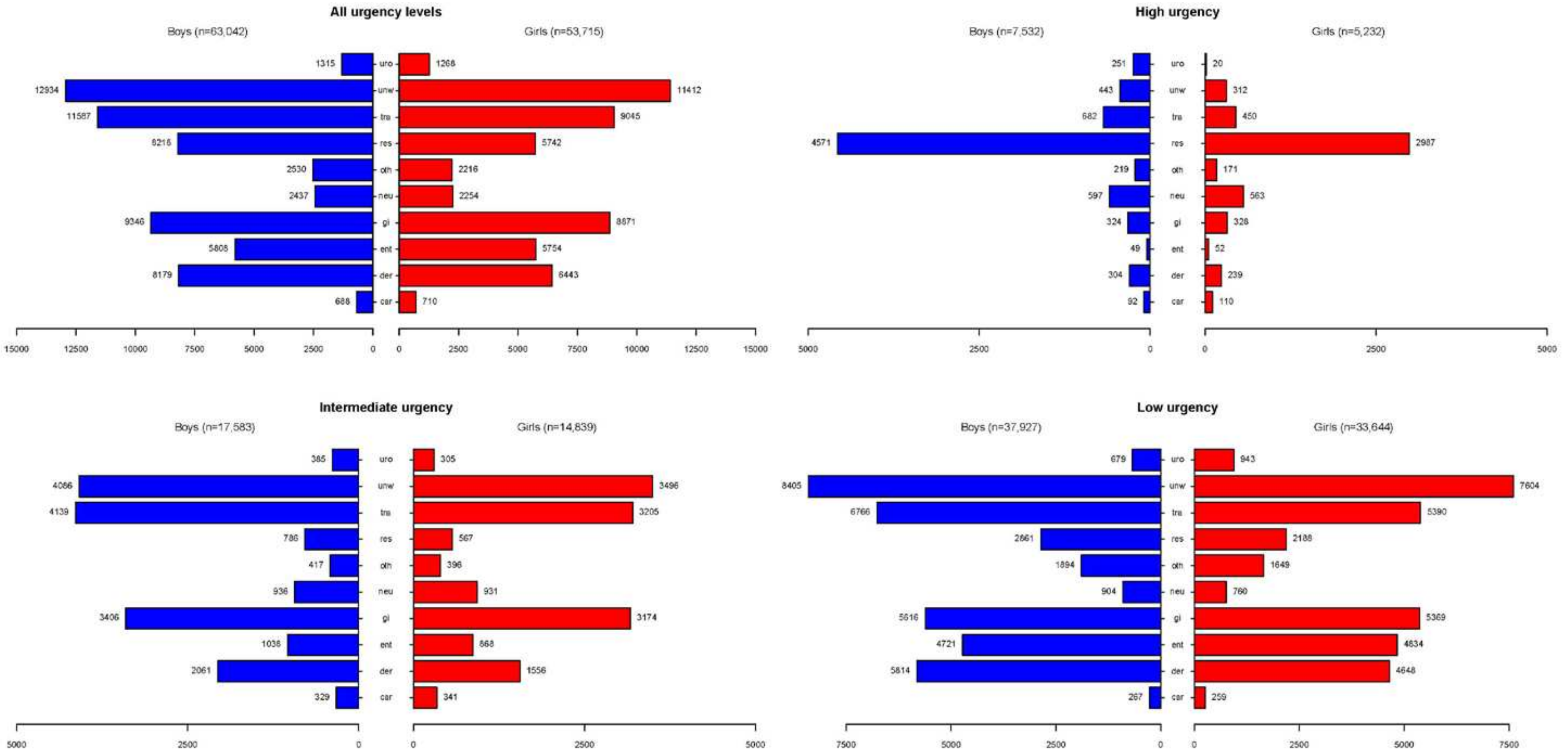

car=cardiac; der=dermatological; ent=ear, nose and throat; gi=gastrointestinal; neu=neurologic or psychiatric; oth=other; res=respiratory; tra=trauma or musculoskeletal; unw=general malaise, uro=uro- or gynaecological

Zachariasse JM, *et al. BMJ Open* 2020; 10:e035918. doi: 10.1136/bmjopen-2019-035918

Zachariasse et al. 2020

APPENDIX 5. Associations of sex with ED diagnostics and management in subgroups

Figure 5.1 Associations of sex with ED diagnostics and management in patients with trauma or musculoskeletal problems, adjusted for age and triage urgency (boys as reference group)

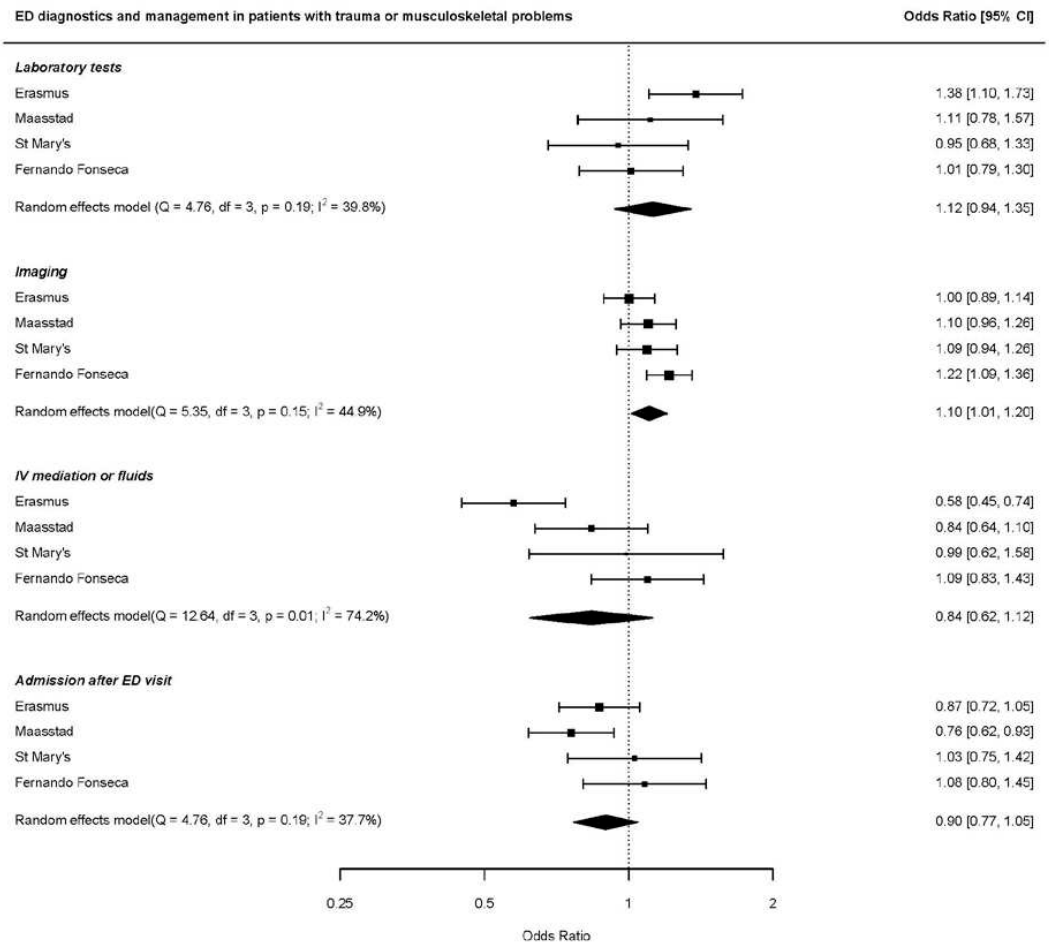

Zachariasse et al. 2020

Figure 5.2 Associations of sex with ED diagnostics and management in patients with gastrointestinal problems, adjusted for age and triage urgency (boys as reference group)

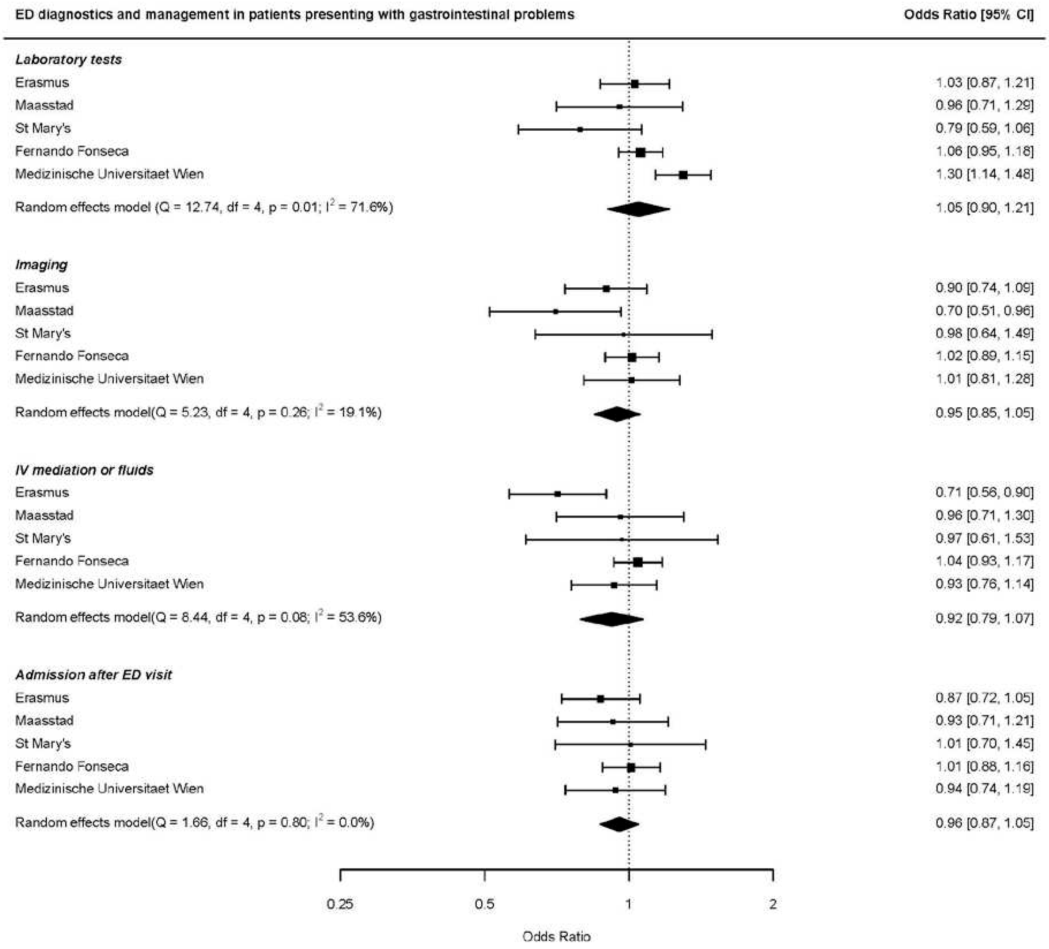

Zachariasse et al. 2020

Figure 5.3 Associations of sex with ED diagnostics and management in patients with respiratory problems, adjusted for age and triage urgency (boys as reference group)

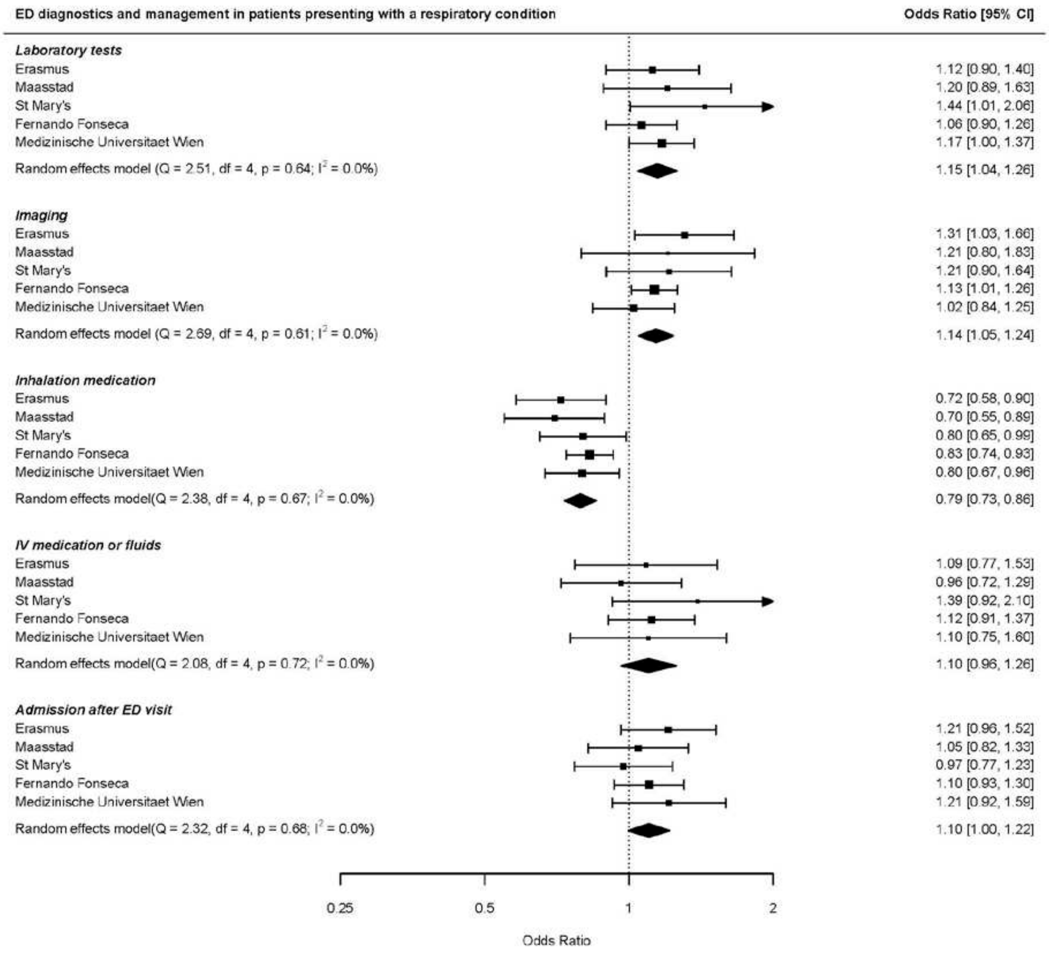

Zachariasse et al. 2020

Figure 5.4 Associations of sex with ED diagnostics and management in patients with fever, adjusted for age and triage urgency (boys as reference group)

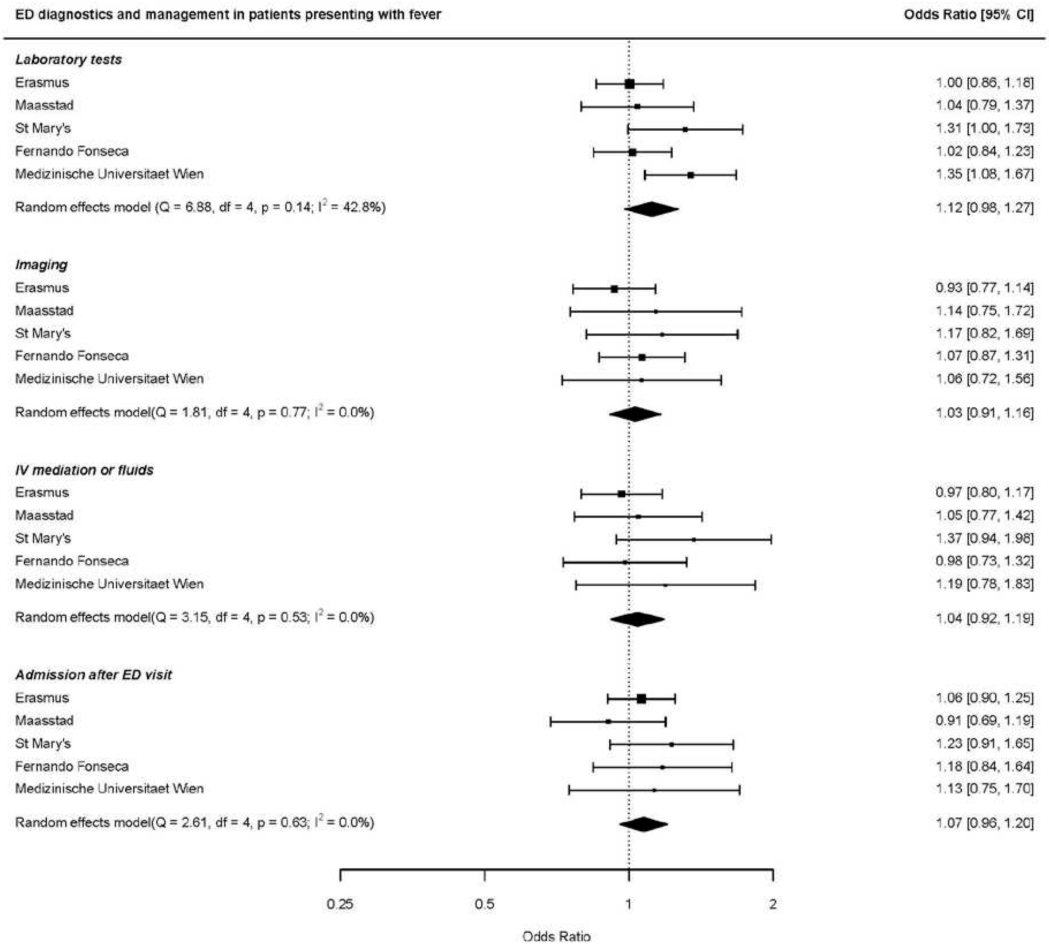

Supplement: Supplementary data [file bmjopen-2019-035918supp001.pdf]
