# Supplementary material for: Sex-specific differences in children attending the emergency department: prospective observational study
Source: BMJ Open. 2020 Sep 17;10(9):e035918. doi: 10.1136/bmjopen-2019-035918 (PMC7500294; doi:10.1136/bmjopen-2019-035918)
Supplement: Author's manuscript [file bmjopen-2019-035918.draft_revisions.pdf]

BMJ Open

BMJ Open is committed to open peer review. As part of this commitment we make the peer review history of every article we publish publicly available.

When an article is published we post the peer reviewers' comments and the authors' responses online. We also post the versions of the paper that were used during peer review. These are the versions that the peer review comments apply to.

The versions of the paper that follow are the versions that were submitted during the peer review process. They are not the versions of record or the final published versions. They should not be cited or distributed as the published version of this manuscript.

BMJ Open is an open access journal and the full, final, typeset and author-corrected version of record of the manuscript is available on our site with no access controls, subscription charges or pay-per-view fees (http://bmjopen.bmj.com).

If you have any questions on BMJ Open's open peer review process please email info.bmjopen@bmj.com

BMJ Open

Sex-Specific Differences in Children Attending the Emergency Department: Prospective Observational Study

Journal:	*BMJ Open*
Manuscript ID	bmjopen-2019-035918
Article Type:	Original research
Date Submitted by the Author:	26-Nov-2019

Complete List of Authors:	Zachariasse, Joany; Erasmus MC- Sophia Children's Hospital, Department of General Paediatrics<br>Borensztajn, Dorine; Erasmus MC Sophia Children Hospital, General Paediatrics<br>Nieboer, Daan; Erasmus MC - University Medical Center Rotterdam, Public Health<br>Alves, Claudio; Hospital Fernando Fonseca, General paediatrics, Emergency Unit<br>Greber-Platzer, Susanne; Medical University of Vienna, Department of Pediatrics and Adolescent Medicine<br>Keyzer-Dekker, Claudia; Erasmus MC- Sophia Children's Hospital, Department of Pediatric Surgery<br>Maconochie, Ian; Department of Paediatric Emergency,<br>Steyerberg, Ewout; Leiden University Medical Center, Department of Medical Statistics<br>Smit, Frank; Maasstad Ziekenhuis, Department of paediatrics<br>moll, Henriette; Erasmus University Medical Center Sophia Children�s hospital, Paediatrics

Keywords:	EPIDEMIOLOGY, Paediatric A&E and ambulatory care < PAEDIATRICS, PAEDIATRICS

SCHOLARONE™ Manuscripts

I, the Submitting Author has the right to grant and does grant on behalf of all authors of the Work (as defined in the below author licence), an exclusive licence and/or a non-exclusive licence for contributions from authors who are: i) UK Crown employees; ii) where BMJ has agreed a CC-BY licence shall apply, and/or iii) in accordance with the terms applicable for US Federal Government officers or employees acting as part of their official duties; on a worldwide, perpetual, irrevocable, royalty-free basis to BMJ Publishing Group Ltd ("BMJ") its licensees and where the relevant Journal is co-owned by BMJ to the co-owners of the Journal, to publish the Work in this journal and any other BMJ products and to exploit all rights, as set out in our licence.

The Submitting Author accepts and understands that any supply made under these terms is made by BMJ to the Submitting Author unless you are acting as an employee on behalf of your employer or a postgraduate student of an affiliated institution which is paying any applicable article publishing charge ("APC") for Open Access articles. Where the Submitting Author wishes to make the Work available on an Open Access basis (and intends to pay the relevant APC), the terms of reuse of such Open Access shall be governed by a Creative Commons licence – details of these licences and which Creative Commons licence will apply to this Work are set out in our licence referred to above.

Other than as permitted in any relevant BMJ Author's Self Archiving Policies, I confirm this Work has not been accepted for publication elsewhere, is not being considered for publication elsewhere and does not duplicate material already published. I confirm all authors consent to publication of this Work and authorise the granting of this licence.

For peer review only

Sex-Specific Differences in Children Attending the Emergency Department: Prospective Observational Study

Joany M Zachariasse, Dorine M Borensztajn, Daan Nieboer, Claudio F Alves, Susanne Greber-Platzer, Claudia M G Keyzer – Dekker, Ian K Maconochie, Ewout W Steyerberg, Frank J Smit, Henriëtte A Moll

Author affiliations:

Department of General Paediatrics, Erasmus MC- Sophia Children's Hospital, University Medical Centre Rotterdam, PO Box 2060, 3000 CB Rotterdam, The Netherlands

Joany M Zachariasse

PhD-student

Department of General Paediatrics, Erasmus MC- Sophia Children's Hospital, University Medical Centre Rotterdam, PO Box 2060, 3000 CB Rotterdam, The Netherlands

Dorine M Borensztajn

Paediatrician

Department of Public Health, Erasmus MC-University Medical Center Rotterdam, P.O. Box 2040

3000 CA Rotterdam The Netherlands

Daan Nieboer

PhD-student

Department of Paediatrics, Emergency Unit, Hospital Professor Doutor Fernando da Fonseca, EPE, IC 19, 2720-276 Amadora, Lisbon, Portugal

Claudio F Alves

Paediatrician

For peer review only

Medical University Vienna, Department of Pediatrics and Adolescent Medicine, Währinger Gürtel 16, 1090, Vienna, Austria

Susanne Greber-Platzer

Professor

Department of Pediatric Surgery, Erasmus MC-University Medical Center Rotterdam, PO Box 2060, 3000 CB Rotterdam, The Netherlands

Claudia M G Keyzer - Dekker

Paediatric Surgeon

Department of Paediatric Emergency Medicine, Imperial College NHS Healthcare Trust, London, United Kingdom

Ian K Maconochie

Associate professor

Department of Medical Statistics and Bioinformatics, Leiden University Medical Center, the Netherlands

and

Department of Public Health, Erasmus MC-University Medical Center Rotterdam, P.O. Box 2040, 3000 CA Rotterdamm The Netherlands

Ewout Steyerberg

Professor

Department of Paediatrics, Maasstad Hospital, P.O. Box 9100, 3007 AC Rotterdam, The Netherlands

Frank J Smit

Paediatrician

For peer review only

Department of General Paediatrics, Erasmus MC- Sophia Children's Hospital, University Medical Centre Rotterdam, PO Box 2060, 3000 CB Rotterdam, The Netherlands

Henriëtte A Moll

Professor

Corresponding author:

Prof. Dr. Henriëtte A. Moll

h.a.moll@erasmusmc.nl

Keywords [MeSH]: sex, emergency medicine, child, epidemiology, prospective studies

Word count: 1801

Word count abstract: 206

Abbreviations:

CI - confidence interval

ED – emergency department

IOM – Institute of Medicine

MTS – Manchester Triage System

OR – odds ratio

Funding: This research received no specific grant from any funding agency in the public, commercial or not-for-profit sectors

Ethical approval: The research protocol was reviewed and approved by the local medical ethics committees and requirement for informed consent was waived.

Competing interests: The authors declare they have no potential conflicts of interest to disclose.

For peer review only

Contributorship statement: JZ contributed to the conceptualization and design of the study, carried out the analyses, and drafted the initial manuscript. DB, FS, CA, CK, IM, and SG contributed to the conceptualization and design of the study, coordinated the data collection, contributed to interpretation of the data, and critically reviewed the manuscript. DN and ES contributed to the conceptualization and design of the study, supervised the analyses, and critically reviewed the manuscript. HM contributed to the conceptualization and design of the study, contributed to interpretation of the data, critically reviewed the manuscript and supervised the study. All authors approved the final manuscript as submitted and agree to be accountable for all aspects of the work.

Transparency declaration: The lead author affirms that this manuscript is an honest, accurate, and transparent account of the study being reported; that no important aspects of the study have been omitted; and that any discrepancies from the study as planned have been explained.

Copyright licence: The Corresponding Author has the right to grant on behalf of all authors and does grant on behalf of all authors, a worldwide licence to the Publishers and its licensees in perpetuity, in all forms, formats and media (whether known now or created in the future), to i) publish, reproduce, distribute, display and store the Contribution, ii) translate the Contribution into other languages, create adaptations, reprints, include within collections and create summaries, extract and/or, abstracts of the Contributions, iii) create any other derivative work(s) based on the Contribution, iv) to exploit all subsidiary rights in the Contribution, v) the inclusion of electronic links from the Contribution to third party material where-ever it may be located; and vi) licence any third party to do any or all of the above.

Dating sharing: Data from this study are available upon request to the corresponding author of the study (h.a.moll@erasmusmc.nl), subject to local rules and regulations.

Acknowledgements: We gratefully acknowledge Rikke Jorgensen, paediatric research nurse (St. Mary's Hospital, London) and Pinky Rose Espina, paediatric resident (General Hospital, Vienna) for their assistance with data collection for this study.

For peer review only

Abstract

Objective To assess the role of sex in the presentation and management of children attending the emergency department.

Design The TrIAGE project, a prospective observational study based on curated electronic health record data

Setting Five diverse European hospitals in four countries (Austria, Netherlands, Portugal, United Kingdom)

Participants All consecutive paediatric emergency department visits of children under the age of 16

Main outcome measures The association between sex (male or female) and diagnostic tests and disease management in children presenting with medical problems, and trauma and injuries. Results from the different hospitals were pooled in a random effects meta-analysis.

Results 116,440 children were included in the study. Boys accounted for the majority of medical visits (n=48,179, 53%), except in adolescence, and for the majority of trauma or injury presentations in all age groups (n=14,537, 57%). After adjusting for age and triage urgency, girls had more laboratory tests when presenting with medical problems, and more imaging when presenting with trauma. Boys attending the emergency department were more often treated with inhalation medication. There was no difference in hospital admission between the sexes.

Conclusion In childhood, sex is associated with differences in disease presentation and management. Further research is needed to investigate which biological or sociocultural factors are responsible.

For peer review only

Strengths and limitations of the study

- The present study was based on a large, multicentre and international cohort of more than 100,000 emergency department visits

- The study is the first to assess the role of sex in the prevalence and type of clinical presentations and in the diagnostics and management of children attending the emergency department

- Due to the use of standardized, routinely collected data, only general patterns and broad subgroups of patients could be evaluated

- Only two hospitals in our study included patients with major trauma, which limits generalizability to this subgroup of patients

For peer review only

Introduction

There is increasing evidence that differences between men and women are important in the epidemiology, pathophysiology, treatment and outcome of many diseases.[1-3] The Institute of Medicine (IOM) has emphasized that sex is an important variable that should be considered in biomedical and health related research.[4] Sex refers to the biological differences between men and women, while gender refers to a broader concept including social and cultural distinctions associated with a given sex.[3]

In emergency medicine, several sex and gender specific differences have been found in adults but research in children is scarce.[2 5-7] Only twenty-two relevant articles described sex or gender differences in children related to emergency medicine between January 2000 and November 2017, according to PubMed. These studies found differences between boys and girls in presentations of trauma and injuries, mental health related conditions, and asthma. (Appendix 1).

The aim of this study is to assess the role of sex in the clinical presentation, diagnostics and management of children attending the emergency department (ED).

For peer review only

Methods

This study was embedded in the TrIAGE project, a prospective observational study, based on electronic health record data, in five EDs in four European countries (the Netherlands, United Kingdom, Austria, Portugal). The study was approved by the medical ethical committees of the participating institutions. All waived the requirement for informed consent.

Study settings and patient population

In the TrIAGE study, 119,209 consecutive ED visits of children under the age of 16 years were included. Enrolment took place during a period of 8 to 36 months between 2012 and 2015 in five diverse study sites (Appendix 2). We restricted our analysis to children with complete data, thereby excluding 1771 children (1.5%) with missing triage categories or missing presenting problem. The General Hospital Vienna only included children attending with a medical complaint.

Data collection

The TrIAGE project is based on routinely collected, standardized electronic health record data. At the start of the study, a set of minimally required variables was determined. Completeness and accuracy of the data was discussed during a site visit and telephone meetings using a checklist quality control. With posters, newsletters and presentations, nurses were encouraged to complete the full medical records including vital sign measurements.

During the study period, nurses and physicians entered the clinical data in the medical or nursing records. After data extraction, careful data harmonization and quality checks were performed.

Determinants

In all participating hospitals, sex (male or female) was routinely registered for each patient as part of the administrative process. We distinguished children presenting with general medical problems and children presenting with trauma or injuries, based on the main problem presented during triage. All

For peer review only

participating hospitals used the Manchester Triage System (MTS). This triage system requires the triage nurse to select a flowchart for each patient, representing the chief complaint. (Appendix 3).

Age and triage urgency were considered as potential confounding variables in the relationship between sex and disease management. Age was maintained as a continuous variable. Triage urgency was defined as the MTS urgency category and placed into three groups: high urgent (MTS category immediate or high urgent), urgent and low urgent (standard or non-urgent). Three hospitals (Erasmus MC, General Hospital Vienna and Hospital Fernando da Fonseca) implemented MTS modifications. To ensure consistency among hospitals, the urgency levels according to MTS version 3 were modelled in all hospitals[8 9]

Data analysis

In descriptive analyses, differences between boys and girls in the type and severity of problems with which they presented at the ED were explored.

Multivariable logistic regression models were used to assess the association between sex and diagnostics (laboratory tests or imaging) and disease management (inhalation or intravenous medication and hospital admission) for each study site. Analyses were adjusted for age and triage urgency and boys were determined as the reference group. The results of the individual hospitals were pooled in a random effects meta-analysis and presented in forest plots. ORs were considered statistically significant when the 95% confidence interval (CI) did not include 1. Heterogeneity among settings was assessed using the Cochran's Q statistic and the I^2 statistic.

We performed subgroup analyses of two pre-specified subgroups: children presenting with fever and children presenting with shortness of breath. Fever was defined as either a temperature of ≥ 38.5°C at the ED or the item "fever" selected as triage discriminator. Shortness of breath was defined based on the triage flowchart.

Statistical analyses were performed in SPSS Statistics version 21.0 (IBM Corp, Armonk, NY, USA) and R version 3.4.2 (R Foundation, Vienna, Austria). The package metafor was used to conduct the random effects meta-analysis and create forest plots.

For peer review only

Patient and public involvement

There was no patient involvement in the design of the study or the writing of the manuscript. We plan to disseminate the results to patient organisations, clinicians and policy makers working in emergency care upon publication.

For peer review only

Results

In total, 116,440 children were included in the study of whom 25,434 (22%) presented with trauma or injuries and 90,738 (78%) with general medical problems. In both groups, boys constituted the majority of visits (Appendix 4).

Sex-specific differences in general medical problems

In the group of children presenting with general medical problems the sex imbalance in number of ED visits changed with age. In childhood, consistently more boys than girls attended the ED, and the ratio reversed in adolescence (Appendix 5). Boys accounted for the majority of visits in all triage urgency levels, most evidently in the high urgency categories. They had the highest proportion of ED visits with chief complaint shortness of breath, while in children presenting with abdominal pain, the boy to girl ratio was almost equal (Table 1).

Adjusted for age and triage urgency, girls had more laboratory tests at the ED than boys (pooled OR 1.12, 95% CI 1.08-1.18). Boys, on the other hand, received more inhalation medication (pooled OR 0.76, 95% CI 0.70-0.83). The same trend was seen in the subgroups of patients presenting with fever and shortness of breath, although heterogeneity was observed (Appendix 6). There was no difference between sexes in imaging performed at the ED, IV medication given or disposition after the ED visit (Figure 1).

Results were consistent among the 5 hospitals and heterogeneity was limited. There was some evidence for heterogeneity in the use of inhalation medication (Q-statistic 8.04, p=0.09, I^2 statistic 49%) but the direction of the association was the same in all settings and odds ratios all indicated a clinically relevant difference.

Sex-specific differences in trauma and injuries

In children presenting with trauma and injuries, the difference between sexes was small under the age of 1 year, but substantial in children up to 12 years of age and in adolescents (Table 1, Appendix 5). Boys represented the majority across all triage urgency levels, and in all types of presenting problems, but particularly in the subgroups of children presenting with head injuries and wounds.

For peer review only

Adjusted for age and triage urgency, more imaging was performed in girls compared with boys (pooled OR 1.16, 95% CI 1.07-1.25). There was no difference between boys and girls in the use of laboratory tests or IV medication at the ED or hospital admission after the ED visit (Figure 2).

Results were more variable than in the general medical ED visits with some evidence of heterogeneity among the settings, particularly for imaging and IV medication or fluids. The association between sex and use of imaging, however, was consistently in the same direction, with ORs ranging from 1.05 to 1.24.

For peer review only

Discussion

In a European cohort of more than 100,000 ED visits, boys accounted for the majority of ED visits in trauma as well as medical presentations. In particular, a higher proportion of boys presented with shortness of breath, fever, and different types of trauma and injuries. In adolescence, the male to female ratio reversed. Girls had more laboratory tests when presenting with medical problems, and more imaging when presenting with trauma and injuries, when adjusted for age and triage urgency level. Boys, on the other hand, received more inhalation medication. There was no difference between sexes in the odds of hospital admission.

This is the first study that assesses the role of sex in paediatric emergency medicine, using a large, multicentre and international cohort. Despite the diversity in study sites, results were comparable across the participating EDs, supporting generalizability of the findings.

These results should be interpreted in light of the limitations of using standardized routinely collected data. Detailed information such as specific types of diagnoses or results from diagnostic tests was not available. Therefore, only general patterns and broad subgroups of patients were evaluated. Furthermore, it is possible that observed differences in diagnostics and management between boys and girls are confounded by sex-specific differences in type and severity of presenting conditions. The analyses were adjusted for age and triage urgency, and the findings were analysed in specific subtypes of presenting problems. There may, however, be remaining variation not captured in urgency and presenting problem. Finally, only two hospitals in our study included patients with major trauma which limits generalizability to this subgroup of patients.

The higher male attendance rate in European EDs is consistent with findings from the US National Hospital Ambulatory Medical Surveys.[10] Diverse biological explanations have been proposed, such as an increased susceptibility to infections and higher risk of lung disease in men, due to male sex hormones.[11-13] Behavioural, social and cultural factors might lead to differences in the number and types of ED visits as well. These may include differences in activities, risk taking behaviour, parenting practices, and different referral decisions by primary care providers.[14 15]

Beside sex differences in disease presentations, differences in diagnostics and management in the ED were observed. These could be caused by differences in disease severity, but may also be attributed to differences in clinical presentation. Boys and girls may differ regarding the presenting signs and symptoms, the way they express pain or distress, or their ability to phrase their symptoms. Diagnostic

For peer review only - http://bmjopen.bmj.com/site/about/guidelines.xhtml

uncertainty due to a-specific disease presentation may warrant more diagnostic tests, which could explain our observation that girls had more laboratory and imaging performed. Finally it is possible that provider attitude subconsciously differs in boys compared with girls. In adults, implicit gender bias in diagnostics and treatment has been found in observational research and standardized case scenarios. [16, 17]

In adults, greater awareness of sex-specific differences has changed medical practice and public-health recommendations. Better treatments and decreases in mortality in women from specific conditions including cardiovascular disease and certain cancers have been attributed to the greater understanding of women's health and investment in women's health research.[3] In children, the role of sex and gender on health is largely unknown and research assessing sex -specific differences is scare. Future studies should focus on the role of sex and gender in specific conditions and determine the influence of biological, as well as social and cultural factors.

For peer review only - http://bmjopen.bmj.com/site/about/guidelines.xhtml

References

1. Doyal L. Sex, gender, and health: the need for a new approach. *Bmj* 2001;323(7320):1061-3.
2. Greenberg MR, Safdar B, Choo EK, et al. Future directions in sex- and Gender-specific Emergency Medicine. *Acad Emerg Med* 2014;21(12):1339-42.
3. Institute of Medicine Committee on Women's Health Research. Women's Health Research: Progress, Pitfalls and Promise. 2010
4. Institute of Medicine Committee on Understanding the Biology of Sex and Gender Differences. Exploring the Biological Contributions to Human Health: Does Sex Matter? 2001
5. Mehta LS, Beckie TM, DeVon HA, et al. Acute Myocardial Infarction in Women: A Scientific Statement From the American Heart Association. *Circulation* 2016;133(9):916-47.
6. Wigginton JG, Perman SM, Barr GC, et al. Sex- and gender-specific research priorities in cardiovascular resuscitation: proceedings from the 2014 Academic Emergency Medicine Consensus Conference Cardiovascular Resuscitation Research Workgroup. *Acad Emerg Med* 2014;21(12):1343-9.
7. Raukar NP, Zonfrillo MR, Kane K, et al. Gender- and sex-specific sports-related injury research in emergency medicine: a consensus on future research direction and focused application. *Acad Emerg Med* 2014;21(12):1370-9.
8. Mackway-Jones K, Marsden J, Windle J. Emergency Triage: John Wiley & Sons, Ltd. 2014.
9. van Veen M, Steyerberg EW, Van't Klooster M, et al. The Manchester triage system: improvements for paediatric emergency care. *Emerg Med J* 2012;29(8):654-9.
10. Rui P, Okeyode T. National Ambulatory Medical Care Survey: 2015 State and National Summary tables. Available from: http://www.cdc.gov/nchs/ahcd/ahcd/products.htm. Accessed 11-06-2017.
11. Klein SL. The effects of hormones on sex differences in infection: from genes to behavior. *Neurosci Biobehav Rev* 2000;24(6):627-38.
12. Wichmann MW, Inthorn D, Andress HJ, et al. Incidence and mortality of severe sepsis in surgical intensive care patients: the influence of patient gender on disease process and outcome. *Intensive Care Med* 2000;26(2):167-72.
13. Carey MA, Card JW, Voltz JW, et al. It's all about sex: gender, lung development and lung disease. *Trends Endocrinol Metab* 2007;18(8):308-13.
14. Hartzog TH, Timerding BL, Alson RL. Pediatric trauma: enabling factors, social situations, and outcome. *Acad Emerg Med* 1996;3(3):213-20.
15. Greenes DS, Wigotsky M, Schutzman SA. Gender differences in rates of unintentional head injury in the first 3 months of life. *Ambul Pediatr* 2001;1(3):178-80.
16. Borkhoff CM, Hawker GA, Kreder HJ, et al. The effect of patients' sex on physicians' recommendations for total knee arthroplasty. *Cmaj* 2008;178(6):681-7.
17. Hawker GA, Wright JG, Coyte PC, et al. Differences between men and women in the rate of use of hip and knee arthroplasty. *N Engl J Med* 2000;342(14):1016-22.

For peer review only - http://bmjopen.bmj.com/site/about/guidelines.xhtml

Figure legends

Figure 1 Associations of sex with ED diagnostics and management in medical patients, adjusted for age and triage urgency (boys as reference group)

Figure 2 Associations of sex with ED diagnostics and management in trauma and injury patients**,** adjusted for age and triage urgency (boys as reference group)

For peer review only

Tables

Table 1 Sex-specific differences in general medical and trauma and injury patients

	Male (n=48,179)	Range over hospitals	Female (n=42,559)	Range over hospitals	Male (n=14,537)	Range over hospitals	Female (n=10,897)	Range over hospitals
	General medical				Trauma and injuries			
Age								
0 – < 1 y	9,741 (57%)	53%-61%	7,508 (44%)	39%-47%	520 (52%)	51%-54%	484 (48%)	46%-49%
1 – < 12 y	33,996 (54%)	52%-59%	29,419 (46%)	41%-48%	9,984 (58%)	56%-60%	7,299 (42%)	40%-44%
≥ 12 y	4,442 (44%)	43%-47%	5,632 (56%)	53%-57%	4,033 (56%)	53%-63%	3,114 (44%)	37%-47%
Triage urgency								
Emergent | Very urgent	6,741 (59%)	54%-64%	4,697 (41%)	36%-46%	775 (60%)	57%-63%	522 (40%)	37%-43%
Urgent	12,556 (53%)	50%-57%	11,121 (47%)	43%-50%	4,984 (57%)	56%-59%	3,689(43%)	41%-44%
Standard | Non-urgent	28,882 (52%)	50%-57%	26,741 (48%)	43%-50%	8,778 (57%)	55%-60%	6,686 (43%)	41%-45%
Presenting problem								
Fever	4,781 (55%)	51%-59%	3,922 (45%)	41%-49%	-	-	-	-
Shortness of breath	7,988 (59%)	57%-64%	5,563 (41%)	36%-43%	-	-	-	-

For peer review only - http://bmjopen.bmj.com/site/about/guidelines.xhtml

Abdominal pain	3,469 (49%)	45%-58%	3,653 (51%)	42%-55%	-	-	-	-
Vomiting and diarrhoea	5,770 (53%)	52%-56%	5,129 (47%)	44%-49%	-	-	-	-
Limb injury	-	-	-	-	7,858 (55%)	52%-58%	6,407 (45%)	42%-48%
Head injury	-	-	-	-	1,945 (64%)	61%-66%	1,091 (46%)	34%-39%
Wounds	-	-	-	-	2,061 (64%)	63%-66%	1,160 (36%)	34%-37%

Percentages are row totals

For peer review only

For peer review only - http://bmjopen.bmj.com/site/about/guidelines.xhtml

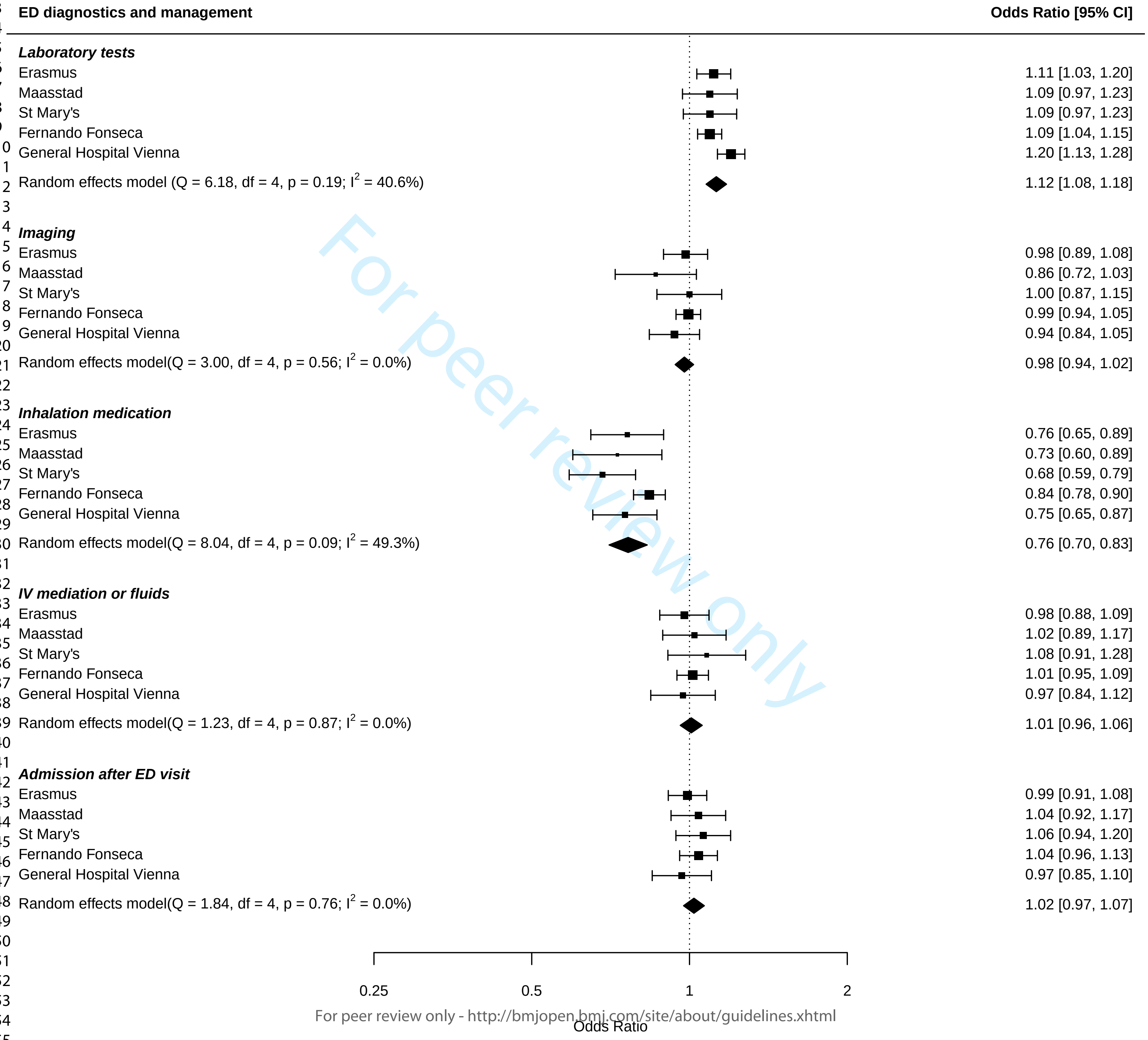

For peer review only

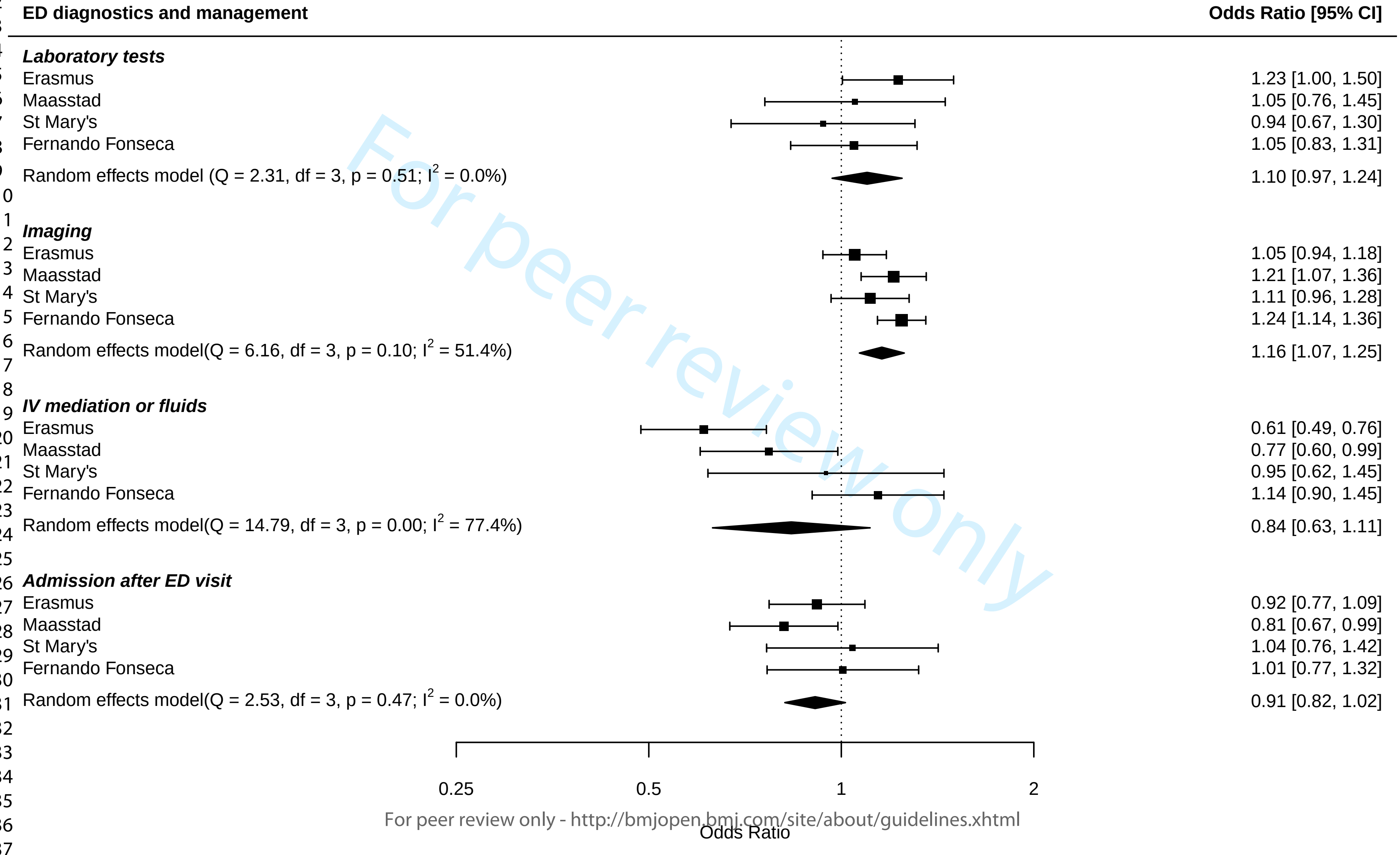

For peer review only

For peer review only - http://bmjopen.bmj.com/site/about/guidelines.xhtml

APPENDIX 1. PubMed literature search

We conducted a systematic literature search to identify articles describing any sex or gender differences in children visiting the emergency department (ED), including but not restricted to differences in presenting problem, disease presentation, disease severity, diagnostics, management and health care provider attitudes and behaviours.

We searched the Pubmed database for articles published from the year 2000 onwards, using search terms related to 1) age or gender (*gender [ti] or sex [ti]*); 2) emergency care (*emergency medicine [mh] or emergency service, hospital [mh] or emergency medical services [mh] or emergency department [tiab] or emergency room [tiab] or emergency medicine [tiab] or emergency service [tiab] or emergency services [tiab]*); and 3) paediatrics (*child [mh] OR infant [mh] OR adolescent [mh] OR Infant, Newborn [mh] OR child, preschool[mh] OR child* [ti] OR adolescent [ti] OR infant [ti] OR pediatr**)

This search identified a total of 134 papers of which 22 were included in the final selection. Papers were excluded because they did not (separately) address children (n=77), were not related to emergency medicine (n=27), were conducted in developing countries (n=3), or did not assess gender differences in patients (n=5).

Selected papers were divided into categories based on the research topic. The topics most commonly studied were identified: trauma and injuries including (sexual) assault (n=8)(1-8); mental health issues, including intoxications (n=6)(9-14) and asthma (n=5)(15-19). Other topics were general resource use (n=2)(20, 21) and urinary tract infections (n=1)(22).

References

1. Greenes DS, Wigotsky M, Schutzman SA. Gender differences in rates of unintentional head injury in the first 3 months of life. Ambul Pediatr. 2001;1(3):178-80.
2. Hassan M, Killion C, Lewin L, Totten V, Gary F. Gender-related sexual abuse experiences reported by children who were examined in an emergency department. Arch Psychiatr Nurs. 2015;29(3):148-54.
3. Irie F, Lang J, Kaltner M, Le Brocque R, Kenardy J. Effects of gender, indigenous status and remoteness to health services on the occurrence of assault-related injuries in children and adolescents. Injury. 2012;43(11):1873-80.
4. Love PF, Tepas JJ, 3rd, Wludyka PS, Masnita-Iusan C. Fall-related pediatric brain injuries: the role of race, age, and sex. J Trauma. 2009;67(1 Suppl):S12-5.
5. Matteucci MJ, Hannum JE, Riffenburgh RH, Clark RF. Pediatric sex group differences in location of snakebite injuries requiring antivenom therapy. J Med Toxicol. 2007;3(3):103-6.
6. McQuillan R, Campbell H. Gender differences in adolescent injury characteristics: a population-based study of hospital A&E data. Public Health. 2006;120(8):732-41.
7. Preiss-Farzanegan SJ, Chapman B, Wong TM, Wu J, Bazarian JJ. The relationship between gender and postconcussion symptoms after sport-related mild traumatic brain injury. Pm R. 2009;1(3):245-53.
8. Ranney ML, Whiteside L, Walton MA, Chermack ST, Zimmerman MA, Cunningham RM. Sex differences in characteristics of adolescents presenting to the emergency department with acute assault-related injury. Acad Emerg Med. 2011;18(10):1027-35.
9. Foto-Ozdemir D, Akdemir D, Cuhadaroglu-Cetin F. Gender differences in defense mechanisms, ways of coping with stress and sense of identity in adolescent suicide attempts. Turk J Pediatr. 2016;58(3):271-81.

For peer review only - http://bmjopen.bmj.com/site/about/guidelines.xhtml

10. Kupferschmid S, Gysin-Maillart A, Buhler SK, et al. Gender differences in methods of suicide attempts and prevalence of previous suicide attempts. Z Kinder Jugendpsychiatr Psychother. 2013;41(6):401-5.
11. Levinson D, Haklai Z, Stein N, Gordon ES. Suicide attempts in israel: age by gender analysis of a national emergency departments database. Suicide Life Threat Behav. 2006;36(1):97-102.
12. Porter M, Gracia R, Oliva JC, Pamias M, Garcia-Pares G, Cobo J. Mental Health Emergencies in Paediatric Services: Characteristics, Diagnostic Stability and Gender differences. Actas Esp Psiquiatr. 2016;44(6):203-11.
13. Rhodes AE, Khan S, Boyle MH, et al. Sex differences in suicides among children and youth: the potential impact of help-seeking behaviour. Can J Psychiatry. 2013;58(5):274-82.
14. Tonezer J, Muller T, Rocha GP, Recondo R, Nogueira EL, Spanemberg L. Clinical Profile and Sex Differences in Brazilian Children and Adolescents Receiving Psychiatric Services in the Emergency Department. Pediatr Emerg Care. 2015.
15. Everage NJ, Pearlman DN, Sutton N, Goldman D. Disparities by race/ethnicity and sex: asthma hospitalizations and emergency department visit rates in Rhode Island and Healthy People 2010 goals. Med Health R I. 2010;93(6):177-8, 81-3.
16. Fagan JK, Scheff PA, Hryhorczuk D, Ramakrishnan V, Ross M, Persky V. Prevalence of asthma and other allergic diseases in an adolescent population: association with gender and race. Ann Allergy Asthma Immunol. 2001;86(2):177-84.
17. Rosychuk RJ, Ospina M, Zhang J, Leigh R, Cave A, Rowe BH. Sex differences in outcomes after discharge from Alberta emergency departments for asthma: A large population-based study. J Asthma. 2017:1-9.
18. Schatz M, Camargo CA, Jr. The relationship of sex to asthma prevalence, health care utilization, and medications in a large managed care organization. Ann Allergy Asthma Immunol. 2003;91(6):553-8.
19. Schatz M, Clark S, Emond JA, Schreiber D, Camargo CA, Jr. Sex differences among children 2-13 years of age presenting at the emergency department with acute asthma. Pediatr Pulmonol. 2004;37(6):523-9.
20. Henneman PL, Nathanson BH, Ribeiro K, Balasubramanian H. The impact of age and gender on resource utilization and profitability in ED patients seen and released. Am J Emerg Med. 2014;32(10):1159-67.
21. Siminski P, Bezzina AJ, Lago LP, Eagar K. Primary care presentations at emergency departments: rates and reasons by age and sex. Aust Health Rev. 2008;32(4):700-9.
22. Lo DS, Shieh HH, Ragazzi SL, Koch VH, Martinez MB, Gilio AE. Community-acquired urinary tract infection: age and gender-dependent etiology. J Bras Nefrol. 2013;35(2):93-8.

For peer review only

For peer review only - http://bmjopen.bmj.com/site/about/guidelines.xhtml

Zachariasse et al. 2019

APPENDIX 2. Description of the different study sites

	**Erasmus MC, Rotterdam, the Netherlands**	**Maasstad Hospital, Rotterdam, the Netherlands**	**St Mary's Hospital, London, United Kingdom**	**Hospital Fernando da Fonseca, Lisbon, Portugal**	**General Hospital, Vienna, Austria**
Hospital characteristics	University hospital<br>60 paediatric beds	Teaching hospital<br>59 paediatric beds	University hospital<br>46 paediatric beds	Community hospital<br>91 paediatric beds	University hospital<br>134 paediatric beds
Catchment area	Urban<br>Mixed high and low socio-economic status	Urban<br>Generally low socio-economic status	Urban<br>Mixed high and low socio-economic status	Mixed urban and rural<br>Generally low socio-economic status	Urban<br>Mixed high and low socio-economic status
Emergency department characteristics	Before October 2014: Paediatric only<br>6500 children/year<br>Major trauma service in separate department	Mixed adult-paediatric<br>9500 children/year<br>Minor trauma service	Paediatric only<br>27,000 children/year<br>Major trauma service	Paediatric only<br>60,000 children/year<br>Major trauma service	Paediatric only<br>22,000 children/year<br>Major trauma service in separate department
Inclusion period	01-01-2012 to 31-12-2014	01-05-2014 to 01-11-2015	01-07-2014 to 28-02-2015	01-03-2014 to 28-02-2015	01-01-2014 to 31-12-2014
Study population	n = 18,594 (16%)	n = 10,584 (9%)	n = 15,556 (13%)	n = 53,175 (45%)	n = 20,300 (18%)

For peer review only - http://bmjopen.bmj.com/site/about/guidelines.xhtml

APPENDIX 3. Distinction between medical and trauma/injury chief complaints based on presentational flowcharts of the Manchester Triage System

Category	Manchester Triage System flowchart
Medical	Abdominal pain in adults, Abdominal pain in children, Abscesses and local infections, Allergy, Apparently drunk, Asthma, Behaving strangely, Chest pain, Collapsed adult, Crying baby, Dental problems, Diabetes, Diarrhoea and vomiting, Ear problems, Eye problems, Fits, Foreign body, GI bleeding, Headache, Irritable child, Limping child, Mental illness, Overdose and poisoning, Palpitations, Pregnancy, PV bleeding, Rashes, Self-harm, Sexually acquired infection, Shortness of breath in adults, Shortness of breath in children, Sore throat, Testicular pain, Unwell adult, Unwell child, Urinary problems, Worried parent, General/Other
Trauma or injury	Assault, Back pain, Bites and stings, Burns and scalds, Exposure to chemicals, Facial problems, Falls, Head injury, Limb problems, Major trauma, Neck pain, Torso injury, Wounds and Major incidents

For peer review only

For peer review only - http://bmjopen.bmj.com/site/about/guidelines.xhtml

APPENDIX 4. Baseline characteristics of the study population for each of the participating hospitals

Table 4.1 Baseline characteristics of children presenting with medical problems

	Erasmus MC	Maasstad Hospital	St Mary's Hospital	Hospital Fernando da Fonseca	General Hospital, Vienna
Sex					
Male	6,871 (57%)	2,860 (57%)	6,323 (55%)	22,146 (51%)	9,979 (53%)
Female	5,111 (43%)	2,182 (43%)	5,238 (45%)	21,072 (49%)	8,956 (47%)
Age					
0 – < 1 yrs	3,210 (27%)	1,471 (29%)	2,528 (22%)	6,830 (16%)	3,210 (17%)
1 – < 12 yrs	7,226 (60%)	2,911 (58%)	7,894 (68%)	31,872 (74%)	13,512 (71%)
≥ 12 yrs	1,546 (13%)	660 (13%)	1,139 (10%)	4,516 (10%)	2,213 (12%)
MTS urgency					
Emergent | Very urgent	2044 (17%)	1217 (24%)	1359 (12%)	5852 (12%)	966 (5%)
Urgent	5717 (48%)	2786 (55%)	3239 (28%)	8352 (28%)	3583 (19%)
Standard | Non-urgent	4,221 (35%)	1,039 (21%)	6,963 (60%)	29,014 (67%)	14,386 (76%)
Diagnostics					
Laboratory	5,716 (48%)	1,932 (38%)	1,370 (12%)	6,671 (15%)	7,190 (38%)
Imaging	2,116 (18%)	623 (12%)	856 (7%)	6,541 (15%)	1,439 (8%)
Medication					
Inhalation	751 (6%)	612 (12%)	1,060 (9%)	5,207 (12%)	901 (5%)
Intravenous	1,712 (14%)	1,058 (21%)	594 (5%)	3,703 (9%)	851 (5%)
Disposition, n (%)					
ICU / mortality at ED	437 (4%)	16 (0.3%)	22 (0.2%)	130 (0.3%)	5 (< 0.1%)
Hospital admission	3,020 (25%)	1,870 (37%)	1,389 (12%)	2,390 (6%)	1,038 (6%)
Discharge / other	8,525 (71%)	3,156 (63%)	10,150 (88%)	40,698 (94%)	17,892 (95%)

For peer review only

For peer review only - http://bmjopen.bmj.com/site/about/guidelines.xhtml

Table 4.2 Baseline characteristics of children presenting with trauma or injuries

	Erasmus MC	Maasstad Hospital	St Mary's Hospital	Hospital Fernando da Fonseca
Sex				
Male	3,566 (59%)	3,078 (57%)	2,354 (59%)	5,539 (56%)
Female	2,476 (41%)	2,362 (43%)	1,641 (41%)	4,418 (44%)
Age				
0 – < 1 yrs	347 (6%)	152 (3%)	24 (6%)	260 (3%)
1 – < 12 yrs	4,286 (71%)	3,574 (66%)	2,848 (71%)	6,575 (66%)
≥ 12 yrs	1,409 (23%)	1,714 (32%)	902 (23%)	3,122 (31%)
MTS urgency				
Emergent | Very urgent	383 (6%)	298 (5%)	246 (6%)	370 (4%)
Urgent	3028 (50%)	2324 (43%)	722 (18%)	2599 (26%)
Standard | Non-urgent	2,631 (44%)	2,818 (52%)	3,027 (76%)	6,988 (70%)
Diagnostics				
Laboratory	423 (7%)	166 (3%)	188 (5%)	319 (3%)
Imaging	2,267 (38%)	3,275 (60%)	1,400 (35%)	6,083 (61%)
Medication				
Intravenous	413 (7%)	285 (5%)	109 (3%)	306 (3%)
Disposition, n (%)				
ICU / mortality at ED	63 (1%)	1 (< 0.1%)	4 (0.1%)	6 (0.1%)
Hospital admission	68 (11%)	563 (10%)	210 (5%)	222 (2%)
Discharge / other	5,295 (88%)	4,876 (90%)	3,781 (95%)	9729 (98%)

For peer review only

For peer review only - http://bmjopen.bmj.com/site/about/guidelines.xhtml

APPENDIX 5. Gender differences per age category

Figure 5.1 Gender differences per age category in children presenting with general medical problems

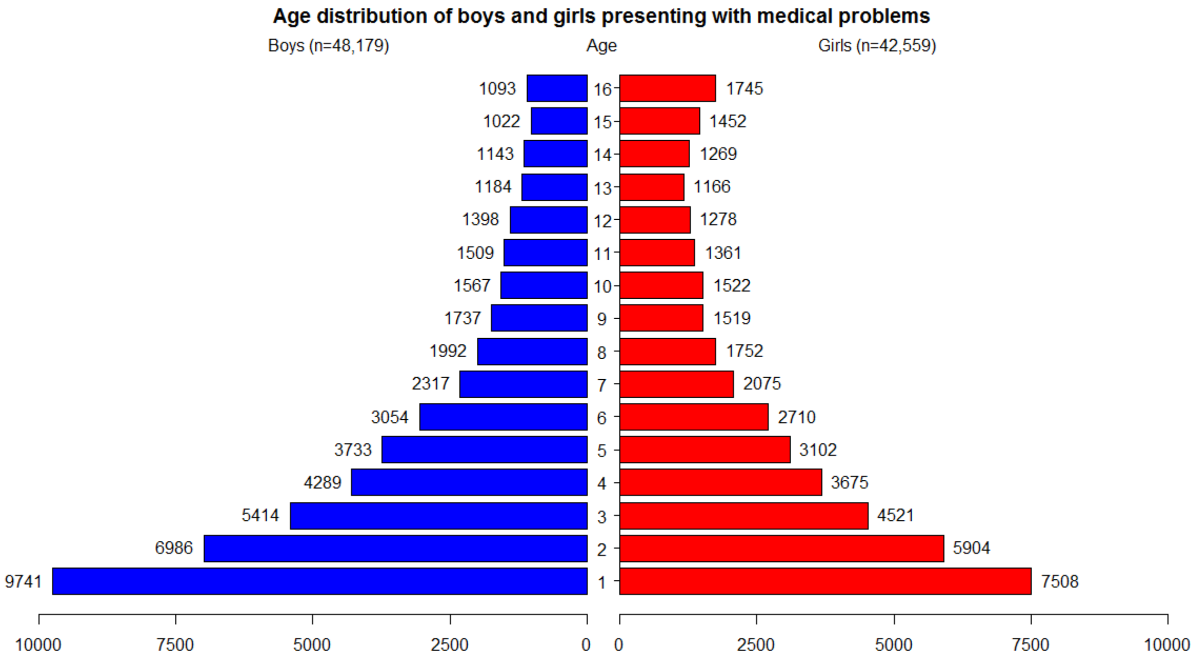

eview only

Figure 5.2 Gender differences per age category in children presenting with trauma or injuries

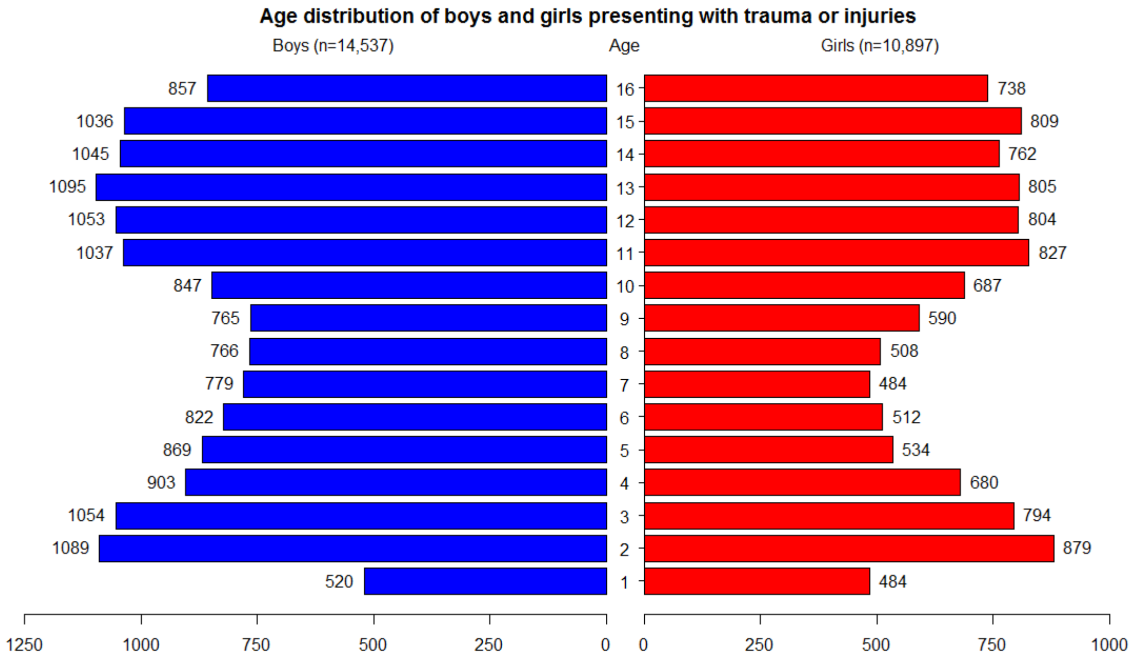

For peer review only - http://bmjopen.bmj.com/site/about/guidelines.xhtml

APPENDIX 6. Gender differences in laboratory tests and inhalation medication at the ED in subgroups of patients based type of presenting problem

		Laboratory tests				Inhalation medication			
			Heterogeneity				Heterogeneity		
		Pooled OR (95%CI) [a]	I^2	Q	p	*Pooled OR (95%CI)* [a, b]	I^2	Q	p
Total population	Female	1.12 (1.08-1.18)	41%	6.2	0.19	0.76 (0.70-0.83)	49%	8.0	0.09
	Male	Reference				Reference			
		Pooled OR (95%CI) [a]	I^2	Q	p	*Pooled OR (95%CI)* [a]	I^2	Q	p
Fever	Female	1.11 (0.98-1.27)	45%	7.2	0.13	0.90 (0.75-1.08)	0%	3.4	0.50
	Male	Reference				Reference			
Shortness of breath	Female	1.14 (1.04-1.25)	0%	3.0	0.55	0.80 (0.74-0.87)	0%	3.6	0.47
	Male	Reference				Reference			

[a] Summary OR based on a random effects model where results from individual settings are pooled. Model adjusted for age and triage urgency
[b] Summary OR based on a random effects model where results from individual settings are pooled. Model adjusted for triage urgency

For peer review only - http://bmjopen.bmj.com/site/about/guidelines.xhtml

STROBE Statement—Checklist of items that should be included in reports of ***cohort studies***

"SEX-SPECIFIC DIFFERENCES IN CHILDREN ATTENDING THE EMERGENCY DEPARTMENT: PROSPECTIVE OBSERVATIONAL STUDY"*, 29-08-2019*

	Item No	Recommendation	
**Title and abstract**	1	(*a*) Indicate the study's design with a commonly used term in the title or the abstract	*The study design, a prospective observational study, is stated in the title and abstract.*
		(*b*) Provide in the abstract an informative and balanced summary of what was done and what was found	*The abstract is written according to the guidelines with all required information.*
**Introduction**			
Background/rationale	2	Explain the scientific background and rationale for the investigation being reported	*The background and rationale are described in the first two paragraphs of the introduction section.*
Objectives	3	State specific objectives, including any prespecified hypotheses	*The objective is stated in the last sentence of the introduction. There were no prespecified hypothesis.*
**Methods**			
Study design	4	Present key elements of study design early in the paper	*Key elements of the study design are presented in the first paragraph of the methods, and in detail explained in the consequent paragraphs*
Setting	5	Describe the setting, locations, and relevant dates, including periods of recruitment, exposure, follow-up, and data collection	*The setting, location and relevant dates are described in paragraph 'Study settings and patient population', in the methods section. A detailed description of all settings is added in supplemental information 2.*
Participants	6	(*a*) Give the eligibility criteria, and the sources and methods of selection of participants. Describe methods of follow-up	*All children under the age of 16 attending the ED during the study period were eligible, as reported in the paragraph 'Study settings and patient population' in the methods section.*
		(*b*) For matched studies, give matching criteria and number of exposed and unexposed	*Not applicable*
Variables	7	Clearly define all outcomes, exposures, predictors, potential confounders, and effect modifiers. Give diagnostic criteria, if applicable	*The main determinant of the study is sex, defined as male or female. The determinant and all outcomes are described in the 'Determinants' paragraph in the methods section.*
Data sources/ measurement	8*	For each variable of interest, give sources of data and details of methods	*All variables are routinely recorded in patients' electronic health*

For peer review only - http://bmjopen.bmj.com/site/about/guidelines.xhtml

		of assessment (measurement). Describe comparability of assessment methods if there is more than one group	*records, and automatically extracted, as reported in the paragraph 'Data collection' of the methods section.*
Bias	9	Describe any efforts to address potential sources of bias	*ED staff involved in the data collection were not aware of the research question, thereby minimizing information bias The number of patients with missing data was very small (1.5%), thereby limiting bias due to missing data.*
Study size	10	Explain how the study size was arrived at	*Because of the observational nature of the study, we did not perform a formal sample size calculation. As a general rule of thumb, a regression model should have at least 10-15 cases with the outcome for each of the predictors included in the model. As can be seen in table 1, all relevant outcomes occurred in more than 1000 patients, enough to study more than 66 determinants.*
Quantitative variables	11	Explain how quantitative variables were handled in the analyses. If applicable, describe which groupings were chosen and why	*The handling of variables is described in the 'Determinants' paragraph of the methods section.*
Statistical methods	12	(*a*) Describe all statistical methods, including those used to control for confounding	*The statistical methods are described in the 'Data analysis' paragraph of the methods section.*
		(*b*) Describe any methods used to examine subgroups and interactions	*We pre-specified 4 subgroups of medical patients, because we expected to obtain more homogeneous results.*
		(*c*) Explain how missing data were addressed	*Because the number of patients with missing data was very small (1.5%) we used a complete case approach, as described in the paragraph 'Study settings and patient population'.*
		(*d*) If applicable, explain how loss to follow-up was addressed	*Not applicable*
		(*e*) Describe any sensitivity analyses	*Not applicable*
**Results**			
Participants	13*	(a) Report numbers of individuals at each stage of study—eg numbers potentially eligible, examined for eligibility, confirmed eligible, included in the study, completing follow-up, and analysed	*The patient flow is described in the first paragraph of the results section. All tables include the numbers of patients that were analysed.*
		(b) Give reasons for non-participation	*Not applicable*

For peer review only - http://bmjopen.bmj.com/site/about/guidelines.xhtml

		at each stage	
		(c) Consider use of a flow diagram	-
Descriptive data	14*	(a) Give characteristics of study participants (eg demographic, clinical, social) and information on exposures and potential confounders	*Characteristics of the study population are described in table 1 and supplemental information 4.*
		(b) Indicate number of participants with missing data for each variable of interest	*The number of participants with missing data is provided in the 'Study settings and patient population' paragraph of the methods section. Missing information was limited to only two variables: triage urgency and triage flowchart (presenting problem)*
		(c) Summarise follow-up time (eg, average and total amount)	*Not applicable*
Outcome data	15*	Report numbers of outcome events or summary measures over time	*The number of patients with the outcome are reported in table 1.*
Main results	16	(*a*) Give unadjusted estimates and, if applicable, confounder-adjusted estimates and their precision (eg, 95% confidence interval). Make clear which confounders were adjusted for and why they were included	*Unadjusted estimates, i.e. absolute numbers are presented in table 1. Adjusted estimates and 95% confidence intervals are presented in figures 1 and 2. Analyses were adjusted for age and triage urgency as mentioned in the "Data analysis" paragraph.*
		(*b*) Report category boundaries when continuous variables were categorized	*The only continuous variable, age, was maintained continues, as described in the "Determinants" paragraph of the methods section.*
		(*c*) If relevant, consider translating estimates of relative risk into absolute risk for a meaningful time period	*Not applicable*
Other analyses	17	Report other analyses done—eg analyses of subgroups and interactions, and sensitivity analyses	*Pre-specified subgroup analyses were performed and described in the "Data analyses" paragraph of the methods section, in the results section and in supplemental information 6.*
**Discussion**			
Key results	18	Summarise key results with reference to study objectives	*Key results are stated in the first paragraph and discussed in the subsequent paragraphs of the discussion section.*
Limitations	19	Discuss limitations of the study, taking into account sources of potential bias or imprecision. Discuss both direction and magnitude of any potential bias	*Limitations of the study are addressed in the 6h paragraph of the discussion, starting with "These results should be interpreted in light of the limitations of…"*

For peer review only - http://bmjopen.bmj.com/site/about/guidelines.xhtml

Interpretation	20	Give a cautious overall interpretation of results considering objectives, limitations, multiplicity of analyses, results from similar studies, and other relevant evidence	*The interpretation of the study is discussed in the 2nd to 4th paragraphs of the discussion.*
Generalisability	21	Discuss the generalisability (external validity) of the study results	*Generalisability of the study is discussed in the 5th paragraph of the discussion, in the section discussing the strengths of the study.*
**Other information**			
Funding	22	Give the source of funding and the role of the funders for the present study and, if applicable, for the original study on which the present article is based	*The source and role of funding is provided on the title page.*

*Give information separately for exposed and unexposed groups.

Note: An Explanation and Elaboration article discusses each checklist item and gives methodological background and published examples of transparent reporting. The STROBE checklist is best used in conjunction with this article (freely available on the Web sites of PLoS Medicine at http://www.plosmedicine.org/, Annals of Internal Medicine at http://www.annals.org/, and Epidemiology at http://www.epidem.com/). Information on the STROBE Initiative is available at http://www.strobe-statement.org.

For peer review only

For peer review only - http://bmjopen.bmj.com/site/about/guidelines.xhtml

BMJ Open

Sex-Specific Differences in Children Attending the Emergency Department: Prospective Observational Study

Journal:	*BMJ Open*
Manuscript ID	bmjopen-2019-035918.R1
Article Type:	Original research
Date Submitted by the Author:	19-Mar-2020
Complete List of Authors:	Zachariasse, Joany; Erasmus MC- Sophia Children's Hospital, Department of General Paediatrics<br>Borensztajn, Dorine; Erasmus MC Sophia Children Hospital, General Paediatrics<br>Nieboer, Daan; Erasmus MC - University Medical Center Rotterdam, Public Health<br>Alves, Claudio; Hospital Fernando Fonseca, General paediatrics, Emergency Unit<br>Greber-Platzer, Susanne; Medical University of Vienna, Department of Pediatrics and Adolescent Medicine<br>Keyzer-Dekker, Claudia; Erasmus MC- Sophia Children's Hospital, Department of Pediatric Surgery<br>Maconochie, Ian; Department of Paediatric Emergency,<br>Steyerberg, Ewout; Leiden University Medical Center, Department of Medical Statistics<br>Smit, Frank; Maasstad Ziekenhuis, Department of paediatrics<br>moll, Henriette; Erasmus University Medical Center Sophia Children�s hospital, Paediatrics
**Primary Subject Heading**:	Paediatrics
Secondary Subject Heading:	Emergency medicine, Epidemiology
Keywords:	EPIDEMIOLOGY, Paediatric A&E and ambulatory care < PAEDIATRICS, PAEDIATRICS

SCHOLARONE™
Manuscripts

I, the Submitting Author has the right to grant and does grant on behalf of all authors of the Work (as defined in the below author licence), an exclusive licence and/or a non-exclusive licence for contributions from authors who are: i) UK Crown employees; ii) where BMJ has agreed a CC-BY licence shall apply, and/or iii) in accordance with the terms applicable for US Federal Government officers or employees acting as part of their official duties; on a worldwide, perpetual, irrevocable, royalty-free basis to BMJ Publishing Group Ltd ("BMJ") its licensees and where the relevant Journal is co-owned by BMJ to the co-owners of the Journal, to publish the Work in this journal and any other BMJ products and to exploit all rights, as set out in our licence.

The Submitting Author accepts and understands that any supply made under these terms is made by BMJ to the Submitting Author unless you are acting as an employee on behalf of your employer or a postgraduate student of an affiliated institution which is paying any applicable article publishing charge ("APC") for Open Access articles. Where the Submitting Author wishes to make the Work available on an Open Access basis (and intends to pay the relevant APC), the terms of reuse of such Open Access shall be governed by a Creative Commons licence – details of these licences and which Creative Commons licence will apply to this Work are set out in our licence referred to above.

Other than as permitted in any relevant BMJ Author's Self Archiving Policies, I confirm this Work has not been accepted for publication elsewhere, is not being considered for publication elsewhere and does not duplicate material already published. I confirm all authors consent to publication of this Work and authorise the granting of this licence.

For peer review only

Sex-Specific Differences in Children Attending the Emergency Department: Prospective Observational Study

Joany M Zachariasse, Dorine M Borensztajn, Daan Nieboer, Claudio F Alves, Susanne Greber-Platzer, Claudia M G Keyzer – Dekker, Ian K Maconochie, Ewout W Steyerberg, Frank J Smit, Henriëtte A Moll

Author affiliations:

Department of General Paediatrics, Erasmus MC- Sophia Children's Hospital, University Medical Centre Rotterdam, PO Box 2060, 3000 CB Rotterdam, The Netherlands

Joany M Zachariasse

PhD-student

Department of General Paediatrics, Erasmus MC- Sophia Children's Hospital, University Medical Centre Rotterdam, PO Box 2060, 3000 CB Rotterdam, The Netherlands

Dorine M Borensztajn

Paediatrician

Department of Public Health, Erasmus MC-University Medical Center Rotterdam, P.O. Box 2040

3000 CA Rotterdam The Netherlands

Daan Nieboer

PhD-student

Department of Paediatrics, Emergency Unit, Hospital Professor Doutor Fernando da Fonseca, EPE, IC 19, 2720-276 Amadora, Lisbon, Portugal

Claudio F Alves

Paediatrician

For peer review only - http://bmjopen.bmj.com/site/about/guidelines.xhtml

Medical University Vienna, Department of Pediatrics and Adolescent Medicine, Währinger Gürtel 16, 1090, Vienna, Austria

Susanne Greber-Platzer

Professor

Department of Pediatric Surgery, Erasmus MC-University Medical Center Rotterdam, PO Box 2060, 3000 CB Rotterdam, The Netherlands

Claudia M G Keyzer - Dekker

Paediatric Surgeon

Department of Paediatric Emergency Medicine, Imperial College NHS Healthcare Trust, London, United Kingdom

Ian K Maconochie

Associate professor

Department of Medical Statistics and Bioinformatics, Leiden University Medical Center, the Netherlands

and

Department of Public Health, Erasmus MC-University Medical Center Rotterdam, P.O. Box 2040, 3000 CA Rotterdamm The Netherlands

Ewout Steyerberg

Professor

Department of Paediatrics, Maasstad Hospital, P.O. Box 9100, 3007 AC Rotterdam, The Netherlands

Frank J Smit

Paediatrician

For peer review only

For peer review only - http://bmjopen.bmj.com/site/about/guidelines.xhtml

Department of General Paediatrics, Erasmus MC- Sophia Children's Hospital, University Medical Centre Rotterdam, PO Box 2060, 3000 CB Rotterdam, The Netherlands

Henriëtte A Moll

Professor

Corresponding author:

Prof. Dr. Henriëtte A. Moll

h.a.moll@erasmusmc.nl

Keywords [MeSH]: sex, emergency medicine, child, epidemiology, prospective studies

Word count: 2554

Word count abstract: 297

Abbreviations:

CI - confidence interval

ED – emergency department

IOM – Institute of Medicine

MTS – Manchester Triage System

OR – odds ratio

Funding: This research received no specific grant from any funding agency in the public, commercial or not-for-profit sectors.

Ethical approval: The research protocol was reviewed and approved by the local medical ethics committees and requirement for informed consent was waived.

Competing interests: The authors declare they have no potential conflicts of interest to disclose.

For peer review only - http://bmjopen.bmj.com/site/about/guidelines.xhtml

Contributorship statement: JZ contributed to the conceptualization and design of the study, carried out the analyses, and drafted the initial manuscript. DB, FS, CA, CK, IM, and SG contributed to the conceptualization and design of the study, coordinated the data collection, contributed to interpretation of the data, and critically reviewed the manuscript. DN and ES contributed to the conceptualization and design of the study, supervised the analyses, and critically reviewed the manuscript. HM contributed to the conceptualization and design of the study, contributed to interpretation of the data, critically reviewed the manuscript and supervised the study. All authors approved the final manuscript as submitted and agree to be accountable for all aspects of the work.

Transparency declaration: The lead author affirms that this manuscript is an honest, accurate, and transparent account of the study being reported; that no important aspects of the study have been omitted; and that any discrepancies from the study as planned have been explained.

Copyright licence: The Corresponding Author has the right to grant on behalf of all authors and does grant on behalf of all authors, a worldwide licence to the Publishers and its licensees in perpetuity, in all forms, formats and media (whether known now or created in the future), to i) publish, reproduce, distribute, display and store the Contribution, ii) translate the Contribution into other languages, create adaptations, reprints, include within collections and create summaries, extract and/or, abstracts of the Contributions, iii) create any other derivative work(s) based on the Contribution, iv) to exploit all subsidiary rights in the Contribution, v) the inclusion of electronic links from the Contribution to third party material where-ever it may be located; and vi) licence any third party to do any or all of the above.

Dating sharing: Data from this study are available upon request to the corresponding author of the study (h.a.moll@erasmusmc.nl), subject to local rules and regulations.

Acknowledgements: We gratefully acknowledge Rikke Jorgensen, paediatric research nurse (St. Mary's Hospital, London) and Pinky Rose Espina, paediatric resident (General Hospital, Vienna) for their assistance with data collection for this study.

For peer review only - http://bmjopen.bmj.com/site/about/guidelines.xhtml

Abstract

Objective To assess the role of sex in the presentation and management of children attending the emergency department (ED).

Design The TrIAGE project, a prospective observational study based on curated electronic health record data

Setting Five diverse European hospitals in four countries (Austria, Netherlands, Portugal, United Kingdom)

Participants All consecutive paediatric ED visits of children under the age of 16 during the study period (8 to 36 months between 2012-2015)

Main outcome measures The association between sex (boy or girl) and diagnostic tests and disease management in general paediatric ED visits and in subgroups presenting with trauma or musculoskeletal, gastrointestinal, and respiratory problems and fever. Results from the different hospitals were pooled in a random effects meta-analysis.

Results 116,172 ED visits were included of which 63,042 (54%) by boys and 53,715 (46%) by girls. Boys accounted for the majority of ED visits in childhood, and girls in adolescence. After adjusting for age, triage urgency and clinical presentation, girls had more laboratory tests compared with boys (pooled OR 1.10, 95%CI 1.05-1.15). Additionally, girls had more laboratory tests in ED visits for respiratory problems (pooled OR 1.15, 95%CI 1.04-1.26) and more imaging in visits for trauma or musculoskeletal problems (pooled OR 1.10, 95%CI 1.01-1.20) and respiratory conditions (pooled OR 1.14, 95%CI 1.05-1.24). Girls with respiratory problems were less often treated with inhalation medication (pooled OR 0.76, 95%CI 0.70-0.83). There was no difference in hospital admission between the sexes (pooled OR 0.99, 95%CI 0.95-1.04).

Conclusion In childhood, boys represent the majority of ED visits and they receive more inhalation medication. Unexpectedly, girls receive more diagnostic tests compared with boys . Further research is needed to investigate whether this is due to pathophysiological differences and differences in disease course, whether girls present signs and symptoms differently, or whether sociocultural factors are responsible.

For peer review only

For peer review only - http://bmjopen.bmj.com/site/about/guidelines.xhtml

Strengths and limitations of the study

- The present study was based on a large, multicentre and international cohort of more than 100,000 emergency department visits

- The study is the first to assess the role of sex in the prevalence and type of clinical presentations and in the diagnostics and management of children attending the emergency department

- Due to the use of standardized, routinely collected data, only general patterns and broad subgroups of patients could be evaluated

- Our cohort of ED visits included repeat visits from the same child, which could have led to underestimation of the standard errors and potentially to wider confidence intervals

- Only two hospitals in our study included patients with major trauma, which limits generalizability to this subgroup of patients

For peer review only

For peer review only - http://bmjopen.bmj.com/site/about/guidelines.xhtml

Introduction

There is increasing evidence that differences between men and women are important in the epidemiology, pathophysiology, treatment and outcome of many diseases.[1-3] The Institute of Medicine (IOM) has emphasized that sex is an important variable that should be considered in biomedical and health related research.[4] Sex refers to the biological differences between men and women, while gender refers to a broader concept including social and cultural distinctions associated with a given sex.[3] In the area of emergency medicine, sex and gender-specific differences have been found in adults, in topics as broad as the clinical presentation and outcomes of acute myocardial infarction[5], the prevalence and survival of out-of-hospital cardiac arrest[6] and the epidemiology of sports-related injuries.[7]

In children, research on the impact of sex and gender on health is scarce, particularly in the field of emergency medicine. Only twenty-two relevant articles described sex or gender differences in children related to emergency medicine between January 2000 and November 2017, according to PubMed (Appendix 1). Of these, only two studies described differences between sexes in overall emergency department resource use or management. Both studies observed higher ED attendance rates in boys compared with girls, but did not address specific types of clinical presentations or evaluate differences in disease management or outcome.[8 9] Other studies focused on specific disease groups such as trauma and injuries, mental health related conditions, and asthma. In these areas studies report conflicting results regarding differences in the rate of ED visits between boys and girls. Diagnosis and management were poorly studied. Only one study in children with asthma looked at differences in ED management and did not find a difference in treatment between boys and girls. [10]

The aim of the current study is to assess the role of sex in the clinical presentation, diagnostics and management in the general population of children attending the emergency department (ED). Through this study, we aim to gain more insight in sex-specific differences in paediatric emergency medicine and identify areas for future research.

For peer review only - http://bmjopen.bmj.com/site/about/guidelines.xhtml

Methods

This study was embedded in the TrIAGE project, a prospective observational study, based on electronic health record data, in five EDs in four European countries (the Netherlands, United Kingdom, Austria, Portugal). The study was approved by the medical ethical committees of the participating institutions: Medical Ethics Committee Erasmus MC (MEC-2013-567), Board of Directors Maasstad Ziekenhuis (L2013-103), Imperial College London Joint Research Compliance Office (14/WA/1051), Comissão de Ética para a Saúde do Hospital Prof. Dr. Fernando Fonseca EPE (Estudo Clínico TrIAGE – Parecer Favorável), Ethik Kommission Medizinische der Medizinischen Unversität Wien (EK Nr: 1405/2014). All waived the requirement for informed consent.

Study settings and patient population

In the TrIAGE study, 119,209 consecutive ED visits of children and adolescents under the age of 16 years were included. Enrolment took place during a period of 8 to 36 months between 2012 and 2015 in five diverse study sites (Appendix 2). We restricted our analysis to ED visits with complete triage data, thereby excluding all visits with missing triage categories or missing presenting problem. The General Hospital Vienna only included ED visits for medical complaints because the majority of trauma patients were seen in the department of traumatology. A small proportion of low urgent trauma cases were still seen in the ED by the “in-house paediatrician” and we excluded these remaining patients to reduce selection bias.

Data collection

The TrIAGE project is based on routinely collected, standardized electronic health record data. At the start of the study, a set of minimally required variables was determined. Completeness and accuracy of the data was discussed during a site visit and telephone meetings using a checklist quality control. With posters, newsletters and presentations, nurses were encouraged to complete the full medical records including vital sign measurements.

During the study period, nurses and physicians entered the clinical data in the medical or nursing records. After data extraction, careful data harmonization and quality checks were performed.

For peer review only - http://bmjopen.bmj.com/site/about/guidelines.xhtml

Determinants

In all participating hospitals, sex (boy or girl) and age were routinely registered for each patient as part of the administrative process.

We used triage data to determine type of presenting problem and triage urgency. All participating hospitals used the Manchester Triage System (MTS). This flowchart-based emergency medical triage system is the most commonly used triage system in Europe. In the MTS, the triage nurse is required to select a flowchart for each patient, representing the chief complaint, such as *Shortness of Breath* or *Wounds*. Each flowchart consists of signs and symptoms named discriminators that are ranked by priority. The nurse then gathers information on the discriminators from top to bottom. Selection of a discriminator allocates the patient to the related urgency category ranging from “Immediate” (0 minutes maximum waiting time) to “Non-urgent” (240 minutes maximum waiting time).[11]

Age, triage urgency and clinical presentation were considered as potential confounding variables in the relationship between sex and disease management. Age was maintained as a continuous variable. Triage urgency was defined as the MTS urgency category and placed into three groups: high urgent (MTS category immediate or high urgent), urgent (MTS category urgent) and low urgent (MTS category standard or non-urgent). Three hospitals (Erasmus MC, General Hospital Vienna and Hospital Fernando da Fonseca) implemented MTS modifications. To ensure consistency among hospitals, the urgency levels according to MTS version 3 with Dutch modifications for children with fever were modelled in all hospitals.[11 12] We distinguished ten clinical presentations, based on the main problem presented during triage according to MTS flowchart (Appendix 3).

Data analysis

In descriptive analyses, differences between boys and girls in the type and severity of problems with which they presented at the ED were explored.

Multivariable logistic regression models were used to assess the association between sex and diagnostics and management in the ED for each study site. We selected laboratory tests and imaging as important markers for diagnostics, and inhalation medication, intravenous medication or fluids and

For peer review only - http://bmjopen.bmj.com/site/about/guidelines.xhtml

hospital admission as markers for disease management. Analyses were adjusted for age, triage urgency and clinical presentation, and boys were determined as the reference group. The results of the individual hospitals were pooled in a random effects meta-analysis and presented in forest plots. ORs were considered statistically significant when the 95% confidence interval (CI) did not include 1. Heterogeneity among settings was assessed using the Cochran's Q statistic and the I^2 statistic.

We performed subgroup analyses in subgroups of children presenting with trauma or musculoskeletal problems, children presenting with fever, children presenting with gastro-intestinal problems and children presenting with shortness of breath. Fever was defined as either a temperature of ≥ 38.5°C at the ED or the item "fever" selected as triage discriminator. Shortness of breath, gastrointestinal and trauma or musculoskeletal problems were defined based on the triage flowchart. We selected these four clinical presentations because they were among the largest subgroups of patients and represent important clinical entities in children.

Statistical analyses were performed in SPSS Statistics version 21.0 (IBM Corp, Armonk, NY, USA) and R version 3.4.2 (R Foundation, Vienna, Austria). The package metafor was used to conduct the random effects meta-analysis and create forest plots.

Patient and public involvement

There was no patient involvement in the design of the study or the writing of the manuscript. We plan to disseminate the results to patient organisations, clinicians and policy makers working in emergency care upon publication.

For peer review only - http://bmjopen.bmj.com/site/about/guidelines.xhtml

Results

Of all 119,209 ED visits included in the TrIAGE cohort, 116,757 ED visits (98%) were included in the study (Figure 1). There was no significant difference between the sexes in the proportion of excluded ED visits (Pearson's Chi-Square(1) = 0.08, p-value 0.77. General characteristics of the study population are presented in Table 1.

The relation between sex and age, triage urgency and clinical presentation

In the total study population, the sex imbalance in number of ED visits changed with age. In childhood, consistently more boys than girls attended the ED, and the ratio reversed in adolescence (Table 2). Boys accounted for the majority of visits in all triage urgency levels, most evidently in the high urgency categories. They had the highest proportion of ED visits with dermatological, respiratory and traumatic complaints, while in children presenting with cardiac, ENT, gastrointestinal and neurologic conditions, the boy to girl ratio was almost equal.

We further explored the relation between sex and triage urgency. The higher rate of boys' ED visits in childhood and girls' ED visits in adolescence was consistent among the different urgency categories. Also, the sex-specific differences in the different types of clinical presentation were similar in the high, intermediate and low urgency categories. An exception was the subgroup of uro- and gynaecological presentations where there were almost only boys prioritized as high urgency. Also notable was the subgroup of respiratory conditions that represents the largest population of high urgency visits with a 1.5 times higher proportion of boys compared to girls (Appendix 4).

Sex-specific differences in diagnostics at the ED

In the general population of children visiting the ED, adjusted for age, triage urgency and clinical presentation, girls had more laboratory tests at the ED than boys (pooled OR 1.10, 95%CI 1.05-1.15) (Table 3, Figure 2). This finding was consistent in all hospitals, although there was some evidence for heterogeneity (Q-statistic 7.27, p=0.12, I^2 statistic 45.8%). Significant higher rates of laboratory testing in girls were also seen in the subgroup of ED visits for respiratory problems (pooled OR 1.15, 95%CI 1.04-

For peer review only - http://bmjopen.bmj.com/site/about/guidelines.xhtml

1.26) and trends were observed in ED visits for trauma or musculoskeletal problems (pooled OR 1.12, 95%CI 0.94-1.35) and fever (pooled OR 1.12, 95%CI 0.98-1.27) (Appendix 5).

In the overall population, no differences in imaging between the sexes were found. However, in the subgroups of ED visits for trauma or musculoskeletal problems and respiratory conditions, more imaging was conducted in girls (pooled OR 1.10, 95%CI 1.01-1.20 and pooled OR 1.14, 95%CI 1.05-1.24 respectively).

Sex-specific differences in management at the ED

In the general population of children attending the ED, adjusted for age, triage urgency and clinical presentation, there was no difference in the rate of IV medication or fluids (OR 0.96, 95%CI 0.89-1.03) and in the rate of hospital admission (OR 0.99, 95%CI 0.95-1.04) (Table 3, Figure 2). Neither was this difference seen in one of the subgroups. Remarkably, there was a large difference in the use of inhalation medication in the subgroup of ED visits for respiratory conditions. In this subgroup, girls received less inhalation medication (pooled OR 0.76, 95%CI 0.70-0.83) (Appendix 5).

For peer review only

For peer review only - http://bmjopen.bmj.com/site/about/guidelines.xhtml

Discussion

In a European cohort of more than 100,000 ED visits, boys accounted for the majority of ED visits in childhood, while in adolescence, the boy to girl ratio reversed. We found evidence that more diagnostic tests were conducted in girls compared with boys: girls had more laboratory tests overall (pooled OR 1.10, 95%CI 1.05-1.15), and in the subgroup of ED visits for respiratory problems (pooled OR 1.15, 95%CI 1.04-1.26) while girls received more imaging in the ED visits for trauma or musculoskeletal problems (pooled OR 1.10, 95%CI 1.01-1.20) and for respiratory conditions (pooled OR 1.14, 95%CI 1.05-1.24). Girls, however, received less inhalation medication when presenting with respiratory problems (pooled OR 0.76, 95%CI 0.70-0.83). There was no difference between sexes in the odds of hospital admission (OR 0.99, 95%CI 0.95-1.04).

This is the first study that assesses the role of sex in paediatric emergency medicine, using a large, multicentre and international cohort. Despite the diversity in study sites, results were comparable across the participating EDs, supporting generalizability of the findings.

The higher attendance rate of boys in European EDs is consistent with findings from the US National Hospital Ambulatory Medical Surveys.[13] Diverse biological explanations have been proposed, such as an increased susceptibility to infections and higher risk of lung disease in men, due to male sex hormones.[14-16] Behavioural, social and cultural factors might lead to differences in the number and types of ED visits as well. These may include differences in activities, risk taking behaviour, parenting practices, and different referral decisions by primary care providers.[17 18] Sex hormones have been considered responsible for differences in lung development and differences in prevalence and severity of lung diseases.[14 15 19] A higher prevalence of asthma in boys during childhood has previously been reported, although reports in emergency care have been inconsistent.[16 20] However, boys' increased susceptibility to respiratory conditions could indicate a more severe disease course, possibly explaining the higher rate of inhalation medications used in our study.

Beside sex differences in disease presentations, our study found differences in diagnostics between boys and girls. We did not find any previous publication with a similar finding. Sex-specific differences in the need for diagnostic tests could be caused by differences in disease severity, but may also be attributed to differences in clinical presentation. Boys and girls may differ regarding the presenting signs and symptoms, the way they express pain or distress, or their ability to phrase their symptoms. Diagnostic uncertainty due to a-specific disease presentation may warrant more diagnostic tests. Finally, it is

For peer review only - http://bmjopen.bmj.com/site/about/guidelines.xhtml

possible that provider attitude subconsciously differs in boys compared with girls. In adults, implicit gender bias in diagnostics and treatment has been found in observational research and standardized case scenarios. [21 22] Our analyses are exploratory and our findings require further research. Future studies are needed that address sex-specific differences in the conducting of diagnostic tests. These studies should aim to elucidate whether differences in the need for diagnostics are due to pathophysiological mechanisms and differences in disease course, whether girls present signs and symptoms differently compared with boys, or whether there are other sociocultural factors responsible.

Limitations

These results should be interpreted in light of the limitations of using standardized routinely collected data. Detailed information such as specific types of diagnoses or results from diagnostic tests was not available. Therefore, only general patterns and broad subgroups of patients were evaluated. Furthermore, it is possible that observed differences in diagnostics and management between boys and girls are confounded by sex-specific differences in type and severity of presenting conditions. The analyses were adjusted for age, triage urgency and clinical presentation, and the findings were analysed in specific subtypes of clinical presentations. There may, however, be remaining variation not captured in urgency and presenting problem. Additionally, our cohort of ED visits included repeat visits from the same child which are not independent. In general, ignoring correlated data does not influence effect estimates such as odds ratios, but it can lead bias in the standard errors.[23] By not taking into account the correlation of visits from the same subject we are likely to underestimate the standard errors of our effect sizes, potentially leading to wider confidence intervals. Finally, only two hospitals in our study included patients with major trauma which limits generalizability to this subgroup of patients.

Conclusion

In children, the role of sex and gender on health is largely unknown and research assessing sex -specific differences is scare. Our study found that in childhood boys more often present to the ED compared with girls, while in adolescence this ratio is reversed. The higher need for inhalation medication in boys may represent a higher susceptibility for or a more severe course of respiratory infections. Unexpectedly, girls receive more diagnostic tests compared with boys. Future studies should focus on the role of sex and gender in specific conditions and determine whether there are pathophysiological differences in disease course and severity, whether girls present signs and symptoms differently or whether there are social and cultural factors responsible.

For peer review only

References

1. Doyal L. Sex, gender, and health: the need for a new approach. *Bmj* 2001;323(7320):1061-3.
2. Greenberg MR, Safdar B, Choo EK, et al. Future directions in sex- and Gender-specific Emergency Medicine. *Acad Emerg Med* 2014;21(12):1339-42.
3. Institute of Medicine Committee on Women's Health Research. Women's Health Research: Progress, Pitfalls and Promise. 2010
4. Institute of Medicine Committee on Understanding the Biology of Sex and Gender Differences. Exploring the Biological Contributions to Human Health: Does Sex Matter? 2001
5. Mehta LS, Beckie TM, DeVon HA, et al. Acute Myocardial Infarction in Women: A Scientific Statement From the American Heart Association. *Circulation* 2016;133(9):916-47.
6. Wigginton JG, Perman SM, Barr GC, et al. Sex- and gender-specific research priorities in cardiovascular resuscitation: proceedings from the 2014 Academic Emergency Medicine Consensus Conference Cardiovascular Resuscitation Research Workgroup. *Acad Emerg Med* 2014;21(12):1343-9.
7. Raukar NP, Zonfrillo MR, Kane K, et al. Gender- and sex-specific sports-related injury research in emergency medicine: a consensus on future research direction and focused application. *Acad Emerg Med* 2014;21(12):1370-9.
8. Henneman PL, Nathanson BH, Ribeiro K, et al. The impact of age and gender on resource utilization and profitability in ED patients seen and released. *Am J Emerg Med* 2014;32(10):1159-67.
9. Siminski P, Bezzina AJ, Lago LP, et al. Primary care presentations at emergency departments: rates and reasons by age and sex. *Aust Health Rev* 2008;32(4):700-9.
10. Schatz M, Clark S, Emond JA, et al. Sex differences among children 2-13 years of age presenting at the emergency department with acute asthma. *Pediatr Pulmonol* 2004;37(6):523-9.
11. Mackway-Jones K, Marsden J, Windle J. Emergency Triage: John Wiley & Sons, Ltd. 2014.
12. van Veen M, Steyerberg EW, Van't Klooster M, et al. The Manchester triage system: improvements for paediatric emergency care. *Emerg Med J* 2012;29(8):654-9.
13. Rui P, Okeyode T. National Ambulatory Medical Care Survey: 2015 State and National Summary tables. Available from: http://www.cdc.gov/nchs/ahcd/ahcd/products.htm. Accessed 11-06-2017.
14. Klein SL. The effects of hormones on sex differences in infection: from genes to behavior. *Neurosci Biobehav Rev* 2000;24(6):627-38.
15. Wichmann MW, Inthorn D, Andress HJ, et al. Incidence and mortality of severe sepsis in surgical intensive care patients: the influence of patient gender on disease process and outcome. *Intensive Care Med* 2000;26(2):167-72.
16. Carey MA, Card JW, Voltz JW, et al. It's all about sex: gender, lung development and lung disease. *Trends Endocrinol Metab* 2007;18(8):308-13.
17. Hartzog TH, Timerding BL, Alson RL. Pediatric trauma: enabling factors, social situations, and outcome. *Acad Emerg Med* 1996;3(3):213-20.
18. Greenes DS, Wigotsky M, Schutzman SA. Gender differences in rates of unintentional head injury in the first 3 months of life. *Ambul Pediatr* 2001;1(3):178-80.
19. Martin GS, Mannino DM, Eaton S, et al. The epidemiology of sepsis in the United States from 1979 through 2000. *N Engl J Med* 2003;348(16):1546-54.
20. Fuseini H, Newcomb DC. Mechanisms Driving Gender Differences in Asthma. *Curr Allergy Asthma Rep* 2017;17(3):19.
21. Borkhoff CM, Hawker GA, Kreder HJ, et al. The effect of patients' sex on physicians' recommendations for total knee arthroplasty. *Cmaj* 2008;178(6):681-7.
22. Hawker GA, Wright JG, Coyte PC, et al. Differences between men and women in the rate of use of hip and knee arthroplasty. *N Engl J Med* 2000;342(14):1016-22.

For peer review only - http://bmjopen.bmj.com/site/about/guidelines.xhtml

23. Cannon MJ, Warner L, Taddei JA, et al. What can go wrong when you assume that correlated data are independent: an illustration from the evaluation of a childhood health intervention in Brazil. *Stat Med* 2001;20(9-10):1461-7.

For peer review only

Figure legends

Figure 1 Flow diagram of the study population

Figure 2 Associations of sex with ED diagnostics and management in the total study population, adjusted for age, triage urgency and clinical presentation (boys as reference group)

For peer review only

For peer review only - http://bmjopen.bmj.com/site/about/guidelines.xhtml

Tables

Table 1 Baseline characteristics of the study population (percentages are column totals)

	Boys<br>n=63,042	Girls<br>n=53,715
Age, no. (%)		
0 – < 1 yrs	10,289 (16)	8,009 (15)
1 – < 12 yrs	44,233 (70)	36,929 (69)
≥ 12 yrs	8,520 (14)	8,777 (16)
MTS urgency, no. (%)		
Emergent | Very urgent	7,532 (12)	5,232 (10)
Urgent	17,583 (28)	14,839 (28)
Standard | Non-urgent	37,927 (60)	33,644 (63)
Clinical presentation, no (%)		
Cardiac	688 (1)	710 (1)
Dermatological	8,179 (13)	6,443 (12)
Ear, Nose and Throat	5,808 (9)	5,754 (11)
Gastrointestinal	9,346 (15)	8,871 (17)
Neurologic or psychiatric	2,437 (4)	2,254 (4)
Respiratory	8,218 (13)	5,742 (11)
Trauma or musculoskeletal	11,587 (18)	9,045 (17)
General malaise	12,934 (21)	11,412 (21)
Uro- or gynaecological	1,315 (2)	1,268 (2)
Other	2530 (4)	2,216 (4)
Diagnostics, no. (%)		
Laboratory	12,461 (20)	11,597 (22)
Imaging	13,386 (21)	11,216 (21)
Medication, no. (%)		
Inhalation	5,177 (8)	3,389 (6)
Intravenous	4,901 (8)	4,143 (8)
Disposition, n (%)		
Mortality at the ED	12 (<0.1)	4 (<0.1)
ICU admission	369 (0.6)	299 (0.6)

For peer review only

Hospital admission	6,447 (10)	4,965 (9)
Discharge / other	56,214 (89)	48,447 (90)

Percentages are column totals

For peer review only

For peer review only - http://bmjopen.bmj.com/site/about/guidelines.xhtml

Table 2 Sex-specific differences in age, triage and clinical presentation

	Boys n=63,042	Range over hospitals	Girls n=53,715	Range over hospitals
Age				
0 – < 1 y	10,289 (56)	53% - 60%	8,009 (44)	40% - 47%
1 – < 12 y	44,233 (54)	53% - 59%	36,929 (46)	41% - 47%
≥ 12 y	8,520 (49)	43% - 54%	8,777 (51)	46% - 57%
Triage urgency				
Emergent | Very urgent	7,532 (59)	54% - 63%	5,232 (47)	37% - 46%
Urgent	17,583 (54)	51% - 58%	14,839 (46)	42% - 49%
Standard | Non-urgent	37,927 (53)		33,644 (41)	
Clinical presentation				
Cardiac	688 (49)	46% - 61%	710 (51)	39% - 54%
Dermatological	8,179 (56)	53% - 61%	6,443 (44)	39% - 47%
Ear, Nose and Throat	5,808 (50)	48% - 59%	5,754 (50)	41% - 52%
Gastrointestinal	9,346 (51)	49% - 57%	8,871 (49)	43% - 51%
Neurologic or psychiatric	2,437 (52)	50% - 55%	2,254 (48)	45% - 50%
Respiratory	8,218 (59)	57% - 64%	5,742 (41)	36% 43%
Trauma or musculoskeletal	11,587 (56)	54% - 59%	9,045 (44)	41% - 46%
General malaise	12,934 (53)	52% - 56%	11,412 (47)	44% - 48%
Uro- or gynaecological	1,315 (51)	40% - 80%	1,268 (49)	20% - 60%
Other	2530 (53)	52% - 56%	2,216 (47)	44% - 49%

Percentages are row totals

For peer review only - http://bmjopen.bmj.com/site/about/guidelines.xhtml

Table 3 Associations of sex with ED diagnostics and management in the total paediatric ED population and in subgroups based on type of clinical presentation (boys as reference group)

**Total paediatric ED population**				*Heterogeneity*	
**n = 116,757**		*Pooled OR (95%CI)* [a]	I^2	Q	p
**Laboratory tests**	**Girls**	**1.10 (1.05 – 1.15)**	**45.8%**	**7.27**	**0.12**
	**Boys**	**Reference**			
Imaging	Girls	1.01 (0.98 – 1.04)	0%	1.84	0.77
	Boys	Reference			
IV medication or fluids	Girls	0.96 (0.89 – 1.03)	50.5%	8.26	0.08
	Boys	Reference			
Hospital admission	Girls	0.99 (0.95 – 1.04)	0%	3.38	0.50
	Boys	Reference			
**Trauma or musculoskeletal**				Heterogeneity	
**n = 20,632**		Pooled OR (95%CI) [b]	I^2	Q	p
Laboratory tests	Girls	1.12 (0.94, 1.35)	39.8%	4.76	0.19
	Boys	Reference			
**Imaging**	**Girls**	**1.10 (1.01, 1.20)**	**44.9%**	**5.35**	**0.15**
	**Boys**	**Reference**			
IV medication or fluids	Girls	0.84 (0.62, 1.12)	74.2%	12.64	0.01
	Boys	Reference			
Hospital admission	Girls	0.90 (0.77, 1.05)	37.7%	4.76	0.19
	Boys	Reference			
**Gastrointestinal**				*Heterogeneity*	
**n = 18,217**		*Pooled OR (95%CI)* [b]	I^2	Q	p
Laboratory tests	Girls	1.05 (0.90, 1.21)	71.6%	12.74	0.01
	Boys	Reference			
Imaging	Girls	0.95 (0.85, 1.05)	19.1%	5.23	0.26
	Boys	Reference			
IV medication or fluids	Girls	0.92 (0.79, 1.07)	53.6%	8.44	0.08
	Boys	Reference			
Hospital admission	Girls	0.96 (0.87, 1.05)	0%	1.66	0.80
	Boys	Reference			
**Respiratory**				*Heterogeneity*	

For peer review only - http://bmjopen.bmj.com/site/about/guidelines.xhtml

n = 13,960		*Pooled OR (95%CI)* [b]	I^2	Q	p
**Laboratory tests**	**Girls**	**1.15 (1.04 – 1.26)**	**0%**	**2.51**	**0.64**
	**Boys**	**Reference**			
**Imaging**	**Girls**	**1.14 (1.05 – 1.24)**	**0%**	**2.69**	**0.61**
	**Boys**	**Reference**			
**Inhalation medication**	**Girls**	**0.79 (0.73, 0.86)**	**0%**	**2.38**	**0.67**
	**Boys**	**Reference**			
IV medication or fluids	Girls	1.10 (0.96, 1.26)	0%	2.08	0.72
	Boys	Reference			
Hospital admission	Girls	1.10 (1.00, 1.22)	0%	2.32	0.72
	Boys	Reference			
**Fever**				*Heterogeneity*	
**n = 8,782**		*Pooled OR (95%CI)* [b]	I^2	Q	p
Laboratory tests	Girls	1.12 (0.98 – 1.27)	42.8%	6.88	0.14
	Boys	Reference			
Imaging	Girls	1.03 (0.91 – 1.16)	0%	1.81	0.77
	Boys	Reference			
IV medication or fluids	Girls	1.04 (0.92 – 1.19)	0%	3.15	0.53
	Boys	Reference			
Hospital admission	Girls	1.07 (0.96 – 1.20)	0%	2.61	0.63
	Boys	Reference			

[a] Summary OR based on a random effects model where results from individual settings are pooled. Model adjusted for age, triage urgency and clinical presentation

[b] Summary OR based on a random effects model where results from individual settings are pooled. Model adjusted for age and triage urgency

For peer review only

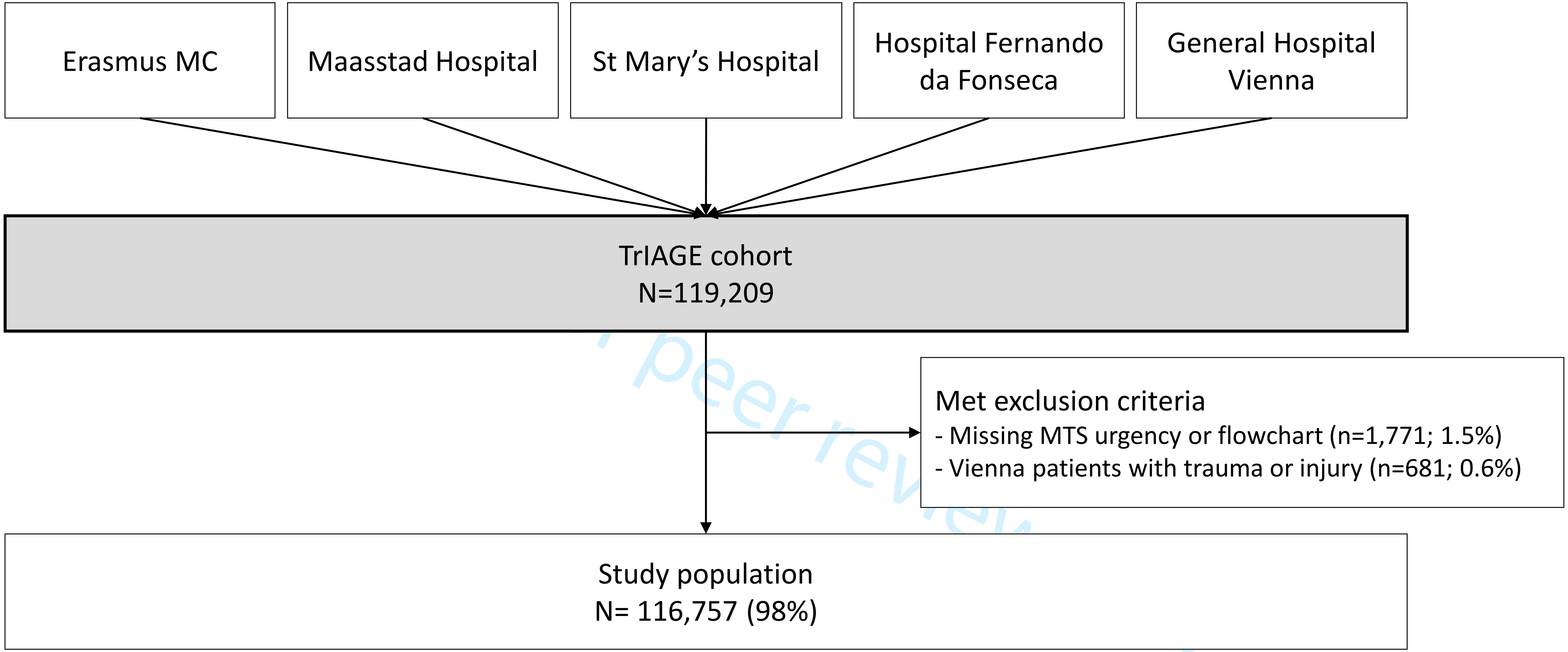
Erasmus MC
Maasstad Hospital
St Mary's Hospital
Hospital Fernando da Fonseca
General Hospital Vienna
TrIAGE cohort
N=119,209
Met exclusion criteria
- Missing MTS urgency or flowchart (n=1,771; 1.5%)
- Vienna patients with trauma or injury (n=681; 0.6%)
Study population
N= 116,757 (98%)

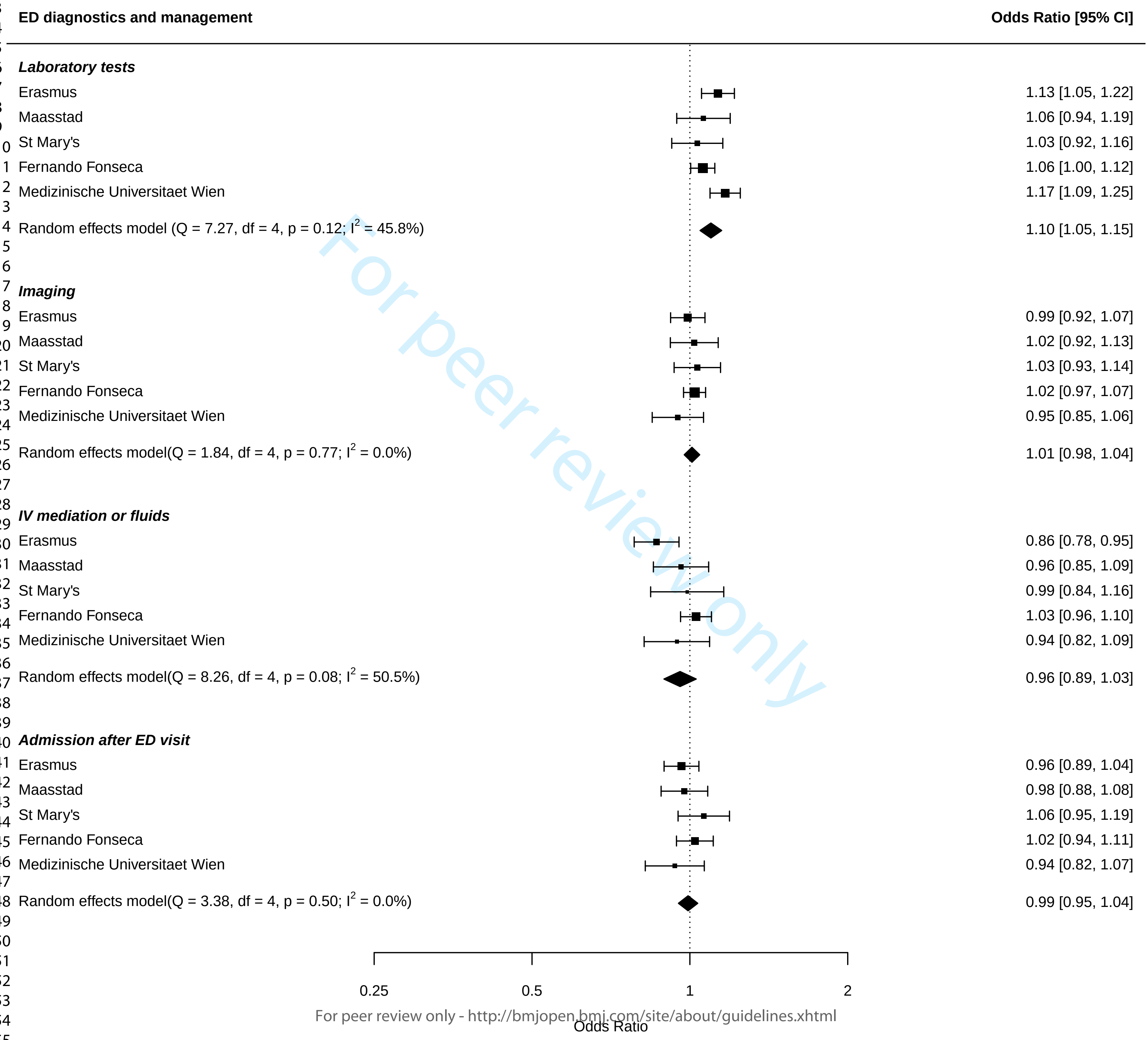

For peer review only

APPENDIX 1. PubMed literature search

We conducted a systematic literature search to identify articles describing any sex or gender differences in children visiting the emergency department (ED), including but not restricted to differences in presenting problem, disease presentation, disease severity, diagnostics, management and health care provider attitudes and behaviours.

We searched the Pubmed database for articles published from the year 2000 onwards, using search terms related to 1) age or gender (*gender [ti] or sex [ti]*); 2) emergency care (*emergency medicine [mh] or emergency service, hospital [mh] or emergency medical services [mh] or emergency department [tiab] or emergency room [tiab] or emergency medicine [tiab] or emergency service [tiab] or emergency services [tiab]*); and 3) paediatrics (*child [mh] OR infant [mh] OR adolescent [mh] OR Infant, Newborn [mh] OR child, preschool[mh] OR child* [ti] OR adolescent [ti] OR infant [ti] OR pediatr**)

This search identified a total of 134 papers of which 22 were included in the final selection. Papers were excluded because they did not (separately) address children (n=77), were not related to emergency medicine (n=27), were conducted in developing countries (n=3), or did not assess gender differences in patients (n=5).

Selected papers were divided into categories based on the research topic. The topics most commonly studied were identified: trauma and injuries including (sexual) assault (n=8)[1-8]; mental health issues, including intoxications (n=6)[9-14] and asthma (n=5)[15-19]. Other topics were general resource use (n=2)[20 21] and urinary tract infections (n=1)[22].

Table 1.1 Mental health (n=6)

Author, year	Setting	Period	n	Inclusion	Conclusion
Foto-Ozdemir, 2016	Emergency service at a Turkish Children's Hospital	-	64	Adolescents with a suicide attempt	There were no statistically significant differences between the girls and the boys with respect to the clinical characteristics of the suicide attempt and the rate of psychiatric disorders.
Kupferschmid, 2013	ED of a large university hospital in Germany	2004-2010	257	Adolescents presenting after a suicide attempt	Boys and girls differed in the methods of suicide attempt and the presence of a previous suicide attempt
Levinson, 2006	A national ED database, Israel	1996-2002	25,446	ED admissions classified as attempted suicide	Women do not attempt suicide more than men, except for 13-26 year olds.
Porter, 2016	PED in Spain	2010-2011	328 ED visits	Patients <15 years attending the pediatric emergency department who required	Males and females differ in disease presentation

For peer review only - http://bmjopen.bmj.com/site/about/guidelines.xhtml

				consultation to psychiatry service	
Rhodes, 2008	All hospital emergency departments in Ontario, Canada	April 1, 2001–March 31, 2002	+/- 2400 ED visits	All medicinal Self-Poisoning presentations	Females presented more often with poisoning.
Tonezer, 2015	Emergency Psychiatric Consultation (EPC) Service of a Brazilian hospital	2010-2013	259 ED visits	Patients <18 years who were assessed by a psychiatric emergency team	Females and males differ in the rates of psychiatric presentations and diagnoses.<br>Males needed more psychiatric medication during evaluation, but there was no difference in the rate of psychiatric hospitalization.

Table 1.2 Asthma (n=5)

**Author, year**	**Setting**	**Period**	**n**	**Inclusion**	**Conclusion**
Everage, 2010	Rhode Island Emergency Department Data	2005-2008	-	Every ED visit and hospitalization in Rhode Island to a non-federal hospital facility	Overall, women had higher rates of ED visits for asthma than men, except in the age group 0-4years
Fagan 2011	Schools in a Mississippi River community in Illinois	1994	2,693 children	Seventh through twelfth grade students	Female students reported significantly more asthma and allergic symptoms, and reported more severe symptoms and a greater number of emergency room and hospital admissions
Rosychuk, 2017	EDs in Alberta, Canada	1999-2011	115,853	Patients discharged from EDs for acute asthma	Prevalence of asthma in childhood is significantly higher in boys than girls with a higher rate of ED return. There was no difference in follow-up physician visits after ED discharge but time to first physician follow-up was shorter for girls.
Schatz, 2003	Computerized data from Southern California Kaiser-Permanente	1999 - 2000	60,694 subjects	Any patient aged 2 to 64 years, identified as having asthma in a large database	Asthma utilization and severity appear greater in males aged 2 to 13 years, somewhat greater in females aged 14 to 22 years.

For peer review only - http://bmjopen.bmj.com/site/about/guidelines.xhtml

Schatz, 2004	140 emergency departments (EDs)	Several periods between 1997 and 2001	1,602	Patients aged 2–13 years who presented to the emergency department (ED) with acute asthma	No material differences exists in acute presentation, chronic asthma characteristics, ED treatment, or ED course in boys compared to girls.

Table 1.3 Urinary tract infection (n=1)

Author, year	Setting	Period	n	Inclusion	Conclusion
Lo, 2013	ED of a general hospital, Brazil	2010	291	Patients younger than 15 years old who had clinical suspicion of UTI and collected quantitative urine culture.	Proteus mirabilis was significantly more prevalent in boys than girls

Table 1.4 Injuries and trauma, including (sexual) abuse (n=8)

Author, year	Setting	Period	n	Inclusion	Conclusion
Greenes, 2001	1) The National Pediatric Trauma Registry (NPTR) 2) A prospective cohort in an urban pediatric emergency department (ED)	1990-1999 (NTPR database)	1072 (NTPR database), 88 (cohort)	Patients ≤90 days of age who had been diagnosed with unintentional head trauma	Boys outnumber girls among infants less than 3 months of age with unintentional head trauma. These young infants appear to be passive participants in their injuries, which indicates that differences in parenting practices may account for the observed gender differences.
Hassan, 2015	ED of an urban Midwestern research and teaching hospital	2006-2010	95 children	Children 6 – 14 years old who were treated for alleged sexual abuse at the ED	There are significant differences in type of sexual abuse.
Irie, 2012	Data from 14 regional and tertiary public	2005-2008	282	Patients <= 17 years who met the inclusion criteria for the	Adolescent males were at a significantly higher risk of sustaining assault-related injuries.

For peer review only - http://bmjopen.bmj.com/site/about/guidelines.xhtml

For peer review only

	hospitals in Queensland			Queensland Trauma Registry (QTR) and were admitted to hospital for 24 h or more for the acute treatment of an accidental or assault-related injury	
Love, 2009	PED	June 2005- June 2008	3436 visits for falls of which 171 TBI's	ED visits for all injuries treated in the pediatric emergency department in which the word "fall" was listed as part of the chief complaint.	Females have a higher risk of TBI from falls than males.
Matteucci, 2007	24 regional poison centers	2002-2004	204	Pediatric snakebite injuries requiring antivenom therapy	Male children were more likely than female children to suffer Crotaline snakebites that required antivenom therapy.
McQuillan, 2006	Monklands Hospital Accident and Emergency (A&E) Department	1996–1999	19,483	0–17 year old residents of Airdrie and Coatbridge, Lanarkshire, Scotland, who attended the A&E Department with a home/leisure injury	The M:F RRR for non-sports A&E attendances remained constant throughout childhood , whilst that for sports attendances increased sharply with age The results suggest a dose–response relationship between sports participation and injury risk.
Preiss-Farzanegan, 2009	Regional trauma center emergency department.	February to September 2003	260	137 patients < 18 years presenting with sport-related mild TBI	Female minors are not at greater risk for post-concussion symptoms than males.
Ranney, 2011	Hurley Medical Center in Flint, Michigan, an inner-city, Level I trauma center ED	?	190	adolescents aged 14 to 18 years presenting to an urban ED with acute assault-related injury	Male and female adolescents with acute assault-related injuries were very similar. There was a greater prevalence of some risk factors among adolescent females.

For peer review only - http://bmjopen.bmj.com/site/about/guidelines.xhtml

Table 1.5 General resource use (n=2)

Author, year	Setting	Period	n	Inclusion	Conclusion
Henneman, 2014	urban, academic ED, USA	2003-2009	523, 882 outpatient ED encounters	Billing and demographic data from all ED visits	Resource utilization and profitability by gender were similar
Siminski, 2008	All major New South Wales EDs	2005	-	All ED visits in the administrative database	The rates of both "potentially primary care" and non-"potentially primary care" attendances are higher among men than among women..

For peer review only

References

1. Greenes DS, Wigotsky M, Schutzman SA. Gender differences in rates of unintentional head injury in the first 3 months of life. *Ambul Pediatr* 2001;1(3):178-80.
2. Hassan M, Killion C, Lewin L, et al. Gender-related sexual abuse experiences reported by children who were examined in an emergency department. *Arch Psychiatr Nurs* 2015;29(3):148-54.
3. Irie F, Lang J, Kaltner M, et al. Effects of gender, indigenous status and remoteness to health services on the occurrence of assault-related injuries in children and adolescents. *Injury* 2012;43(11):1873-80.
4. Love PF, Tepas JJ, 3rd, Wludyka PS, et al. Fall-related pediatric brain injuries: the role of race, age, and sex. *J Trauma* 2009;67(1 Suppl):S12-5.
5. Matteucci MJ, Hannum JE, Riffenburgh RH, et al. Pediatric sex group differences in location of snakebite injuries requiring antivenom therapy. *J Med Toxicol* 2007;3(3):103-6.
6. McQuillan R, Campbell H. Gender differences in adolescent injury characteristics: a population-based study of hospital A&E data. *Public Health* 2006;120(8):732-41.
7. Preiss-Farzanegan SJ, Chapman B, Wong TM, et al. The relationship between gender and postconcussion symptoms after sport-related mild traumatic brain injury. *Pm R* 2009;1(3):245-53.
8. Ranney ML, Whiteside L, Walton MA, et al. Sex differences in characteristics of adolescents presenting to the emergency department with acute assault-related injury. *Acad Emerg Med* 2011;18(10):1027-35.
9. Foto-Ozdemir D, Akdemir D, Cuhadaroglu-Cetin F. Gender differences in defense mechanisms, ways of coping with stress and sense of identity in adolescent suicide attempts. *Turk J Pediatr* 2016;58(3):271-81.
10. Kupferschmid S, Gysin-Maillart A, Buhler SK, et al. Gender differences in methods of suicide attempts and prevalence of previous suicide attempts. *Z Kinder Jugendpsychiatr Psychother* 2013;41(6):401-5.
11. Levinson D, Haklai Z, Stein N, et al. Suicide attempts in israel: age by gender analysis of a national emergency departments database. *Suicide Life Threat Behav* 2006;36(1):97-102.
12. Porter M, Gracia R, Oliva JC, et al. Mental Health Emergencies in Paediatric Services: Characteristics, Diagnostic Stability and Gender differences. *Actas Esp Psiquiatr* 2016;44(6):203-11.

For peer review only - http://bmjopen.bmj.com/site/about/guidelines.xhtml

13. Rhodes AE, Khan S, Boyle MH, et al. Sex differences in suicides among children and youth: the potential impact of help-seeking behaviour. *Can J Psychiatry* 2013;58(5):274-82.
14. Tonezer J, Muller T, Rocha GP, et al. Clinical Profile and Sex Differences in Brazilian Children and Adolescents Receiving Psychiatric Services in the Emergency Department. *Pediatr Emerg Care* 2015
15. Everage NJ, Pearlman DN, Sutton N, et al. Disparities by race/ethnicity and sex: asthma hospitalizations and emergency department visit rates in Rhode Island and Healthy People 2010 goals. *Med Health R I* 2010;93(6):177-8, 81-3.
16. Fagan JK, Scheff PA, Hryhorczuk D, et al. Prevalence of asthma and other allergic diseases in an adolescent population: association with gender and race. *Ann Allergy Asthma Immunol* 2001;86(2):177-84.
17. Rosychuk RJ, Ospina M, Zhang J, et al. Sex differences in outcomes after discharge from Alberta emergency departments for asthma: A large population-based study. *J Asthma* 2017:1-9.
18. Schatz M, Camargo CA, Jr. The relationship of sex to asthma prevalence, health care utilization, and medications in a large managed care organization. *Ann Allergy Asthma Immunol* 2003;91(6):553-8.
19. Schatz M, Clark S, Emond JA, et al. Sex differences among children 2-13 years of age presenting at the emergency department with acute asthma. *Pediatr Pulmonol* 2004;37(6):523-9.
20. Henneman PL, Nathanson BH, Ribeiro K, et al. The impact of age and gender on resource utilization and profitability in ED patients seen and released. *Am J Emerg Med* 2014;32(10):1159-67.
21. Siminski P, Bezzina AJ, Lago LP, et al. Primary care presentations at emergency departments: rates and reasons by age and sex. *Aust Health Rev* 2008;32(4):700-9.
22. Lo DS, Shieh HH, Ragazzi SL, et al. Community-acquired urinary tract infection: age and gender-dependent etiology. *J Bras Nefrol* 2013;35(2):93-8.

For peer review only

For peer review only - http://bmjopen.bmj.com/site/about/guidelines.xhtml

APPENDIX 2. Description of the different study sites

	**Erasmus MC, Rotterdam, the Netherlands**	**Maasstad Hospital, Rotterdam, the Netherlands**	**St Mary's Hospital, London, United Kingdom**	**Hospital Fernando da Fonseca, Lisbon, Portugal**	**General Hospital, Vienna, Austria**
Hospital characteristics	University hospital<br>60 paediatric beds	Teaching hospital<br>59 paediatric beds	University hospital<br>46 paediatric beds	Community hospital<br>91 paediatric beds	University hospital<br>134 paediatric beds
Catchment area	Urban<br>Mixed high and low socio-economic status	Urban<br>Generally low socio-economic status	Urban<br>Mixed high and low socio-economic status	Mixed urban and rural<br>Generally low socio-economic status	Urban<br>Mixed high and low socio-economic status
Emergency department characteristics	Before October 2014: Paediatric only<br>6500 children/year<br>Major trauma service in separate department	Mixed adult-paediatric<br>9500 children/year<br>Minor trauma service	Paediatric only<br>27,000 children/year<br>Major trauma service	Paediatric only<br>60,000 children/year<br>Major trauma service	Paediatric only<br>22,000 children/year<br>Major trauma service in separate department
Inclusion period	01-01-2012 to 31-12-2014	01-05-2014 to 01-11-2015	01-07-2014 to 28-02-2015	01-03-2014 to 28-02-2015	01-01-2014 to 31-12-2014
Study population	n = 18,594 (16%)	n = 10,584 (9%)	n = 15,556 (13%)	n = 53,175 (45%)	n = 20,300 (18%)

For peer review only - http://bmjopen.bmj.com/site/about/guidelines.xhtml

Zachariasse et al. 2020

APPENDIX 3. Distinction between clinical presentations based on selected Manchester Triage System flowchart

Clinical presentation	MTS Flowcharts
**Cardiac**	Chest pain, Palpitations
**Dermatological**	Rashes, Bites and stings, Burns and scalds, Abscesses and local infections, Wounds
**Ear, Nose and Throat**	Sore throat, Facial problems, Ear problems
**Gastrointestinal**	Abdominal pain in adults, Abdominal pain in children, Diarrhoea and vomiting, GI bleeding
**Neurologic or Psychiatric**	Headache, Fits, Collapsed adult, Behaving strangely, Overdose and poisoning, Mental illness, Self-harm, Apparently drunk
**Respiratory**	Asthma, Shortness of breath in adults, Shortness of breath in children
**Trauma or muscular**	Limb problems, Major trauma, Neck pain, Back pain, Torso injury, Falls, Assault, Head injury, Limping child
**General malaise**	Unwell adult, Unwell child, Irritable child, Worried parent, Crying baby
**Uro- or gynaecological**	Urinary problems, Testicular pain, PV bleeding, Sexually acquired infection, Pregnancy
**Other**	Major incidents primary, Dental problems, Exposure to chemicals, Foreign body, Diabetes, Eye problems, Allergy, General/Other

The classification in subgroups of clinical presentation according to a previously published study.[1] Based on clinical experience, the flowcharts “Crying baby” and “Worried parent” were moved from the category Other to General malaise.

[1]*Zachariasse JM, Seiger N, Rood PP, et al. Validity of the Manchester Triage System in emergency care: A prospective observational study. PLoS One. 2017;12(2):e0170811*

For peer review only - http://bmjopen.bmj.com/site/about/guidelines.xhtml

APPENDIX 4. Gender differences stratified by triage urgency

Figure 4.1 Differences between boys and girls over the different age categories, stratified by MTS urgency

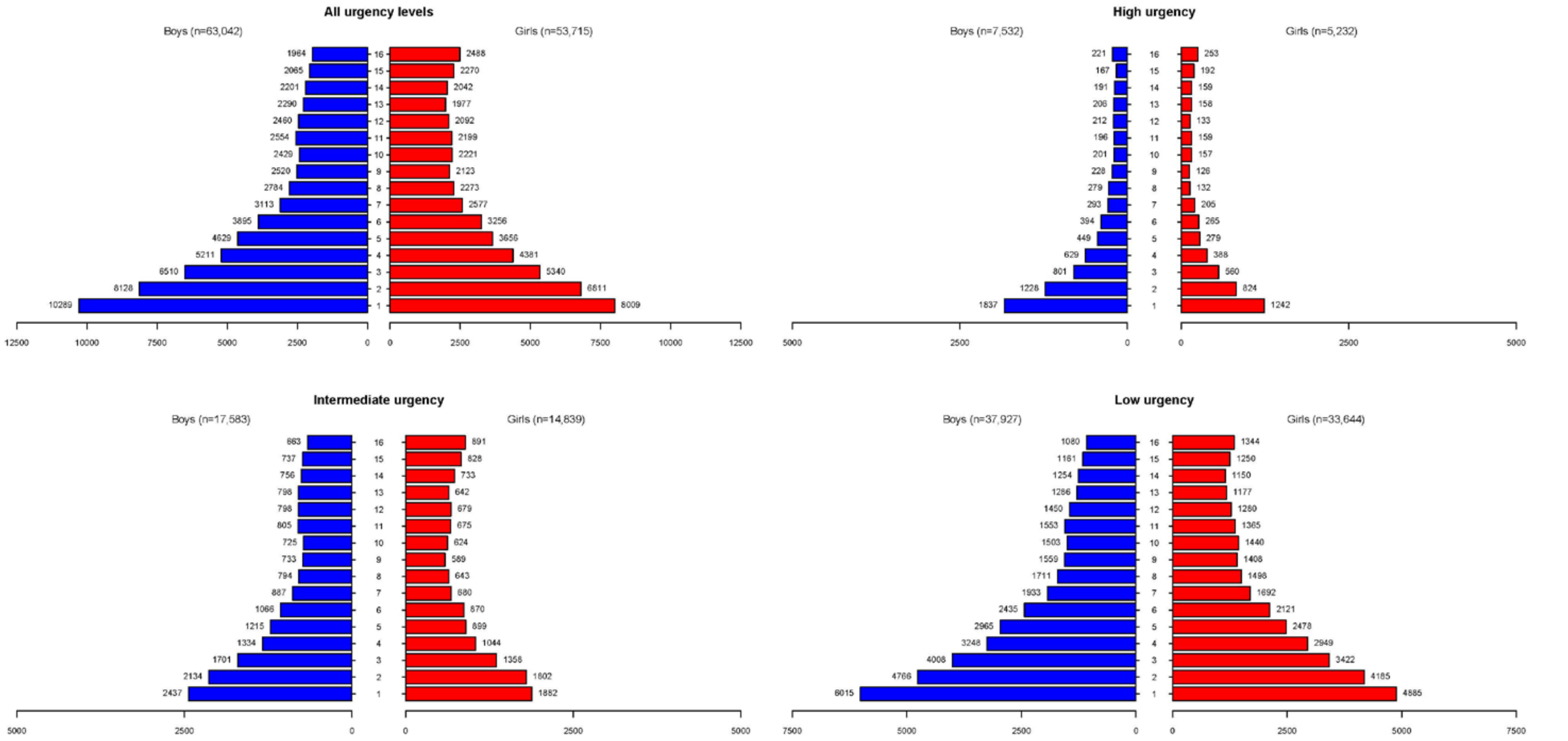

Figure 4.2 Differences between boys and girls over the different clinical presentations, stratified by MTS urgency

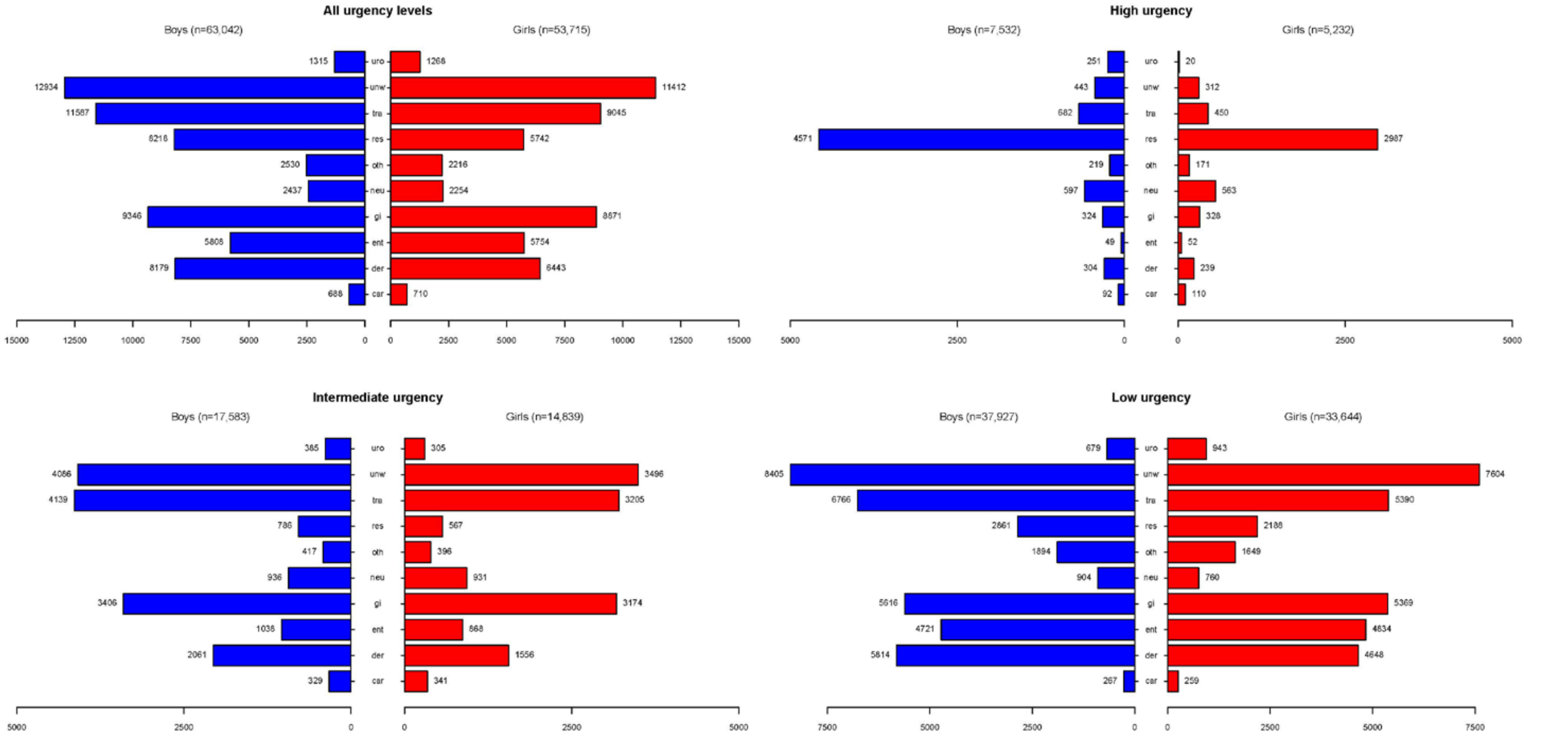

car=cardiac; der=dermatological; ent=ear, nose and throat; gi=gastrointestinal; neu=neurologic or psychiatric; oth=other; res=respiratory; tra=trauma or musculoskeletal; unw=general malaise, uro=uro- or gynaecological

For peer review only - http://bmjopen.bmj.com/site/about/guidelines.xhtml

APPENDIX 5. Associations of sex with ED diagnostics and management in subgroups

Figure 5.1 Associations of sex with ED diagnostics and management in patients with trauma or musculoskeletal problems, adjusted for age and triage urgency (boys as reference group)

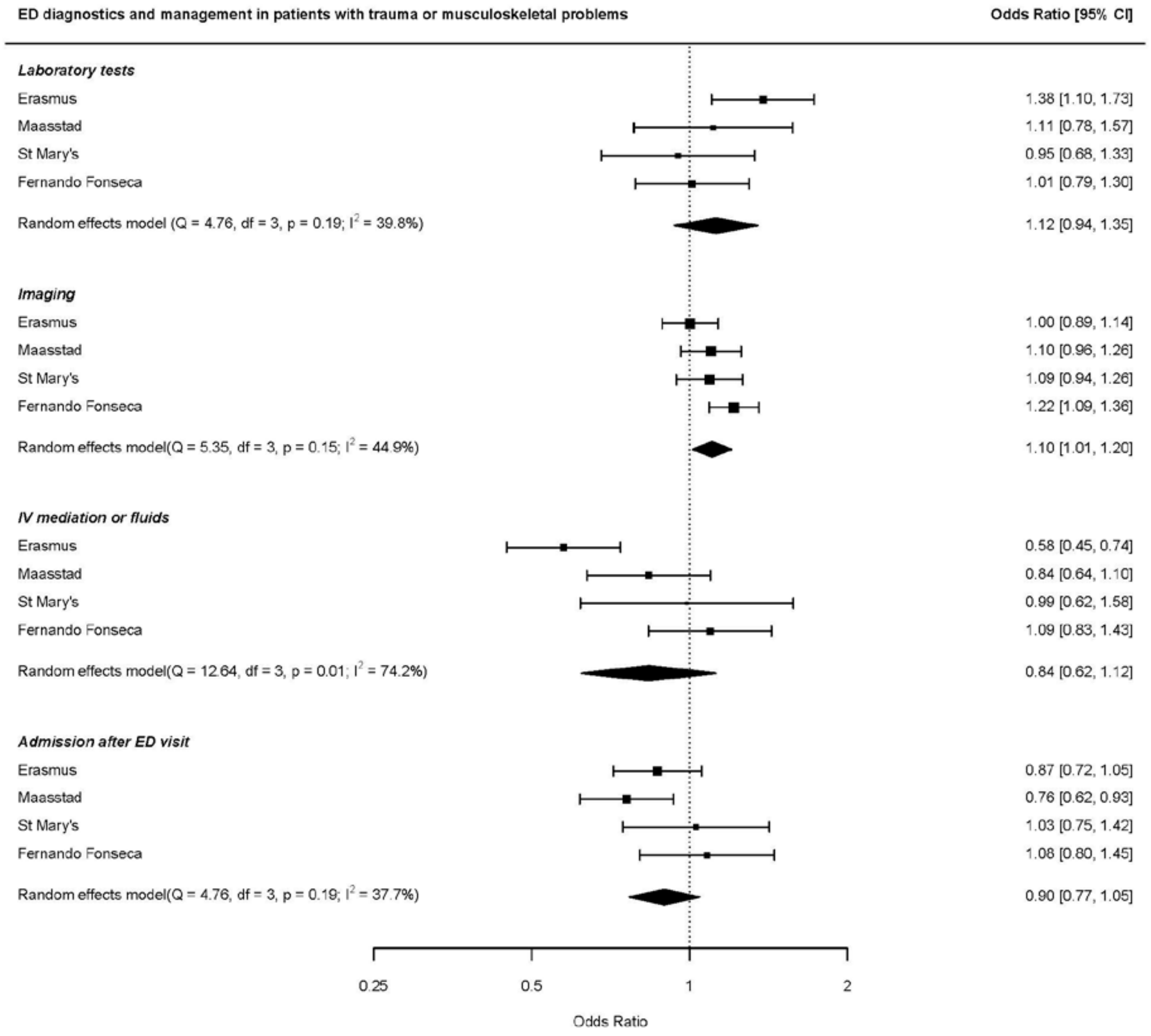

For peer review only - http://bmjopen.bmj.com/site/about/guidelines.xhtml

Figure 5.2 Associations of sex with ED diagnostics and management in patients with gastrointestinal problems, adjusted for age and triage urgency (boys as reference group)

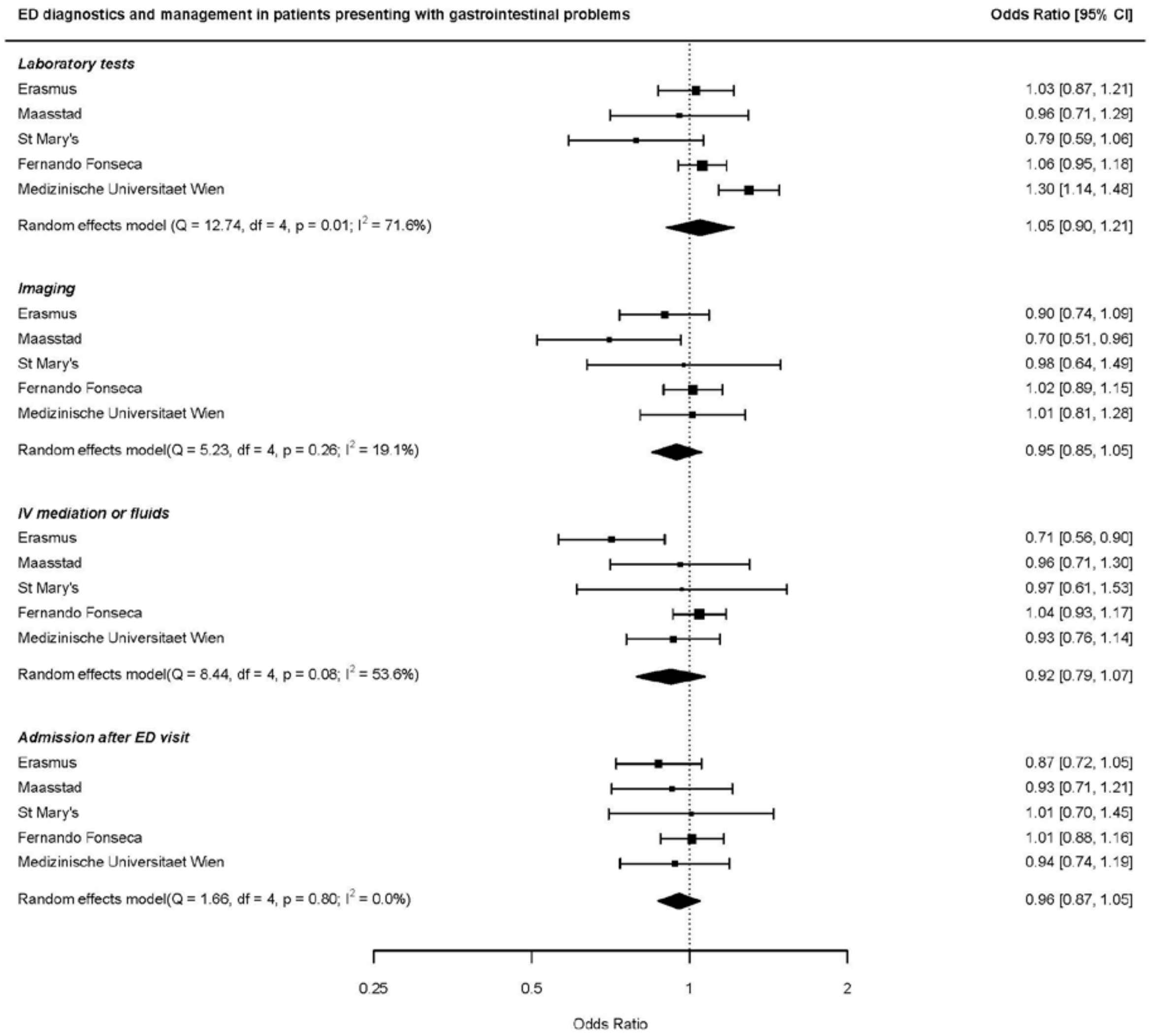

Figure 5.3 Associations of sex with ED diagnostics and management in patients with respiratory problems, adjusted for age and triage urgency (boys as reference group)

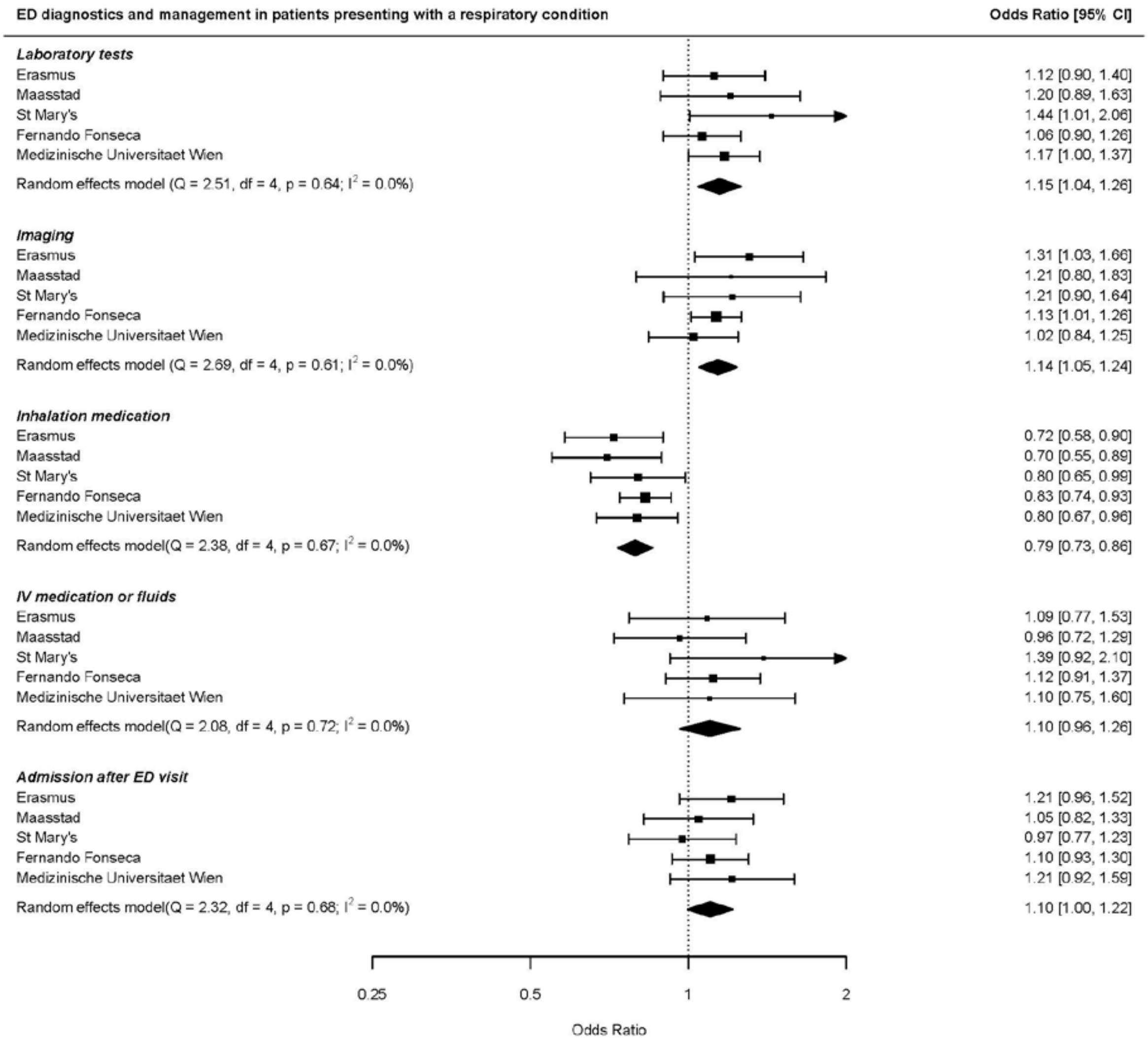

For peer review only - http://bmjopen.bmj.com/site/about/guidelines.xhtml

Figure 5.4 Associations of sex with ED diagnostics and management in patients with fever, adjusted for age and triage urgency (boys as reference group)

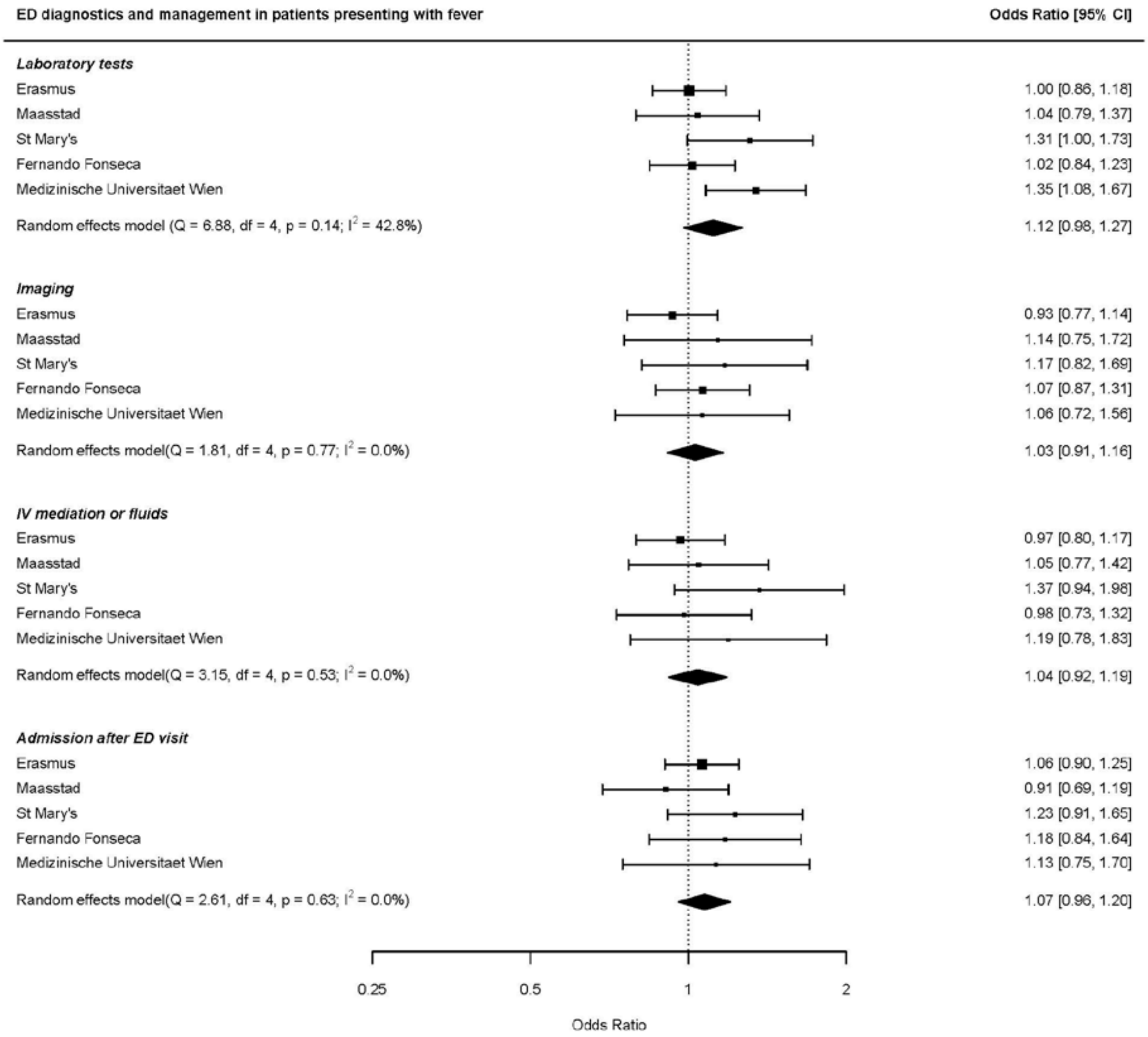

STROBE Statement—Checklist of items that should be included in reports of ***cohort studies***

"SEX-SPECIFIC DIFFERENCES IN CHILDREN ATTENDING THE EMERGENCY DEPARTMENT: PROSPECTIVE OBSERVATIONAL STUDY"*, 10-03-2020*

	Item No	Recommendation	
**Title and abstract**	1	(*a*) Indicate the study's design with a commonly used term in the title or the abstract	*The study design, a prospective observational study, is stated in the title and abstract (p1).*
		(*b*) Provide in the abstract an informative and balanced summary of what was done and what was found	*The abstract is written according to the guidelines with all required information (p5-6).*
**Introduction**			
Background/rationale	2	Explain the scientific background and rationale for the investigation being reported	*The background and rationale are described in the first two paragraphs of the introduction section (p8).*
Objectives	3	State specific objectives, including any prespecified hypotheses	*The objective is stated in the last sentence of the introduction (p8). There were no prespecified hypothesis.*
**Methods**			
Study design	4	Present key elements of study design early in the paper	*Key elements of the study design are presented in the first paragraph of the methods (p9), and in detail explained in the consequent paragraphs under subheadings "Study settings and patient population", "Data collection" and "Data analysis".*
Setting	5	Describe the setting, locations, and relevant dates, including periods of recruitment, exposure, follow-up, and data collection	*The setting, location and relevant dates are described in paragraph 'Study settings and patient population', in the methods section (p9). A detailed description of all settings is added in supplemental information 2.*
Participants	6	(*a*) Give the eligibility criteria, and the sources and methods of selection of participants. Describe methods of follow-up	*All children under the age of 16 attending the ED during the study period were eligible, as reported in the paragraph 'Study settings and patient population' in the methods section (p9).*
		(*b*) For matched studies, give matching criteria and number of exposed and unexposed	*Not applicable*
Variables	7	Clearly define all outcomes, exposures, predictors, potential confounders, and effect modifiers. Give diagnostic criteria, if applicable	*The main determinant of the study is sex, defined as male or female. The determinant and all outcomes are described in the 'Determinants'*

For peer review only - http://bmjopen.bmj.com/site/about/guidelines.xhtml

For peer review only

			paragraph in the methods section (p10).
Data sources/ measurement	8*	For each variable of interest, give sources of data and details of methods of assessment (measurement). Describe comparability of assessment methods if there is more than one group	*All variables are routinely recorded in patients' electronic health records, and automatically extracted, as reported in the paragraph 'Data collection' of the methods section (p9).*
Bias	9	Describe any efforts to address potential sources of bias	*The study is based on extraction of routinely collected data (p9). ED staff involved in the data collection were not aware of the research question, thereby minimizing information bias The number of patients with missing data was very small (2%), thereby limiting bias due to missing data (p12).*
Study size	10	Explain how the study size was arrived at	*Because of the observational nature of the study, we did not perform a formal sample size calculation. As a general rule of thumb, a regression model should have at least 10-15 cases with the outcome for each of the predictors included in the model. As can be seen in table 1, all relevant outcomes occurred in more than 1000 patients, enough to study more than 66 determinants.*
Quantitative variables	11	Explain how quantitative variables were handled in the analyses. If applicable, describe which groupings were chosen and why	*The handling of variables is described in the 'Determinants' paragraph of the methods section (p10).*
Statistical methods	12	(*a*) Describe all statistical methods, including those used to control for confounding	*The statistical methods are described in the 'Data analysis' paragraph of the methods section (p10-11).*
		(*b*) Describe any methods used to examine subgroups and interactions	*Based on the reviewer comments, we performed subgroup analyses in the most prevalent clinical presentations (p10, appendix 3).*
		(*c*) Explain how missing data were addressed	*Because the number of patients with missing data was very small (2%) we used a complete case approach, as described in the paragraph 'Study settings and patient population'(p9)'.*
		(*d*) If applicable, explain how loss to follow-up was addressed	*Not applicable*
		(*e*) Describe any sensitivity analyses	*Not applicable*
**Results**			
Participants	13*	(a) Report numbers of individuals at each stage of study—eg numbers potentially	*The patient flow is described in Figure 1 and in the first paragraph of the*

For peer review only

		eligible, examined for eligibility, confirmed eligible, included in the study, completing follow-up, and analysed	*results section (p12). All tables include the numbers of patients that were analysed.*
		(b) Give reasons for non-participation at each stage	*Not applicable*
		(c) Consider use of a flow diagram	-
Descriptive data	14*	(a) Give characteristics of study participants (eg demographic, clinical, social) and information on exposures and potential confounders	*Characteristics of the study population are described in table 1 and supplemental information 4.*
		(b) Indicate number of participants with missing data for each variable of interest	*The number of participants with missing data is provided in the flow diagram (Figure 1) and in the 'Study settings and patient population' paragraph of the methods section (p9). Missing information was limited to only two variables: triage urgency and triage flowchart (presenting problem)*
		(c) Summarise follow-up time (eg, average and total amount)	*Not applicable*
Outcome data	15*	Report numbers of outcome events or summary measures over time	*The number of patients with the outcome are reported in table 1.*
Main results	16	(*a*) Give unadjusted estimates and, if applicable, confounder-adjusted estimates and their precision (eg, 95% confidence interval). Make clear which confounders were adjusted for and why they were included	*Unadjusted estimates, i.e. absolute numbers are presented in table 1. Adjusted estimates and 95% confidence intervals are presented in figures 1 and 2. Analyses were adjusted for age and triage urgency as mentioned in the "Data analysis" paragraph (p10-11).*
		(*b*) Report category boundaries when continuous variables were categorized	*The only continuous variable, age, was maintained continues, as described in the "Determinants" paragraph of the methods section (p10).*
		(*c*) If relevant, consider translating estimates of relative risk into absolute risk for a meaningful time period	*Not applicable*
Other analyses	17	Report other analyses done—eg analyses of subgroups and interactions, and sensitivity analyses	*Pre-specified subgroup analyses were performed and described in the "Data analyses" paragraph of the methods section (p10-11), in the results section (p12-13) and in supplemental information 6.*
**Discussion**			
Key results	18	Summarise key results with reference to study objectives	*Key results are stated in the first paragraph and discussed in the subsequent paragraphs of the discussion section (p14-15).*

For peer review only - http://bmjopen.bmj.com/site/about/guidelines.xhtml

Limitations	19	Discuss limitations of the study, taking into account sources of potential bias or imprecision. Discuss both direction and magnitude of any potential bias	*Limitations of the study are addressed in the a separate "Limitations" paragraph of the discussion, (p15).*
Interpretation	20	Give a cautious overall interpretation of results considering objectives, limitations, multiplicity of analyses, results from similar studies, and other relevant evidence	*The interpretation of the study is discussed in the 2nd to 4th paragraphs of the discussion (p14-15).*
Generalisability	21	Discuss the generalisability (external validity) of the study results	*Generalisability of the study is discussed in the 2nd paragraph of the discussion (p14).*
**Other information**			
Funding	22	Give the source of funding and the role of the funders for the present study and, if applicable, for the original study on which the present article is based	*This research received no funding. This is described on the title page (p3).*

*Give information separately for exposed and unexposed groups.

Note: An Explanation and Elaboration article discusses each checklist item and gives methodological background and published examples of transparent reporting. The STROBE checklist is best used in conjunction with this article (freely available on the Web sites of PLoS Medicine at http://www.plosmedicine.org/, Annals of Internal Medicine at http://www.annals.org/, and Epidemiology at http://www.epidem.com/). Information on the STROBE Initiative is available at http://www.strobe-statement.org.

For peer review only

For peer review only - http://bmjopen.bmj.com/site/about/guidelines.xhtml

BMJ Open

Sex-Specific Differences in Children Attending the Emergency Department: Prospective Observational Study

Journal:	*BMJ Open*
Manuscript ID	bmjopen-2019-035918.R2
Article Type:	Original research
Date Submitted by the Author:	22-Jun-2020
Complete List of Authors:	Zachariasse, Joany; Erasmus MC- Sophia Children's Hospital, Department of General Paediatrics<br>Borensztajn, Dorine; Erasmus MC Sophia Children Hospital, General Paediatrics<br>Nieboer, Daan; Erasmus MC - University Medical Center Rotterdam, Public Health<br>Alves, Claudio; Hospital Fernando Fonseca, General paediatrics, Emergency Unit<br>Greber-Platzer, Susanne; Medical University of Vienna, Department of Pediatrics and Adolescent Medicine<br>Keyzer-Dekker, Claudia; Erasmus MC- Sophia Children's Hospital, Department of Pediatric Surgery<br>Maconochie, Ian; Department of Paediatric Emergency,<br>Steyerberg, Ewout; Leiden University Medical Center, Department of Medical Statistics<br>Smit, Frank; Maasstad Ziekenhuis, Department of paediatrics<br>moll, Henriette; Erasmus University Medical Center Sophia Children�s hospital, Paediatrics
**Primary Subject Heading**:	Paediatrics
Secondary Subject Heading:	Emergency medicine, Epidemiology
Keywords:	EPIDEMIOLOGY, Paediatric A&E and ambulatory care < PAEDIATRICS, PAEDIATRICS

SCHOLARONE™
Manuscripts

I, the Submitting Author has the right to grant and does grant on behalf of all authors of the Work (as defined in the below author licence), an exclusive licence and/or a non-exclusive licence for contributions from authors who are: i) UK Crown employees; ii) where BMJ has agreed a CC-BY licence shall apply, and/or iii) in accordance with the terms applicable for US Federal Government officers or employees acting as part of their official duties; on a worldwide, perpetual, irrevocable, royalty-free basis to BMJ Publishing Group Ltd ("BMJ") its licensees and where the relevant Journal is co-owned by BMJ to the co-owners of the Journal, to publish the Work in this journal and any other BMJ products and to exploit all rights, as set out in our licence.

The Submitting Author accepts and understands that any supply made under these terms is made by BMJ to the Submitting Author unless you are acting as an employee on behalf of your employer or a postgraduate student of an affiliated institution which is paying any applicable article publishing charge ("APC") for Open Access articles. Where the Submitting Author wishes to make the Work available on an Open Access basis (and intends to pay the relevant APC), the terms of reuse of such Open Access shall be governed by a Creative Commons licence – details of these licences and which Creative Commons licence will apply to this Work are set out in our licence referred to above.

Other than as permitted in any relevant BMJ Author's Self Archiving Policies, I confirm this Work has not been accepted for publication elsewhere, is not being considered for publication elsewhere and does not duplicate material already published. I confirm all authors consent to publication of this Work and authorise the granting of this licence.

For peer review only

Sex-Specific Differences in Children Attending the Emergency Department: Prospective Observational Study

Joany M Zachariasse, Dorine M Borensztajn, Daan Nieboer, Claudio F Alves, Susanne Greber-Platzer, Claudia M G Keyzer – Dekker, Ian K Maconochie, Ewout W Steyerberg, Frank J Smit, Henriëtte A Moll

Author affiliations:

Department of General Paediatrics, Erasmus MC- Sophia Children's Hospital, University Medical Centre Rotterdam, PO Box 2060, 3000 CB Rotterdam, The Netherlands

Joany M Zachariasse

PhD-student

Department of General Paediatrics, Erasmus MC- Sophia Children's Hospital, University Medical Centre Rotterdam, PO Box 2060, 3000 CB Rotterdam, The Netherlands

Dorine M Borensztajn

Paediatrician

Department of Public Health, Erasmus MC-University Medical Center Rotterdam, P.O. Box 2040

3000 CA Rotterdam The Netherlands

Daan Nieboer

PhD-student

Department of Paediatrics, Emergency Unit, Hospital Professor Doutor Fernando da Fonseca, EPE, IC 19, 2720-276 Amadora, Lisbon, Portugal

Claudio F Alves

Paediatrician

For peer review only - http://bmjopen.bmj.com/site/about/guidelines.xhtml

For peer review only

Medical University Vienna, Department of Pediatrics and Adolescent Medicine, Währinger Gürtel 16, 1090, Vienna, Austria

Susanne Greber-Platzer

Professor

Department of Pediatric Surgery, Erasmus MC-University Medical Center Rotterdam, PO Box 2060, 3000 CB Rotterdam, The Netherlands

Claudia M G Keyzer - Dekker

Paediatric Surgeon

Department of Paediatric Emergency Medicine, Imperial College NHS Healthcare Trust, London, United Kingdom

Ian K Maconochie

Associate professor

Department of Medical Statistics and Bioinformatics, Leiden University Medical Center, the Netherlands

and

Department of Public Health, Erasmus MC-University Medical Center Rotterdam, P.O. Box 2040, 3000 CA Rotterdamm The Netherlands

Ewout Steyerberg

Professor

Department of Paediatrics, Maasstad Hospital, P.O. Box 9100, 3007 AC Rotterdam, The Netherlands

Frank J Smit

Paediatrician

For peer review only

For peer review only - http://bmjopen.bmj.com/site/about/guidelines.xhtml

Department of General Paediatrics, Erasmus MC- Sophia Children's Hospital, University Medical Centre Rotterdam, PO Box 2060, 3000 CB Rotterdam, The Netherlands

Henriëtte A Moll

Professor

Corresponding author:

Prof. Dr. Henriëtte A. Moll

h.a.moll@erasmusmc.nl

Keywords [MeSH]: sex, emergency medicine, child, epidemiology, prospective studies

Word count: 2554

Word count abstract: 297

Abbreviations:

CI - confidence interval

ED – emergency department

IOM – Institute of Medicine

MTS – Manchester Triage System

OR – odds ratio

Funding: This research received no specific grant from any funding agency in the public, commercial or not-for-profit sectors.

Ethical approval: The research protocol was reviewed and approved by the local medical ethics committees and requirement for informed consent was waived.

Competing interests: The authors declare they have no potential conflicts of interest to disclose.

For peer review only - http://bmjopen.bmj.com/site/about/guidelines.xhtml

Contributorship statement: JZ contributed to the conceptualization and design of the study, carried out the analyses, and drafted the initial manuscript. DB, FS, CA, CK, IM, and SG contributed to the conceptualization and design of the study, coordinated the data collection, contributed to interpretation of the data, and critically reviewed the manuscript. DN and ES contributed to the conceptualization and design of the study, supervised the analyses, and critically reviewed the manuscript. HM contributed to the conceptualization and design of the study, contributed to interpretation of the data, critically reviewed the manuscript and supervised the study. All authors approved the final manuscript as submitted and agree to be accountable for all aspects of the work.

Transparency declaration: The lead author affirms that this manuscript is an honest, accurate, and transparent account of the study being reported; that no important aspects of the study have been omitted; and that any discrepancies from the study as planned have been explained.

Copyright licence: The Corresponding Author has the right to grant on behalf of all authors and does grant on behalf of all authors, a worldwide licence to the Publishers and its licensees in perpetuity, in all forms, formats and media (whether known now or created in the future), to i) publish, reproduce, distribute, display and store the Contribution, ii) translate the Contribution into other languages, create adaptations, reprints, include within collections and create summaries, extract and/or, abstracts of the Contributions, iii) create any other derivative work(s) based on the Contribution, iv) to exploit all subsidiary rights in the Contribution, v) the inclusion of electronic links from the Contribution to third party material where-ever it may be located; and vi) licence any third party to do any or all of the above.

Dating sharing: Data from this study are available upon request to the corresponding author of the study (h.a.moll@erasmusmc.nl), subject to local rules and regulations.

Acknowledgements: We gratefully acknowledge Rikke Jorgensen, paediatric research nurse (St. Mary's Hospital, London) and Pinky Rose Espina, paediatric resident (General Hospital, Vienna) for their assistance with data collection for this study.

For peer review only - http://bmjopen.bmj.com/site/about/guidelines.xhtml

Abstract

Objective To assess the role of sex in the presentation and management of children attending the emergency department (ED).

Design The TrIAGE project, a prospective observational study based on curated electronic health record data

Setting Five diverse European hospitals in four countries (Austria, Netherlands, Portugal, United Kingdom)

Participants All consecutive paediatric ED visits of children under the age of 16 during the study period (8 to 36 months between 2012-2015)

Main outcome measures The association between sex (male or female) and diagnostic tests and disease management in general paediatric ED visits and in subgroups presenting with trauma or musculoskeletal, gastrointestinal, and respiratory problems and fever. Results from the different hospitals were pooled in a random effects meta-analysis.

Results 116,172 ED visits were included of which 63,042 (54%) by boys and 53,715 (46%) by girls. Boys accounted for the majority of ED visits in childhood, and girls in adolescence. After adjusting for age, triage urgency and clinical presentation, girls had more laboratory tests compared with boys (pooled OR 1.10, 95%CI 1.05-1.15). Additionally, girls had more laboratory tests in ED visits for respiratory problems (pooled OR 1.15, 95%CI 1.04-1.26) and more imaging in visits for trauma or musculoskeletal problems (pooled OR 1.10, 95%CI 1.01-1.20) and respiratory conditions (pooled OR 1.14, 95%CI 1.05-1.24). Girls with respiratory problems were less often treated with inhalation medication (pooled OR 0.76, 95%CI 0.70-0.83). There was no difference in hospital admission between the sexes (pooled OR 0.99, 95%CI 0.95-1.04).

Conclusion In childhood, boys represent the majority of ED visits and they receive more inhalation medication. Unexpectedly, girls receive more diagnostic tests compared with boys . Further research is needed to investigate whether this is due to pathophysiological differences and differences in disease course, whether girls present signs and symptoms differently, or whether sociocultural factors are responsible.

For peer review only - http://bmjopen.bmj.com/site/about/guidelines.xhtml

For peer review only

Strengths and limitations of the study

- The present study was based on a large, multicentre and international cohort of more than 100,000 emergency department visits

- The study is the first to assess the role of sex in the prevalence and type of clinical presentations and in the diagnostics and management of children attending the emergency department

- Due to the use of standardized, routinely collected data, only general patterns and broad subgroups of patients could be evaluated

- Our cohort of ED visits included repeat visits from the same child, which could have led to underestimation of the standard errors and potentially to wider confidence intervals

- Only two hospitals in our study included patients with major trauma, which limits generalizability to this subgroup of patients

For peer review only

Introduction

There is increasing evidence that differences between men and women are important in the epidemiology, pathophysiology, treatment and outcome of many diseases.[1-3] The Institute of Medicine (IOM) has emphasized that sex is an important variable that should be considered in biomedical and health related research.[4] Sex refers to the biological differences between men and women, while gender refers to a broader concept including social and cultural distinctions associated with a given sex.[3] In the area of emergency medicine, sex and gender-specific differences have been found in adults, in topics as broad as the clinical presentation and outcomes of acute myocardial infarction[5], the prevalence and survival of out-of-hospital cardiac arrest[6] and the epidemiology of sports-related injuries.[7]

In children, research on the impact of sex and gender on health is scarce, particularly in the field of emergency medicine. Only twenty-two relevant articles described sex or gender differences in children related to emergency medicine between January 2000 and November 2017, according to PubMed (Appendix 1). Of these, only two studies described differences between sexes in overall emergency department resource use or management. Both studies observed higher ED attendance rates in boys compared with girls, but did not address specific types of clinical presentations or evaluate differences in disease management or outcome.[8 9] Other studies focused on specific disease groups such as trauma and injuries, mental health related conditions, and asthma. In these areas studies report conflicting results regarding differences in the rate of ED visits between boys and girls. Diagnosis and management were poorly studied. Only one study in children with asthma looked at differences in ED management and did not find a difference in treatment between boys and girls. [10]

The aim of the current study is to assess the role of sex in the clinical presentation, diagnostics and management in the general population of children attending the emergency department (ED). Through this study, we aim to gain more insight in sex-specific differences in paediatric emergency medicine and identify areas for future research.

For peer review only - http://bmjopen.bmj.com/site/about/guidelines.xhtml

Methods

This study was embedded in the TrIAGE project, a prospective observational study, based on electronic health record data, in five EDs in four European countries (the Netherlands, United Kingdom, Austria, Portugal). The study was approved by the medical ethical committees of the participating institutions: Medical Ethics Committee Erasmus MC (MEC-2013-567), Board of Directors Maasstad Ziekenhuis (L2013-103), Imperial College London Joint Research Compliance Office (14/WA/1051), Comissão de Ética para a Saúde do Hospital Prof. Dr. Fernando Fonseca EPE (Estudo Clínico TrIAGE – Parecer Favorável), Ethik Kommission Medizinische der Medizinischen Unversität Wien (EK Nr: 1405/2014). All waived the requirement for informed consent.

Study settings and patient population

In the TrIAGE study, 119,209 consecutive ED visits of children and adolescents under the age of 16 years were included. Enrolment took place during a period of 8 to 36 months between 2012 and 2015 in five diverse study sites (Appendix 2). We restricted our analysis to ED visits with complete triage data, thereby excluding all visits with missing triage categories or missing presenting problem. The General Hospital Vienna only included ED visits for medical complaints because the majority of trauma patients were seen in the department of traumatology. A small proportion of low urgent trauma cases were still seen in the ED by the "in-house paediatrician" and we excluded these remaining patients to reduce selection bias.

Data collection

The TrIAGE project is based on routinely collected, standardized electronic health record data. At the start of the study, a set of minimally required variables was determined. Completeness and accuracy of the data was discussed during a site visit and telephone meetings using a checklist quality control. With posters, newsletters and presentations, nurses were encouraged to complete the full medical records including vital sign measurements.

During the study period, nurses and physicians entered the clinical data in the medical or nursing records. After data extraction, careful data harmonization and quality checks were performed.

For peer review only - http://bmjopen.bmj.com/site/about/guidelines.xhtml

Determinants

In all participating hospitals, sex (male or female) and age were routinely registered for each patient as part of the administrative process.

We used triage data to determine type of presenting problem and triage urgency. All participating hospitals used the Manchester Triage System (MTS). This flowchart-based emergency medical triage system is the most commonly used triage system in Europe. In the MTS, the triage nurse is required to select a flowchart for each patient, representing the chief complaint, such as *Shortness of Breath* or *Wounds*. Each flowchart consists of signs and symptoms named discriminators that are ranked by priority. The nurse then gathers information on the discriminators from top to bottom. Selection of a discriminator allocates the patient to the related urgency category ranging from "Immediate" (0 minutes maximum waiting time) to "Non-urgent" (240 minutes maximum waiting time).[11]

Age, triage urgency and clinical presentation were considered as potential confounding variables in the relationship between sex and disease management. Age was maintained as a continuous variable. Triage urgency was defined as the MTS urgency category and placed into three groups: high urgent (MTS category immediate or high urgent), urgent (MTS category urgent) and low urgent (MTS category standard or non-urgent). Three hospitals (Erasmus MC, General Hospital Vienna and Hospital Fernando da Fonseca) implemented MTS modifications. To ensure consistency among hospitals, the urgency levels according to MTS version 3 with Dutch modifications for children with fever were modelled in all hospitals.[11 12] We distinguished ten clinical presentations, based on the main problem presented during triage according to MTS flowchart (Appendix 3).

For peer review only

Data analysis

In descriptive analyses, differences between male and female sex in the type and severity of problems with which they presented at the ED were explored.

Multivariable logistic regression models were used to assess the association between sex and diagnostics and management in the ED for each study site. We selected laboratory tests and imaging as important markers for diagnostics, and inhalation medication, intravenous medication or fluids and

For peer review only - http://bmjopen.bmj.com/site/about/guidelines.xhtml

hospital admission as markers for disease management. Analyses were adjusted for age, triage urgency and clinical presentation, and boys were determined as the reference group. ED visits were treated as independent in the analyses although some children likely had repeated visits and those visits could not be distinguished in this study. The results of the individual hospitals were pooled in a random effects meta-analysis and presented in forest plots. ORs were considered statistically significant when the 95% confidence interval (CI) did not include 1. Heterogeneity among settings was assessed using the Cochran's Q statistic and the I^2 statistic.

We performed subgroup analyses in subgroups of children presenting with trauma or musculoskeletal problems, children presenting with fever, children presenting with gastro-intestinal problems and children presenting with shortness of breath. Fever was defined as either a temperature of ≥ 38.5°C at the ED or the item "fever" selected as triage discriminator. Shortness of breath, gastrointestinal and trauma or musculoskeletal problems were defined based on the triage flowchart. We selected these four clinical presentations because they were among the largest subgroups of patients and represent important clinical entities in children.

Statistical analyses were performed in SPSS Statistics version 21.0 (IBM Corp, Armonk, NY, USA) and R version 3.4.2 (R Foundation, Vienna, Austria). The package metafor was used to conduct the random effects meta-analysis and create forest plots.

Patient and public involvement

There was no patient involvement in the design of the study or the writing of the manuscript. We plan to disseminate the results to patient organisations, clinicians and policy makers working in emergency care upon publication.

For peer review only

For peer review only - http://bmjopen.bmj.com/site/about/guidelines.xhtml

Results

Of all 119,209 ED visits included in the TrIAGE cohort, 116,757 ED visits (98%) were included in the study (Figure 1). There was no significant difference between the sexes in the proportion of excluded ED visits (Pearson's Chi-Square(1) = 0.08, p-value 0.77. General characteristics of the study population are presented in Table 1.

The relation between sex and age, triage urgency and clinical presentation

In the total study population, the sex imbalance in number of ED visits changed with age. In childhood, consistently more boys than girls attended the ED, and the ratio reversed in adolescence (Table 2). Boys accounted for the majority of visits in all triage urgency levels, most evidently in the high urgency categories. They had the highest proportion of ED visits with dermatological, respiratory and traumatic complaints, while in children presenting with cardiac, ENT, gastrointestinal and neurologic conditions, the boy to girl ratio was almost equal.

We further explored the relation between sex and triage urgency. The higher rate of boys' ED visits in childhood and girls' ED visits in adolescence was consistent among the different urgency categories. Also, the sex-specific differences in the different types of clinical presentation were similar in the high, intermediate and low urgency categories. An exception was the subgroup of uro- and gynaecological presentations where there were almost only boys prioritized as high urgency. Also notable was the subgroup of respiratory conditions that represents the largest population of high urgency visits with a 1.5 times higher proportion of boys compared to girls (Appendix 4).

Sex-specific differences in diagnostics at the ED

In the general population of children visiting the ED, adjusted for age, triage urgency and clinical presentation, girls had more laboratory tests at the ED than boys (pooled OR 1.10, 95%CI 1.05-1.15) (Table 3, Figure 2). This finding was consistent in all hospitals, although there was some evidence for heterogeneity (Q-statistic 7.27, p=0.12, I^2 statistic 45.8%). Significant higher rates of laboratory testing in girls were also seen in the subgroup of ED visits for respiratory problems (pooled OR 1.15, 95%CI 1.04-

For peer review only - http://bmjopen.bmj.com/site/about/guidelines.xhtml

1.26) and trends were observed in ED visits for trauma or musculoskeletal problems (pooled OR 1.12, 95%CI 0.94-1.35) and fever (pooled OR 1.12, 95%CI 0.98-1.27) (Appendix 5).

In the overall population, no differences in imaging between the sexes were found. However, in the subgroups of ED visits for trauma or musculoskeletal problems and respiratory conditions, more imaging was conducted in girls (pooled OR 1.10, 95%CI 1.01-1.20 and pooled OR 1.14, 95%CI 1.05-1.24 respectively).

Sex-specific differences in management at the ED

In the general population of children attending the ED, adjusted for age, triage urgency and clinical presentation, there was no difference in the rate of IV medication or fluids (OR 0.96, 95%CI 0.89-1.03) and in the rate of hospital admission (OR 0.99, 95%CI 0.95-1.04) (Table 3, Figure 2). Neither was this difference seen in one of the subgroups. Remarkably, there was a large difference in the use of inhalation medication in the subgroup of ED visits for respiratory conditions. In this subgroup, girls received less inhalation medication (pooled OR 0.76, 95%CI 0.70-0.83) (Appendix 5).

For peer review only

For peer review only - http://bmjopen.bmj.com/site/about/guidelines.xhtml

Discussion

In a European cohort of more than 100,000 ED visits, boys accounted for the majority of ED visits in childhood, while in adolescence, the male to female ratio reversed. We found evidence that more diagnostic tests were conducted in girls compared with boys: girls had more laboratory tests overall (pooled OR 1.10, 95%CI 1.05-1.15), and in the subgroup of ED visits for respiratory problems (pooled OR 1.15, 95%CI 1.04-1.26) while girls received more imaging in the ED visits for trauma or musculoskeletal problems (pooled OR 1.10, 95%CI 1.01-1.20) and for respiratory conditions (pooled OR 1.14, 95%CI 1.05-1.24). Girls, however, received less inhalation medication when presenting with respiratory problems (pooled OR 0.76, 95%CI 0.70-0.83). There was no difference between sexes in the odds of hospital admission (OR 0.99, 95%CI 0.95-1.04).

This is the first study that assesses the role of sex in paediatric emergency medicine, using a large, multicentre and international cohort. Despite the diversity in study sites, results were comparable across the participating EDs, supporting generalizability of the findings.

The higher attendance rate of boys in European EDs is consistent with findings from the US National Hospital Ambulatory Medical Surveys.[13] Diverse biological explanations have been proposed, such as an increased susceptibility to infections and higher risk of lung disease in men, due to male sex hormones.[14-16] Behavioural, social and cultural factors might lead to differences in the number and types of ED visits as well. These may include differences in activities, risk taking behaviour, parenting practices, and different referral decisions by primary care providers.[17 18] Sex hormones have been considered responsible for differences in lung development and differences in prevalence and severity of lung diseases.[14 15 19] A higher prevalence of asthma in boys during childhood has previously been reported, although reports in emergency care have been inconsistent.[16 20] However, boys' increased susceptibility to respiratory conditions could indicate a more severe disease course, possibly explaining the higher rate of inhalation medications used in our study.

Beside sex differences in disease presentations, our study found differences in diagnostics between boys and girls. We did not find any previous publication with a similar finding. Sex-specific differences in the need for diagnostic tests could be caused by differences in disease severity, but may also be attributed to differences in clinical presentation. Boys and girls may differ regarding the presenting signs and symptoms, the way they express pain or distress, or their ability to phrase their symptoms. Diagnostic uncertainty due to a-specific disease presentation may warrant more diagnostic tests. Finally, it is

For peer review only - http://bmjopen.bmj.com/site/about/guidelines.xhtml

possible that provider attitude subconsciously differs in boys compared with girls. In adults, implicit gender bias in diagnostics and treatment has been found in observational research and standardized case scenarios. [21 22] Our analyses are exploratory and our findings require further research. Future studies are needed that address sex-specific differences in the conducting of diagnostic tests. These studies should aim to elucidate whether differences in the need for diagnostics are due to pathophysiological mechanisms and differences in disease course, whether girls present signs and symptoms differently compared with boys, or whether there are other sociocultural factors responsible.

Limitations

These results should be interpreted in light of the limitations of using standardized routinely collected data. Detailed information such as specific types of diagnoses or results from diagnostic tests was not available. Therefore, only general patterns and broad subgroups of patients were evaluated. Furthermore, it is possible that observed differences in diagnostics and management between boys and girls are confounded by sex-specific differences in type and severity of presenting conditions. The analyses were adjusted for age, triage urgency and clinical presentation, and the findings were analysed in specific subtypes of clinical presentations. There may, however, be remaining variation not captured in urgency and presenting problem. Additionally, our cohort of ED visits included repeat visits from the same child which are not independent. In general, ignoring correlated data does not influence effect estimates such as odds ratios, but it can lead bias in the standard errors.[23] By not taking into account the correlation of visits from the same subject we are likely to underestimate the standard errors of our effect sizes. Such underestimation could potentially lead to smaller confidence intervals than would be obtained when analyses are adjusted for repeat visits from the same child. A previous study shows a revisit rate of approximately 21% in a representative sample of data from U.S. EDs.[24] Finally, only two hospitals in our study included patients with major trauma which limits generalizability to this subgroup of patients.

Conclusion

In children, the role of sex and gender on health is largely unknown and research assessing sex -specific differences is scare. Our study found that in childhood boys more often present to the ED compared with girls, while in adolescence this ratio is reversed. The higher need for inhalation medication in boys may represent a higher susceptibility for or a more severe course of respiratory infections. Unexpectedly, girls receive more diagnostic tests compared with boys. Future studies should focus on

For peer review only - http://bmjopen.bmj.com/site/about/guidelines.xhtml

the role of sex and gender in specific conditions and determine whether there are pathophysiological differences in disease course and severity, whether girls present signs and symptoms differently or whether there are social and cultural factors responsible.

References

1. Doyal L. Sex, gender, and health: the need for a new approach. *Bmj* 2001;323(7320):1061-3.
2. Greenberg MR, Safdar B, Choo EK, et al. Future directions in sex- and Gender-specific Emergency Medicine. *Acad Emerg Med* 2014;21(12):1339-42.
3. Institute of Medicine Committee on Women's Health Research. Women's Health Research: Progress, Pitfalls and Promise. 2010
4. Institute of Medicine Committee on Understanding the Biology of Sex and Gender Differences. Exploring the Biological Contributions to Human Health: Does Sex Matter? 2001
5. Mehta LS, Beckie TM, DeVon HA, et al. Acute Myocardial Infarction in Women: A Scientific Statement From the American Heart Association. *Circulation* 2016;133(9):916-47.
6. Wigginton JG, Perman SM, Barr GC, et al. Sex- and gender-specific research priorities in cardiovascular resuscitation: proceedings from the 2014 Academic Emergency Medicine Consensus Conference Cardiovascular Resuscitation Research Workgroup. *Acad Emerg Med* 2014;21(12):1343-9.
7. Raukar NP, Zonfrillo MR, Kane K, et al. Gender- and sex-specific sports-related injury research in emergency medicine: a consensus on future research direction and focused application. *Acad Emerg Med* 2014;21(12):1370-9.
8. Henneman PL, Nathanson BH, Ribeiro K, et al. The impact of age and gender on resource utilization and profitability in ED patients seen and released. *Am J Emerg Med* 2014;32(10):1159-67.
9. Siminski P, Bezzina AJ, Lago LP, et al. Primary care presentations at emergency departments: rates and reasons by age and sex. *Aust Health Rev* 2008;32(4):700-9.
10. Schatz M, Clark S, Emond JA, et al. Sex differences among children 2-13 years of age presenting at the emergency department with acute asthma. *Pediatr Pulmonol* 2004;37(6):523-9.
11. Mackway-Jones K, Marsden J, Windle J. Emergency Triage: John Wiley & Sons, Ltd. 2014.
12. van Veen M, Steyerberg EW, Van't Klooster M, et al. The Manchester triage system: improvements for paediatric emergency care. *Emerg Med J* 2012;29(8):654-9.
13. Rui P, Okeyode T. National Ambulatory Medical Care Survey: 2015 State and National Summary tables. Available from: http://www.cdc.gov/nchs/ahcd/ahcd/products.htm. Accessed 11-06-2017.
14. Klein SL. The effects of hormones on sex differences in infection: from genes to behavior. *Neurosci Biobehav Rev* 2000;24(6):627-38.
15. Wichmann MW, Inthorn D, Andress HJ, et al. Incidence and mortality of severe sepsis in surgical intensive care patients: the influence of patient gender on disease process and outcome. *Intensive Care Med* 2000;26(2):167-72.
16. Carey MA, Card JW, Voltz JW, et al. It's all about sex: gender, lung development and lung disease. *Trends Endocrinol Metab* 2007;18(8):308-13.
17. Hartzog TH, Timerding BL, Alson RL. Pediatric trauma: enabling factors, social situations, and outcome. *Acad Emerg Med* 1996;3(3):213-20.
18. Greenes DS, Wigotsky M, Schutzman SA. Gender differences in rates of unintentional head injury in the first 3 months of life. *Ambul Pediatr* 2001;1(3):178-80.
19. Martin GS, Mannino DM, Eaton S, et al. The epidemiology of sepsis in the United States from 1979 through 2000. *N Engl J Med* 2003;348(16):1546-54.

For peer review only - http://bmjopen.bmj.com/site/about/guidelines.xhtml

20. Fuseini H, Newcomb DC. Mechanisms Driving Gender Differences in Asthma. *Curr Allergy Asthma Rep* 2017;17(3):19.
21. Borkhoff CM, Hawker GA, Kreder HJ, et al. The effect of patients' sex on physicians' recommendations for total knee arthroplasty. *Cmaj* 2008;178(6):681-7.
22. Hawker GA, Wright JG, Coyte PC, et al. Differences between men and women in the rate of use of hip and knee arthroplasty. *N Engl J Med* 2000;342(14):1016-22.
23. Cannon MJ, Warner L, Taddei JA, et al. What can go wrong when you assume that correlated data are independent: an illustration from the evaluation of a childhood health intervention in Brazil. *Stat Med* 2001;20(9-10):1461-7.
24. Schlichting LE, Rogers ML, Gjelsvik A, et al. Pediatric Emergency Department Utilization and Reliance by Insurance Coverage in the United States. *Acad Emerg Med* 2017;24(12):1483-90.

For peer review only

Figure legends

Figure 1 Flow diagram of the study population

Figure 2 Associations of sex with ED diagnostics and management in the total study population, adjusted for age, triage urgency and clinical presentation (boys as reference group)

For peer review only

For peer review only - http://bmjopen.bmj.com/site/about/guidelines.xhtml

Tables

Table 1 Baseline characteristics of the study population (percentages are column totals)

	Boys<br>n=63,042	Girls<br>n=53,715
Age, no. (%)		
0 – < 1 yrs	10,289 (16)	8,009 (15)
1 – < 12 yrs	44,233 (70)	36,929 (69)
≥ 12 yrs	8,520 (14)	8,777 (16)
MTS urgency, no. (%)		
Emergent | Very urgent	7,532 (12)	5,232 (10)
Urgent	17,583 (28)	14,839 (28)
Standard | Non-urgent	37,927 (60)	33,644 (63)
Clinical presentation, no (%)		
Cardiac	688 (1)	710 (1)
Dermatological	8,179 (13)	6,443 (12)
Ear, Nose and Throat	5,808 (9)	5,754 (11)
Gastrointestinal	9,346 (15)	8,871 (17)
Neurologic or psychiatric	2,437 (4)	2,254 (4)
Respiratory	8,218 (13)	5,742 (11)
Trauma or musculoskeletal	11,587 (18)	9,045 (17)
General malaise	12,934 (21)	11,412 (21)
Uro- or gynaecological	1,315 (2)	1,268 (2)
Other	2530 (4)	2,216 (4)
Diagnostics, no. (%)		
Laboratory	12,461 (20)	11,597 (22)
Imaging	13,386 (21)	11,216 (21)
Medication, no. (%)		
Inhalation	5,177 (8)	3,389 (6)
Intravenous	4,901 (8)	4,143 (8)
Disposition, n (%)		
Mortality at the ED	12 (<0.1)	4 (<0.1)
ICU admission	369 (0.6)	299 (0.6)

For peer review only - http://bmjopen.bmj.com/site/about/guidelines.xhtml

For peer review only

Hospital admission	6,447 (10)	4,965 (9)
Discharge / other	56,214 (89)	48,447 (90)

Percentages are column totals

For peer review only

For peer review only - http://bmjopen.bmj.com/site/about/guidelines.xhtml

Table 2 Sex-specific differences in age, triage and clinical presentation

	Boys<br>n=63,042	Range over hospitals	Girls<br>n=53,715	Range over hospitals
Age				
0 – < 1 y	10,289 (56)	53% - 60%	8,009 (44)	40% - 47%
1 – < 12 y	44,233 (54)	53% - 59%	36,929 (46)	41% - 47%
≥ 12 y	8,520 (49)	43% - 54%	8,777 (51)	46% - 57%
Triage urgency				
Emergent | Very urgent	7,532 (59)	54% - 63%	5,232 (47)	37% - 46%
Urgent	17,583 (54)	51% - 58%	14,839 (46)	42% - 49%
Standard | Non-urgent	37,927 (53)		33,644 (41)	
Clinical presentation				
Cardiac	688 (49)	46% - 61%	710 (51)	39% - 54%
Dermatological	8,179 (56)	53% - 61%	6,443 (44)	39% - 47%
Ear, Nose and Throat	5,808 (50)	48% - 59%	5,754 (50)	41% - 52%
Gastrointestinal	9,346 (51)	49% - 57%	8,871 (49)	43% - 51%
Neurologic or psychiatric	2,437 (52)	50% - 55%	2,254 (48)	45% - 50%
Respiratory	8,218 (59)	57% - 64%	5,742 (41)	36% 43%
Trauma or musculoskeletal	11,587 (56)	54% - 59%	9,045 (44)	41% - 46%
General malaise	12,934 (53)	52% - 56%	11,412 (47)	44% - 48%
Uro- or gynaecological	1,315 (51)	40% - 80%	1,268 (49)	20% - 60%
Other	2530 (53)	52% - 56%	2,216 (47)	44% - 49%

Percentages are row totals

For peer review only - http://bmjopen.bmj.com/site/about/guidelines.xhtml

Table 3 Associations of sex with ED diagnostics and management in the total paediatric ED population and in subgroups based on type of clinical presentation (boys as reference group)

**Total paediatric ED population** **n = 116,757**		*Pooled OR (95%CI)* [a]	I^2	*Heterogeneity* Q	p
**Laboratory tests**	**Girls**	**1.10 (1.05 – 1.15)**	**45.8%**	**7.27**	**0.12**
	**Boys**	**Reference**			
Imaging	Girls	1.01 (0.98 – 1.04)	0%	1.84	0.77
	Boys	Reference			
IV medication or fluids	Girls	0.96 (0.89 – 1.03)	50.5%	8.26	0.08
	Boys	Reference			
Hospital admission	Girls	0.99 (0.95 – 1.04)	0%	3.38	0.50
	Boys	Reference			
**Trauma or musculoskeletal** **n = 20,632**		Pooled OR (95%CI) [b]	I^2	Heterogeneity Q	p
Laboratory tests	Girls	1.12 (0.94, 1.35)	39.8%	4.76	0.19
	Boys	Reference			
**Imaging**	**Girls**	**1.10 (1.01, 1.20)**	**44.9%**	**5.35**	**0.15**
	**Boys**	**Reference**			
IV medication or fluids	Girls	0.84 (0.62, 1.12)	74.2%	12.64	0.01
	Boys	Reference			
Hospital admission	Girls	0.90 (0.77, 1.05)	37.7%	4.76	0.19
	Boys	Reference			
**Gastrointestinal** **n = 18,217**		*Pooled OR (95%CI)* [b]	I^2	*Heterogeneity* Q	p
Laboratory tests	Girls	1.05 (0.90, 1.21)	71.6%	12.74	0.01
	Boys	Reference			
Imaging	Girls	0.95 (0.85, 1.05)	19.1%	5.23	0.26
	Boys	Reference			
IV medication or fluids	Girls	0.92 (0.79, 1.07)	53.6%	8.44	0.08
	Boys	Reference			
Hospital admission	Girls	0.96 (0.87, 1.05)	0%	1.66	0.80
	Boys	Reference			
**Respiratory**				*Heterogeneity*	

For peer review only - http://bmjopen.bmj.com/site/about/guidelines.xhtml

n = 13,960		*Pooled OR (95%CI)* [b]	I^2	Q	p
**Laboratory tests**	**Girls**	**1.15 (1.04 – 1.26)**	**0%**	**2.51**	**0.64**
	**Boys**	**Reference**			
**Imaging**	**Girls**	**1.14 (1.05 – 1.24)**	**0%**	**2.69**	**0.61**
	**Boys**	**Reference**			
**Inhalation medication**	**Girls**	**0.79 (0.73, 0.86)**	**0%**	**2.38**	**0.67**
	**Boys**	**Reference**			
IV medication or fluids	Girls	1.10 (0.96, 1.26)	0%	2.08	0.72
	Boys	Reference			
Hospital admission	Girls	1.10 (1.00, 1.22)	0%	2.32	0.72
	Boys	Reference			
**Fever**				*Heterogeneity*	
**n = 8,782**		*Pooled OR (95%CI)* [b]	I^2	Q	p
Laboratory tests	Girls	1.12 (0.98 – 1.27)	42.8%	6.88	0.14
	Boys	Reference			
Imaging	Girls	1.03 (0.91 – 1.16)	0%	1.81	0.77
	Boys	Reference			
IV medication or fluids	Girls	1.04 (0.92 – 1.19)	0%	3.15	0.53
	Boys	Reference			
Hospital admission	Girls	1.07 (0.96 – 1.20)	0%	2.61	0.63
	Boys	Reference			

[a] Summary OR based on a random effects model where results from individual settings are pooled. Model adjusted for age, triage urgency and clinical presentation

[b] Summary OR based on a random effects model where results from individual settings are pooled. Model adjusted for age and triage urgency

For peer review only - http://bmjopen.bmj.com/site/about/guidelines.xhtml

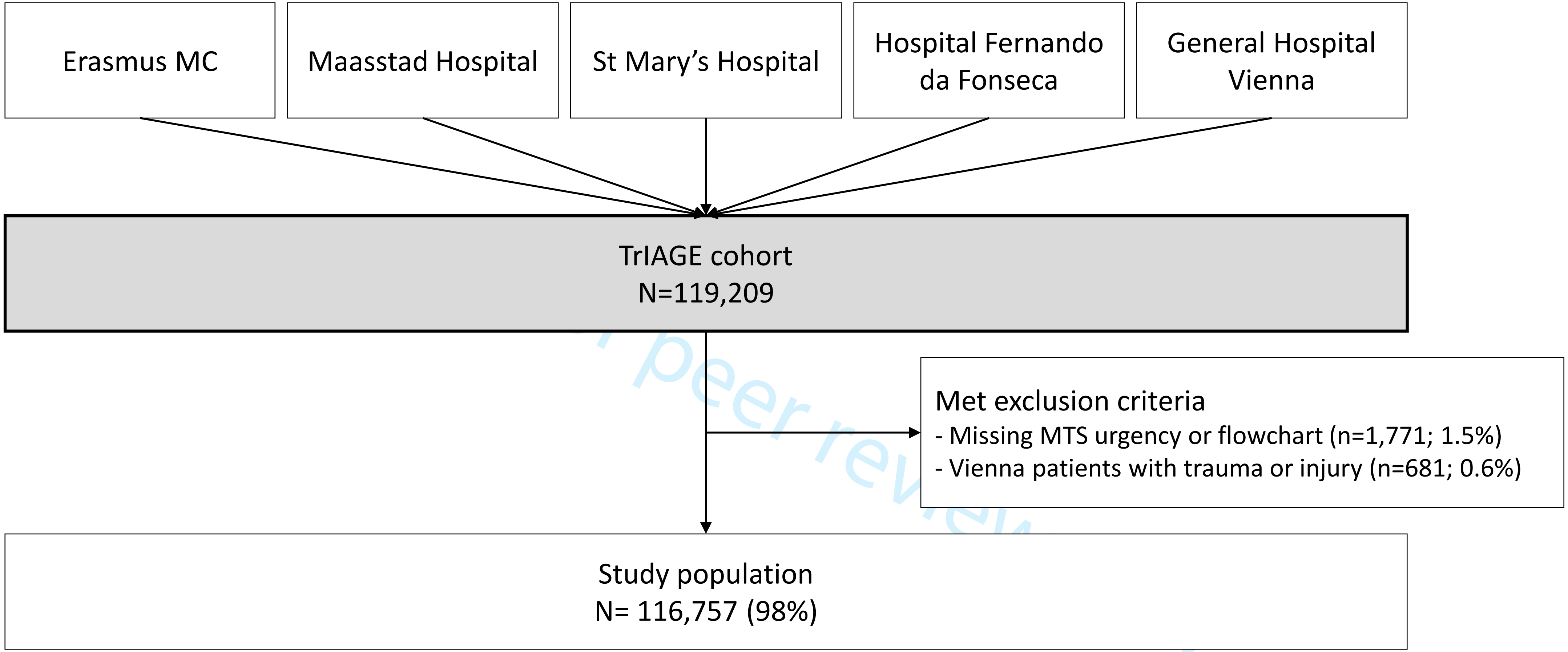
Erasmus MC
Maasstad Hospital
St Mary's Hospital
Hospital Fernando da Fonseca
General Hospital Vienna
TrIAGE cohort
N=119,209
Met exclusion criteria
- Missing MTS urgency or flowchart (n=1,771; 1.5%)
- Vienna patients with trauma or injury (n=681; 0.6%)
Study population
N= 116,757 (98%)

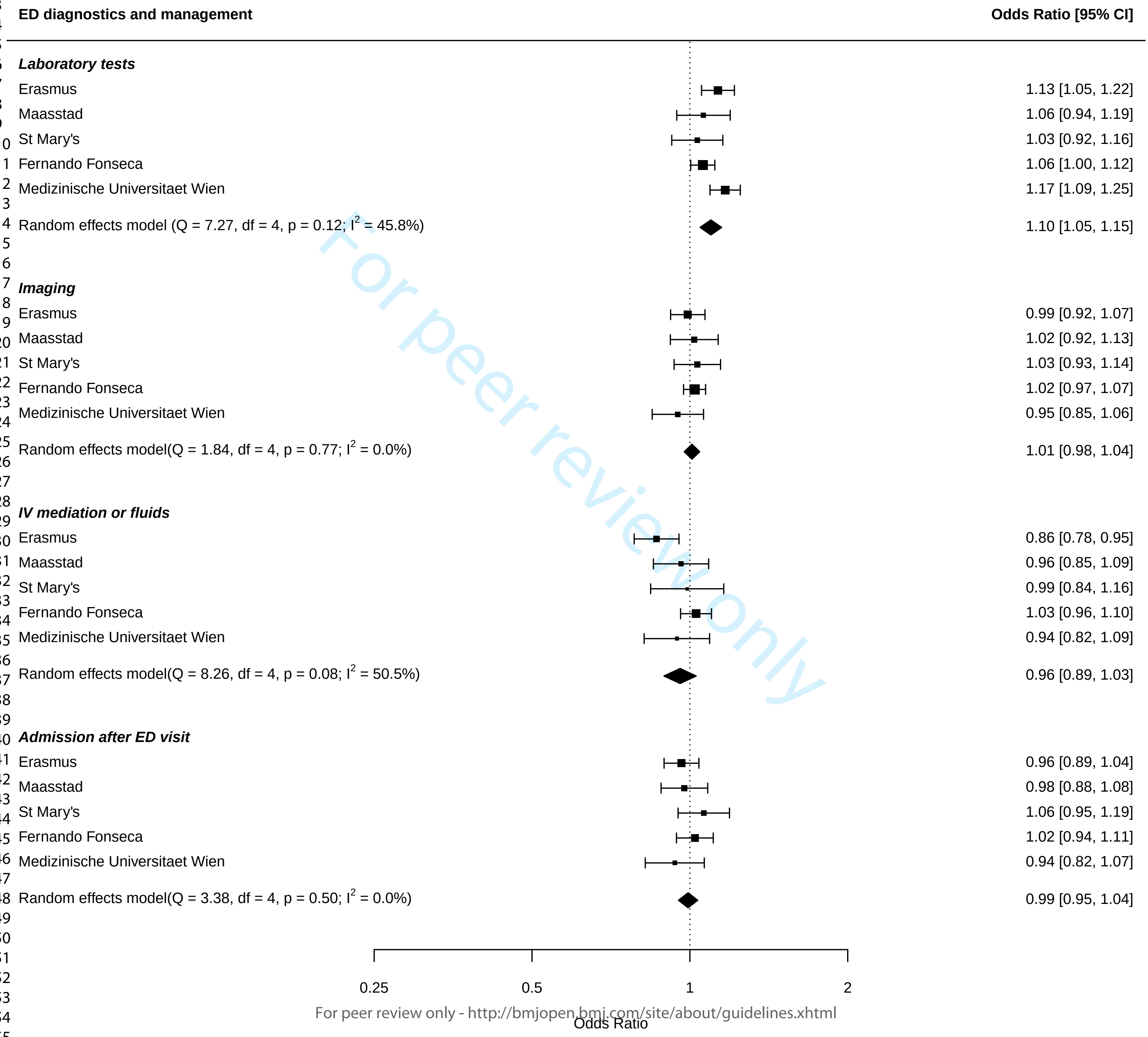

For peer review only

APPENDIX 1. PubMed literature search

We conducted a systematic literature search to identify articles describing any sex or gender differences in children visiting the emergency department (ED), including but not restricted to differences in presenting problem, disease presentation, disease severity, diagnostics, management and health care provider attitudes and behaviours.

We searched the Pubmed database for articles published from the year 2000 onwards, using search terms related to 1) age or gender (*gender [ti] or sex [ti]*); 2) emergency care (*emergency medicine [mh] or emergency service, hospital [mh] or emergency medical services [mh] or emergency department [tiab] or emergency room [tiab] or emergency medicine [tiab] or emergency service [tiab] or emergency services [tiab]*); and 3) paediatrics (*child [mh] OR infant [mh] OR adolescent [mh] OR Infant, Newborn [mh] OR child, preschool[mh] OR child* [ti] OR adolescent [ti] OR infant [ti] OR pediatr**)

This search identified a total of 134 papers of which 22 were included in the final selection. Papers were excluded because they did not (separately) address children (n=77), were not related to emergency medicine (n=27), were conducted in developing countries (n=3), or did not assess gender differences in patients (n=5).

Selected papers were divided into categories based on the research topic. The topics most commonly studied were identified: trauma and injuries including (sexual) assault (n=8)[1-8]; mental health issues, including intoxications (n=6)[9-14] and asthma (n=5)[15-19]. Other topics were general resource use (n=2)[20 21] and urinary tract infections (n=1)[22].

Table 1.1 Mental health (n=6)

Author, year	Setting	Period	n	Inclusion	Conclusion
Foto-Ozdemir, 2016	Emergency service at a Turkish Children's Hospital	-	64	Adolescents with a suicide attempt	There were no statistically significant differences between the girls and the boys with respect to the clinical characteristics of the suicide attempt and the rate of psychiatric disorders.
Kupferschmid, 2013	ED of a large university hospital in Germany	2004-2010	257	Adolescents presenting after a suicide attempt	Boys and girls differed in the methods of suicide attempt and the presence of a previous suicide attempt
Levinson, 2006	A national ED database, Israel	1996-2002	25,446	ED admissions classified as attempted suicide	Women do not attempt suicide more than men, except for 13-26 year olds.
Porter, 2016	PED in Spain	2010-2011	328 ED visits	Patients <15 years attending the pediatric emergency department who required	Males and females differ in disease presentation

For peer review only - http://bmjopen.bmj.com/site/about/guidelines.xhtml

				consultation to psychiatry service	
Rhodes, 2008	All hospital emergency departments in Ontario, Canada	April 1, 2001–March 31, 2002	+/- 2400 ED visits	All medicinal Self-Poisoning presentations	Females presented more often with poisoning.
Tonezer, 2015	Emergency Psychiatric Consultation (EPC) Service of a Brazilian hospital	2010-2013	259 ED visits	Patients <18 years who were assessed by a psychiatric emergency team	Females and males differ in the rates of psychiatric presentations and diagnoses.<br>Males needed more psychiatric medication during evaluation, but there was no difference in the rate of psychiatric hospitalization.

Table 1.2 Asthma (n=5)

**Author, year**	**Setting**	**Period**	**n**	**Inclusion**	**Conclusion**
Everage, 2010	Rhode Island Emergency Department Data	2005-2008	-	Every ED visit and hospitalization in Rhode Island to a non-federal hospital facility	Overall, women had higher rates of ED visits for asthma than men, except in the age group 0-4years
Fagan 2011	Schools in a Mississippi River community in Illinois	1994	2,693 children	Seventh through twelfth grade students	Female students reported significantly more asthma and allergic symptoms, and reported more severe symptoms and a greater number of emergency room and hospital admissions
Rosychuk, 2017	EDs in Alberta, Canada	1999-2011	115,853	Patients discharged from EDs for acute asthma	Prevalence of asthma in childhood is significantly higher in boys than girls with a higher rate of ED return. There was no difference in follow-up physician visits after ED discharge but time to first physician follow-up was shorter for girls.
Schatz, 2003	Computerized data from Southern California Kaiser-Permanente	1999 - 2000	60,694 subjects	Any patient aged 2 to 64 years, identified as having asthma in a large database	Asthma utilization and severity appear greater in males aged 2 to 13 years, somewhat greater in females aged 14 to 22 years.

For peer review only - http://bmjopen.bmj.com/site/about/guidelines.xhtml

Schatz, 2004	140 emergency departments (EDs)	Several periods between 1997 and 2001	1,602	Patients aged 2–13 years who presented to the emergency department (ED) with acute asthma	No material differences exists in acute presentation, chronic asthma characteristics, ED treatment, or ED course in boys compared to girls.

Table 1.3 Urinary tract infection (n=1)

Author, year	Setting	Period	n	Inclusion	Conclusion
Lo, 2013	ED of a general hospital, Brazil	2010	291	Patients younger than 15 years old who had clinical suspicion of UTI and collected quantitative urine culture.	Proteus mirabilis was significantly more prevalent in boys than girls

For peer review only

Table 1.4 Injuries and trauma, including (sexual) abuse (n=8)

Author, year	Setting	Period	n	Inclusion	Conclusion
Greenes, 2001	1) The National Pediatric Trauma Registry (NPTR)<br>2) A prospective cohort in an urban pediatric emergency department (ED)	1990-1999 (NTPR database)	1072 (NTPR database), 88 (cohort)	Patients ≤90 days of age who had been diagnosed with unintentional head trauma	Boys outnumber girls among infants less than 3 months of age with unintentional head trauma. These young infants appear to be passive participants in their injuries, which indicates that differences in parenting practices may account for the observed gender differences.
Hassan, 2015	ED of an urban Midwestern research and teaching hospital	2006-2010	95 children	Children 6 – 14 years old who were treated for alleged sexual abuse at the ED	There are significant differences in type of sexual abuse.
Irie, 2012	Data from 14 regional and tertiary public	2005-2008	282	Patients <= 17 years who met the inclusion criteria for the	Adolescent males were at a significantly higher risk of sustaining assault-related injuries.

For peer review only - http://bmjopen.bmj.com/site/about/guidelines.xhtml

	hospitals in Queensland			Queensland Trauma Registry (QTR) and were admitted to hospital for 24 h or more for the acute treatment of an accidental or assault-related injury	
Love, 2009	PED	June 2005- June 2008	3436 visits for falls of which 171 TBI's	ED visits for all injuries treated in the pediatric emergency department in which the word "fall" was listed as part of the chief complaint.	Females have a higher risk of TBI from falls than males.
Matteucci, 2007	24 regional poison centers	2002-2004	204	Pediatric snakebite injuries requiring antivenom therapy	Male children were more likely than female children to suffer Crotaline snakebites that required antivenom therapy.
McQuillan, 2006	Monklands Hospital Accident and Emergency (A&E) Department	1996–1999	19,483	0–17 year old residents of Airdrie and Coatbridge, Lanarkshire, Scotland, who attended the A&E Department with a home/leisure injury	The M:F RRR for non-sports A&E attendances remained constant throughout childhood , whilst that for sports attendances increased sharply with age The results suggest a dose–response relationship between sports participation and injury risk.
Preiss-Farzanegan, 2009	Regional trauma center emergency department.	February to September 2003	260	137 patients < 18 years presenting with sport-related mild TBI	Female minors are not at greater risk for post-concussion symptoms than males.
Ranney, 2011	Hurley Medical Center in Flint, Michigan, an inner-city, Level I trauma center ED	?	190	adolescents aged 14 to 18 years presenting to an urban ED with acute assault-related injury	Male and female adolescents with acute assault-related injuries were very similar. There was a greater prevalence of some risk factors among adolescent females.

For peer review only - http://bmjopen.bmj.com/site/about/guidelines.xhtml

Table 1.5 General resource use (n=2)

Author, year	Setting	Period	n	Inclusion	Conclusion
Henneman, 2014	urban, academic ED, USA	2003-2009	523, 882 outpatient ED encounters	Billing and demographic data from all ED visits	Resource utilization and profitability by gender were similar
Siminski, 2008	All major New South Wales EDs	2005	-	All ED visits in the administrative database	The rates of both "potentially primary care" and non-"potentially primary care" attendances are higher among men than among women..

References

1. Greenes DS, Wigotsky M, Schutzman SA. Gender differences in rates of unintentional head injury in the first 3 months of life. *Ambul Pediatr* 2001;1(3):178-80.
2. Hassan M, Killion C, Lewin L, et al. Gender-related sexual abuse experiences reported by children who were examined in an emergency department. *Arch Psychiatr Nurs* 2015;29(3):148-54.
3. Irie F, Lang J, Kaltner M, et al. Effects of gender, indigenous status and remoteness to health services on the occurrence of assault-related injuries in children and adolescents. *Injury* 2012;43(11):1873-80.
4. Love PF, Tepas JJ, 3rd, Wludyka PS, et al. Fall-related pediatric brain injuries: the role of race, age, and sex. *J Trauma* 2009;67(1 Suppl):S12-5.
5. Matteucci MJ, Hannum JE, Riffenburgh RH, et al. Pediatric sex group differences in location of snakebite injuries requiring antivenom therapy. *J Med Toxicol* 2007;3(3):103-6.
6. McQuillan R, Campbell H. Gender differences in adolescent injury characteristics: a population-based study of hospital A&E data. *Public Health* 2006;120(8):732-41.
7. Preiss-Farzanegan SJ, Chapman B, Wong TM, et al. The relationship between gender and postconcussion symptoms after sport-related mild traumatic brain injury. *Pm R* 2009;1(3):245-53.
8. Ranney ML, Whiteside L, Walton MA, et al. Sex differences in characteristics of adolescents presenting to the emergency department with acute assault-related injury. *Acad Emerg Med* 2011;18(10):1027-35.
9. Foto-Ozdemir D, Akdemir D, Cuhadaroglu-Cetin F. Gender differences in defense mechanisms, ways of coping with stress and sense of identity in adolescent suicide attempts. *Turk J Pediatr* 2016;58(3):271-81.
10. Kupferschmid S, Gysin-Maillart A, Buhler SK, et al. Gender differences in methods of suicide attempts and prevalence of previous suicide attempts. *Z Kinder Jugendpsychiatr Psychother* 2013;41(6):401-5.
11. Levinson D, Haklai Z, Stein N, et al. Suicide attempts in israel: age by gender analysis of a national emergency departments database. *Suicide Life Threat Behav* 2006;36(1):97-102.
12. Porter M, Gracia R, Oliva JC, et al. Mental Health Emergencies in Paediatric Services: Characteristics, Diagnostic Stability and Gender differences. *Actas Esp Psiquiatr* 2016;44(6):203-11.

For peer review only - http://bmjopen.bmj.com/site/about/guidelines.xhtml

13. Rhodes AE, Khan S, Boyle MH, et al. Sex differences in suicides among children and youth: the potential impact of help-seeking behaviour. *Can J Psychiatry* 2013;58(5):274-82.
14. Tonezer J, Muller T, Rocha GP, et al. Clinical Profile and Sex Differences in Brazilian Children and Adolescents Receiving Psychiatric Services in the Emergency Department. *Pediatr Emerg Care* 2015
15. Everage NJ, Pearlman DN, Sutton N, et al. Disparities by race/ethnicity and sex: asthma hospitalizations and emergency department visit rates in Rhode Island and Healthy People 2010 goals. *Med Health R I* 2010;93(6):177-8, 81-3.
16. Fagan JK, Scheff PA, Hryhorczuk D, et al. Prevalence of asthma and other allergic diseases in an adolescent population: association with gender and race. *Ann Allergy Asthma Immunol* 2001;86(2):177-84.
17. Rosychuk RJ, Ospina M, Zhang J, et al. Sex differences in outcomes after discharge from Alberta emergency departments for asthma: A large population-based study. *J Asthma* 2017:1-9.
18. Schatz M, Camargo CA, Jr. The relationship of sex to asthma prevalence, health care utilization, and medications in a large managed care organization. *Ann Allergy Asthma Immunol* 2003;91(6):553-8.
19. Schatz M, Clark S, Emond JA, et al. Sex differences among children 2-13 years of age presenting at the emergency department with acute asthma. *Pediatr Pulmonol* 2004;37(6):523-9.
20. Henneman PL, Nathanson BH, Ribeiro K, et al. The impact of age and gender on resource utilization and profitability in ED patients seen and released. *Am J Emerg Med* 2014;32(10):1159-67.
21. Siminski P, Bezzina AJ, Lago LP, et al. Primary care presentations at emergency departments: rates and reasons by age and sex. *Aust Health Rev* 2008;32(4):700-9.
22. Lo DS, Shieh HH, Ragazzi SL, et al. Community-acquired urinary tract infection: age and gender-dependent etiology. *J Bras Nefrol* 2013;35(2):93-8.

For peer review only

For peer review only - http://bmjopen.bmj.com/site/about/guidelines.xhtml

APPENDIX 2. Description of the different study sites

	**Erasmus MC, Rotterdam, the Netherlands**	**Maasstad Hospital, Rotterdam, the Netherlands**	**St Mary's Hospital, London, United Kingdom**	**Hospital Fernando da Fonseca, Lisbon, Portugal**	**General Hospital, Vienna, Austria**
Hospital characteristics	University hospital<br>60 paediatric beds	Teaching hospital<br>59 paediatric beds	University hospital<br>46 paediatric beds	Community hospital<br>91 paediatric beds	University hospital<br>134 paediatric beds
Catchment area	Urban<br>Mixed high and low socio-economic status	Urban<br>Generally low socio-economic status	Urban<br>Mixed high and low socio-economic status	Mixed urban and rural<br>Generally low socio-economic status	Urban<br>Mixed high and low socio-economic status
Emergency department characteristics	Before October 2014: Paediatric only<br>6500 children/year<br>Major trauma service in separate department	Mixed adult-paediatric<br>9500 children/year<br>Minor trauma service	Paediatric only<br>27,000 children/year<br>Major trauma service	Paediatric only<br>60,000 children/year<br>Major trauma service	Paediatric only<br>22,000 children/year<br>Major trauma service in separate department
Inclusion period	01-01-2012 to 31-12-2014	01-05-2014 to 01-11-2015	01-07-2014 to 28-02-2015	01-03-2014 to 28-02-2015	01-01-2014 to 31-12-2014
Study population	n = 18,594 (16%)	n = 10,584 (9%)	n = 15,556 (13%)	n = 53,175 (45%)	n = 20,300 (18%)

For peer review only - http://bmjopen.bmj.com/site/about/guidelines.xhtml

APPENDIX 3. Distinction between clinical presentations based on selected Manchester Triage System flowchart

Clinical presentation	MTS Flowcharts
**Cardiac**	Chest pain, Palpitations
**Dermatological**	Rashes, Bites and stings, Burns and scalds, Abscesses and local infections, Wounds
**Ear, Nose and Throat**	Sore throat, Facial problems, Ear problems
**Gastrointestinal**	Abdominal pain in adults, Abdominal pain in children, Diarrhoea and vomiting, GI bleeding
**Neurologic or Psychiatric**	Headache, Fits, Collapsed adult, Behaving strangely, Overdose and poisoning, Mental illness, Self-harm, Apparently drunk
**Respiratory**	Asthma, Shortness of breath in adults, Shortness of breath in children
**Trauma or muscular**	Limb problems, Major trauma, Neck pain, Back pain, Torso injury, Falls, Assault, Head injury, Limping child
**General malaise**	Unwell adult, Unwell child, Irritable child, Worried parent, Crying baby
**Uro- or gynaecological**	Urinary problems, Testicular pain, PV bleeding, Sexually acquired infection, Pregnancy
**Other**	Major incidents primary, Dental problems, Exposure to chemicals, Foreign body, Diabetes, Eye problems, Allergy, General/Other

The classification in subgroups of clinical presentation according to a previously published study.[1] Based on clinical experience, the flowcharts "Crying baby" and "Worried parent" were moved from the category Other to General malaise.

[1]*Zachariasse JM, Seiger N, Rood PP, et al. Validity of the Manchester Triage System in emergency care: A prospective observational study. PLoS One. 2017;12(2):e0170811*

For peer review only - http://bmjopen.bmj.com/site/about/guidelines.xhtml

APPENDIX 4. Gender differences stratified by triage urgency

Figure 4.1 Differences between boys and girls over the different age categories, stratified by MTS urgency

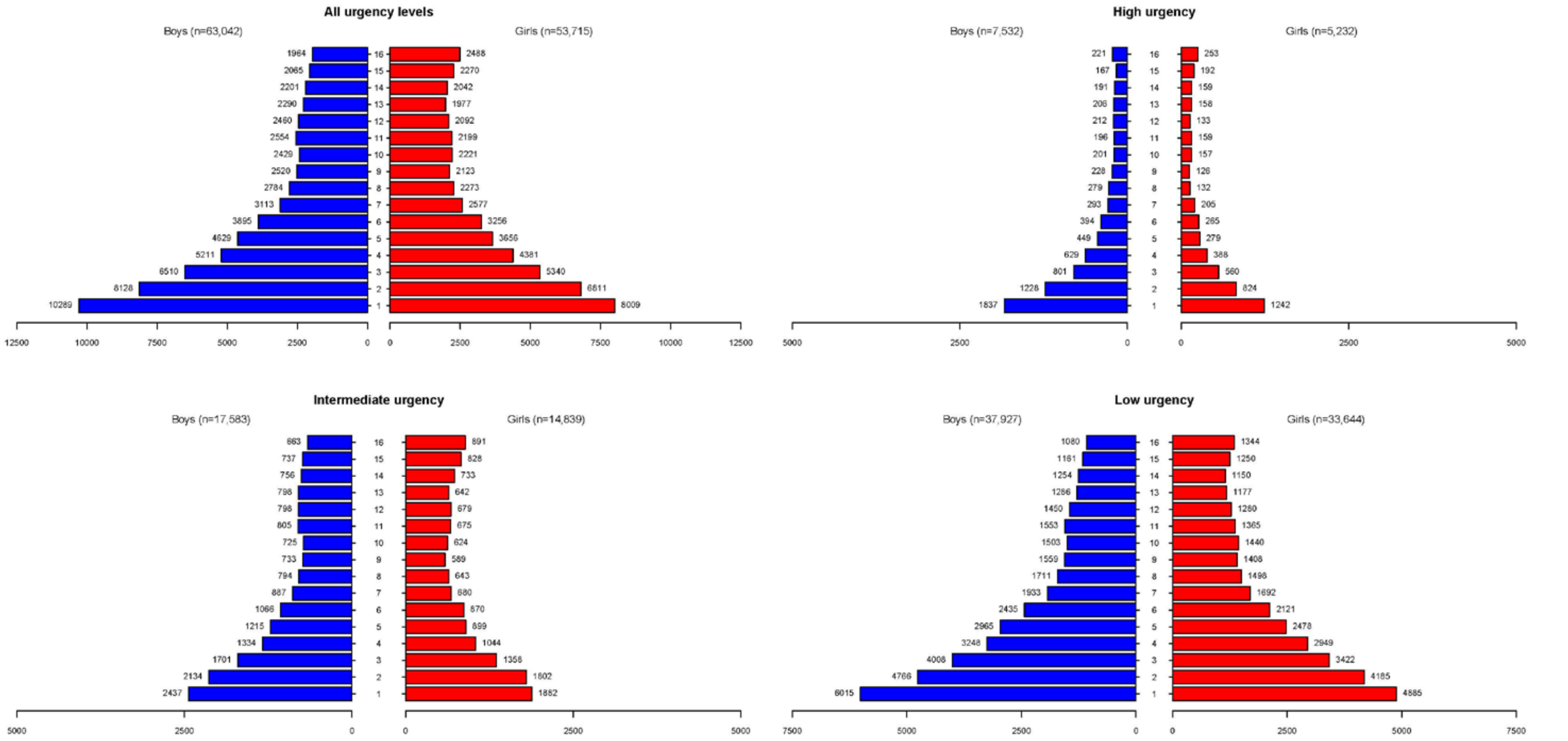

For peer review only - http://bmjopen.bmj.com/site/about/guidelines.xhtml

Figure 4.2 Differences between boys and girls over the different clinical presentations, stratified by MTS urgency

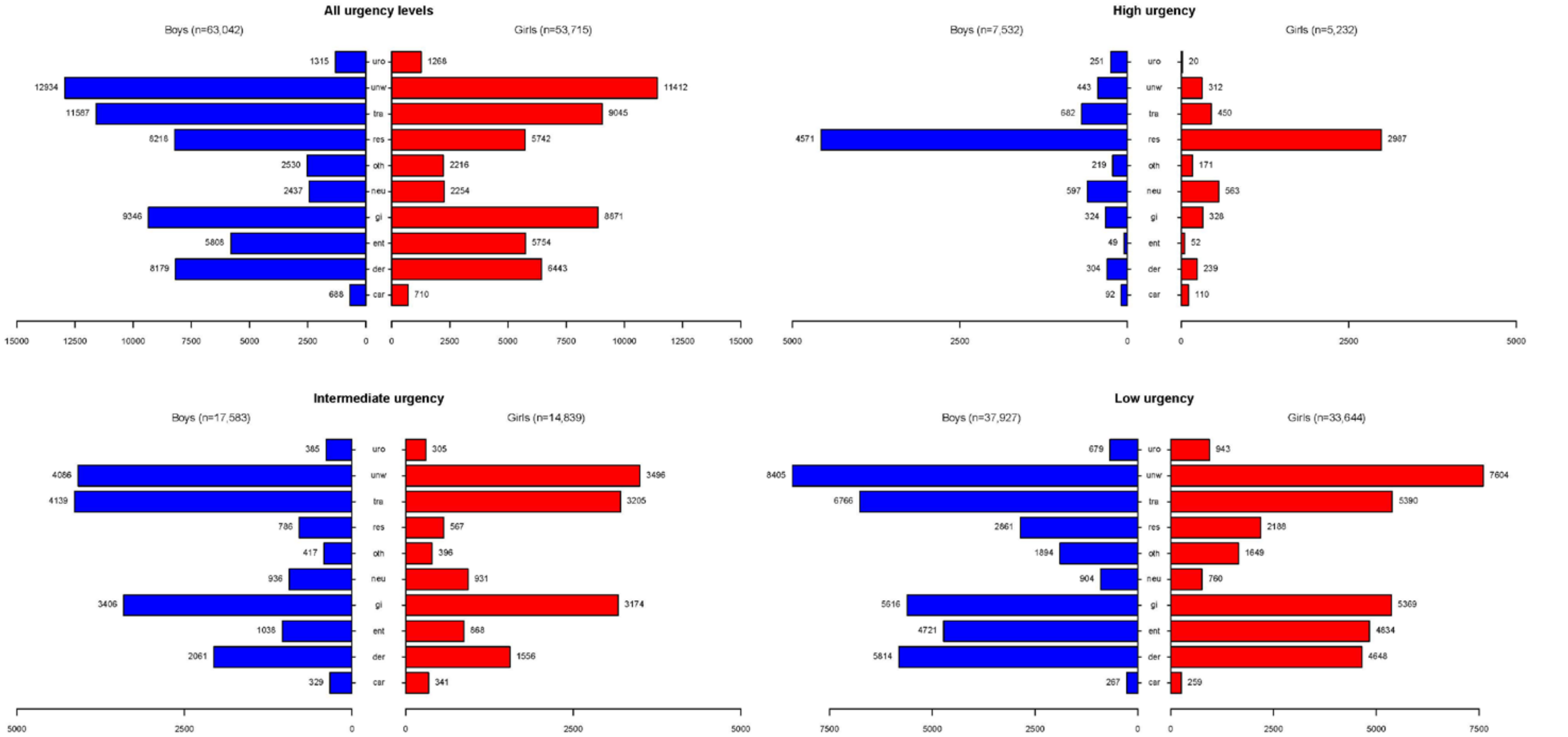

car=cardiac; der=dermatological; ent=ear, nose and throat; gi=gastrointestinal; neu=neurologic or psychiatric; oth=other; res=respiratory; tra=trauma or musculoskeletal; unw=general malaise, uro=uro- or gynaecological

For peer review only - http://bmjopen.bmj.com/site/about/guidelines.xhtml

APPENDIX 5. Associations of sex with ED diagnostics and management in subgroups

Figure 5.1 Associations of sex with ED diagnostics and management in patients with trauma or musculoskeletal problems, adjusted for age and triage urgency (boys as reference group)

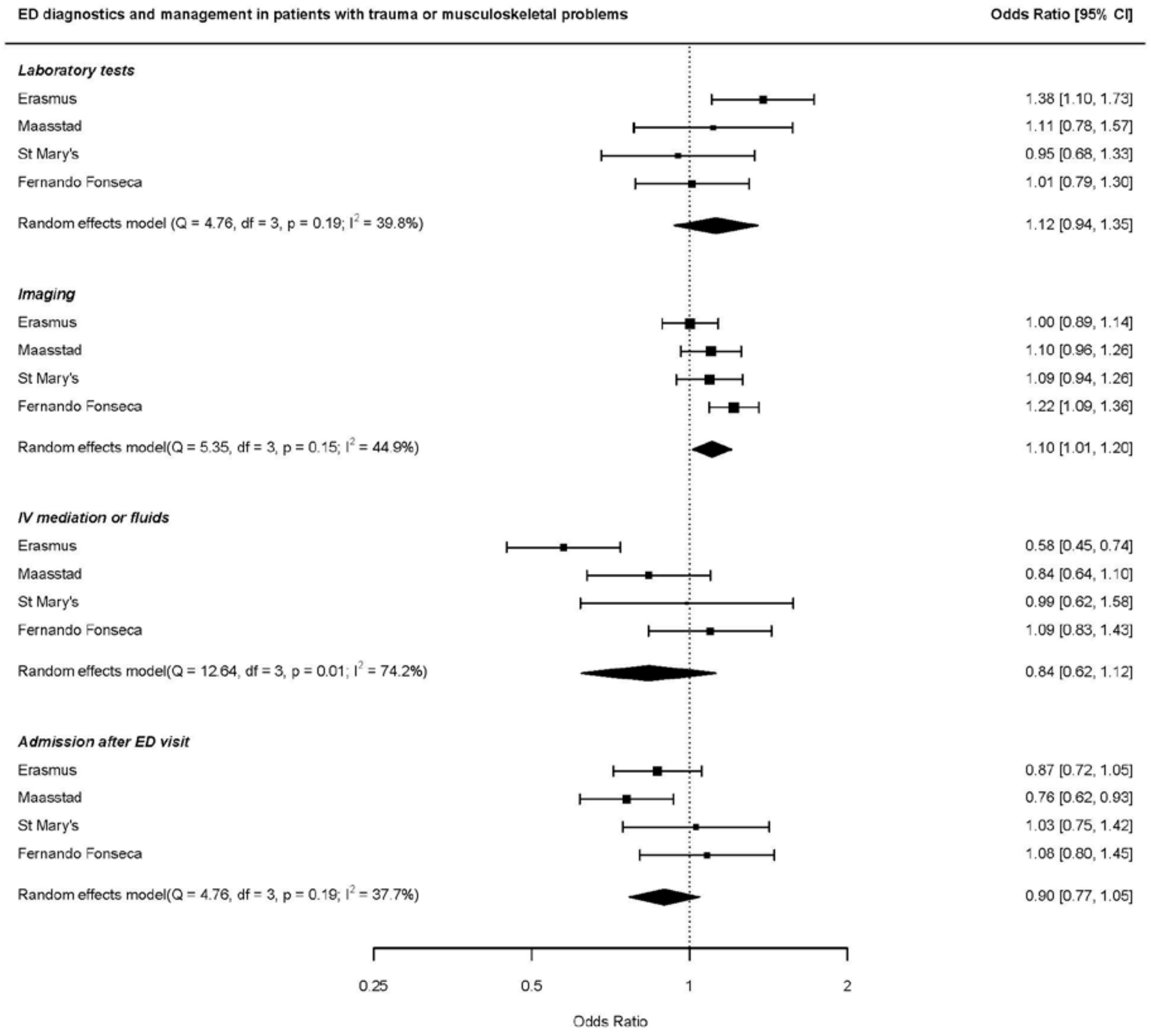

Figure 5.2 Associations of sex with ED diagnostics and management in patients with gastrointestinal problems, adjusted for age and triage urgency (boys as reference group)

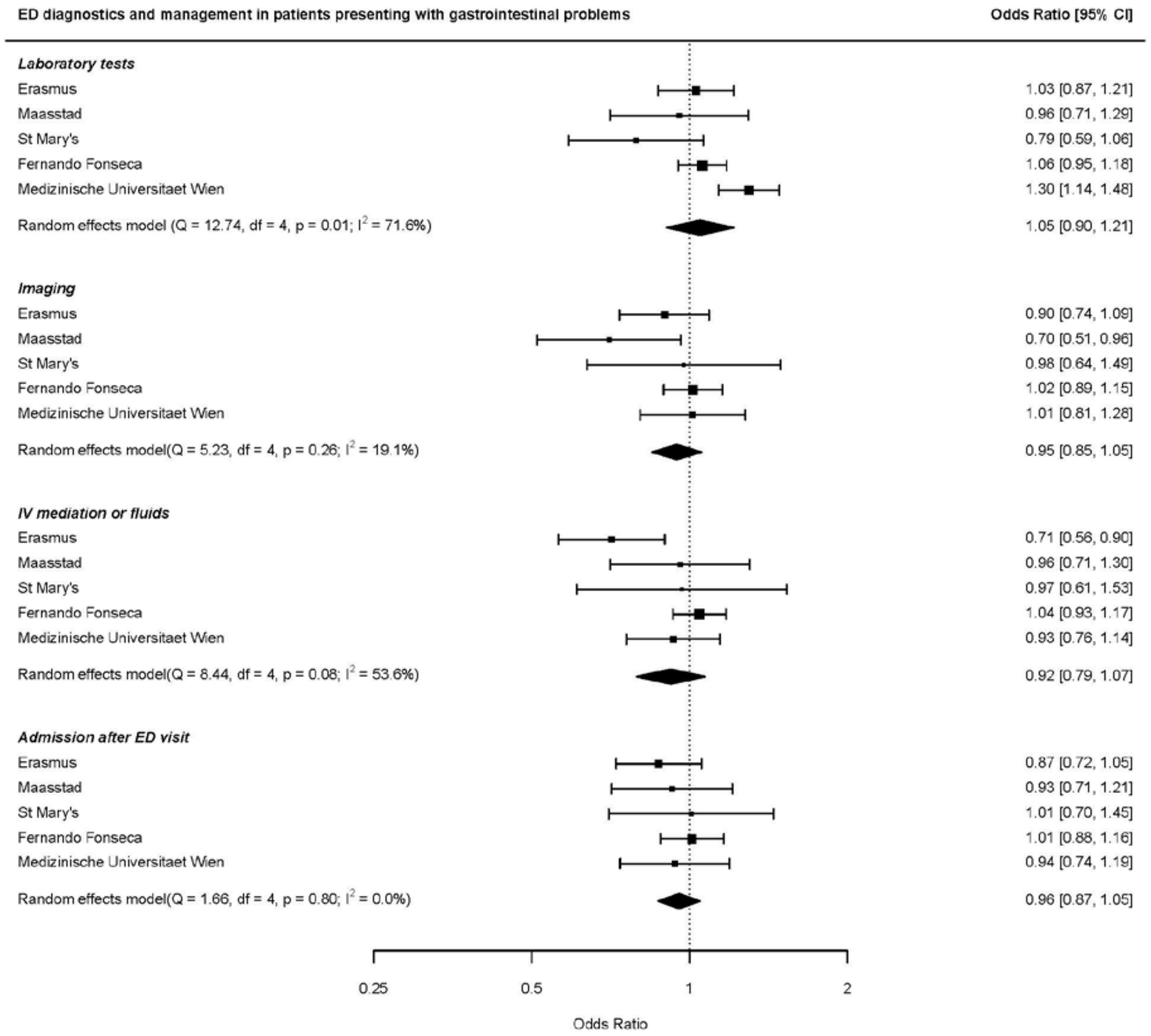

Figure 5.3 Associations of sex with ED diagnostics and management in patients with respiratory problems, adjusted for age and triage urgency (boys as reference group)

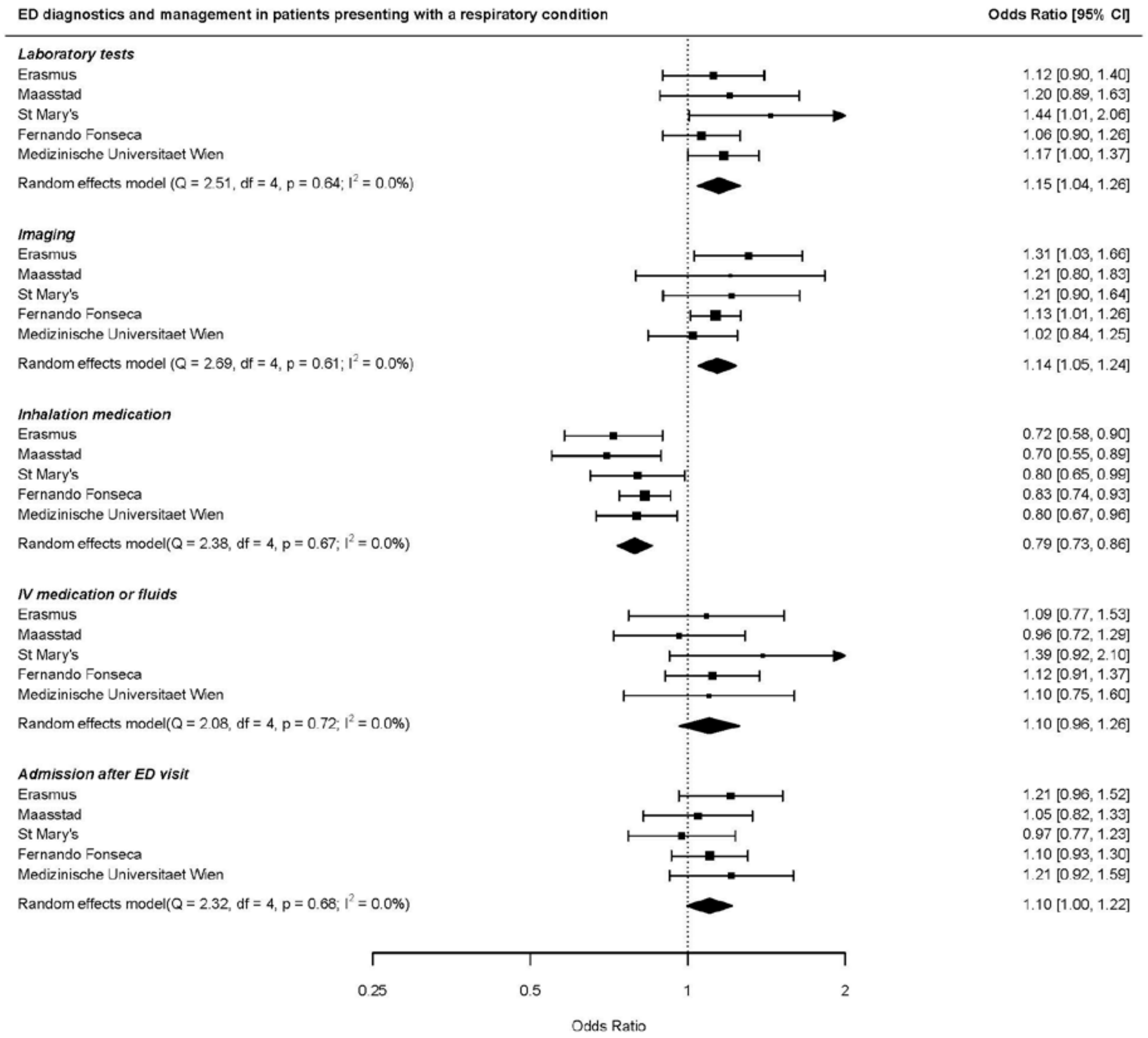

Zachariasse et al. 2020

Figure 5.4 Associations of sex with ED diagnostics and management in patients with fever, adjusted for age and triage urgency (boys as reference group)

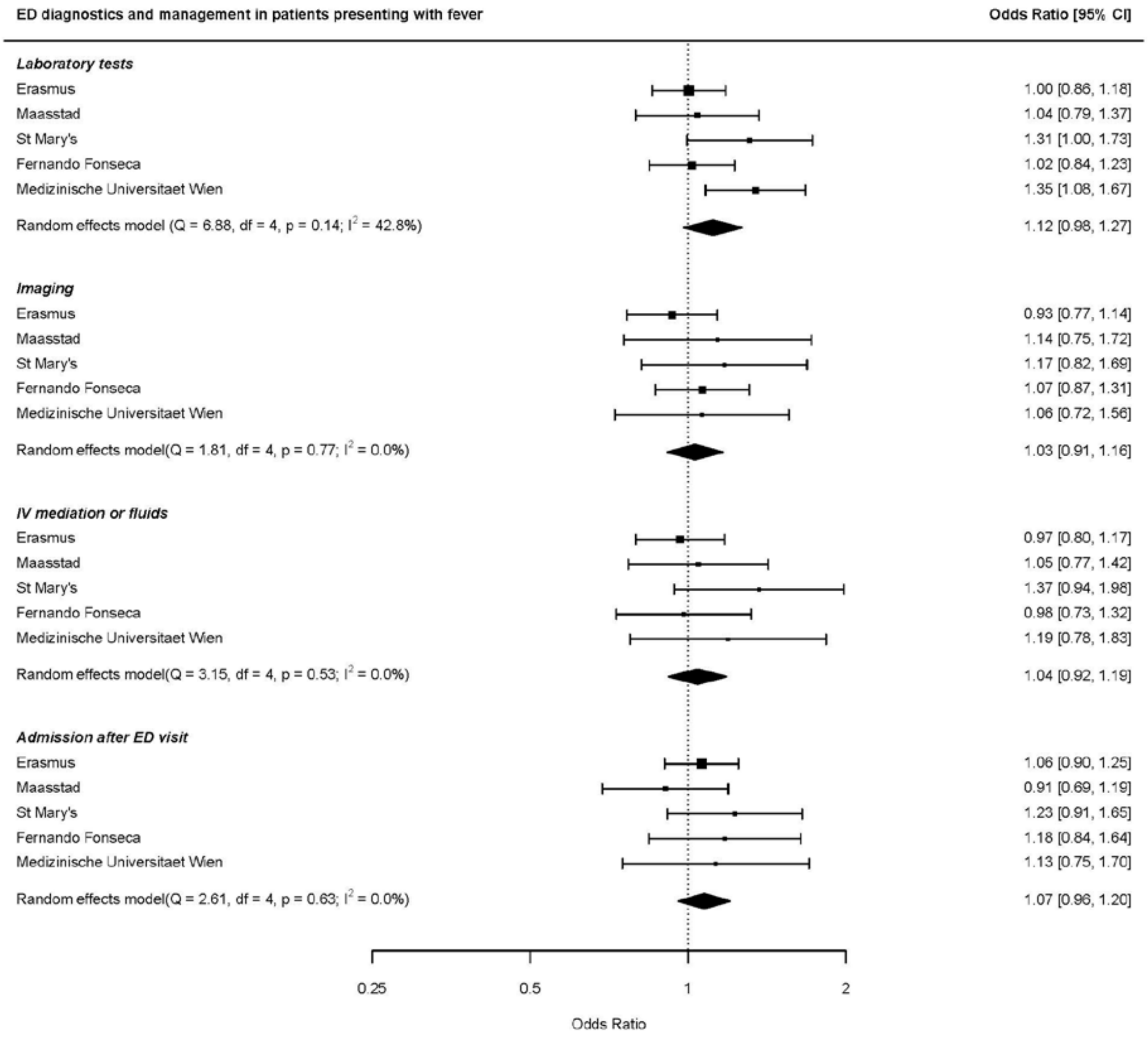

For peer review only - http://bmjopen.bmj.com/site/about/guidelines.xhtml

STROBE Statement—Checklist of items that should be included in reports of ***cohort studies***

"SEX-SPECIFIC DIFFERENCES IN CHILDREN ATTENDING THE EMERGENCY DEPARTMENT: PROSPECTIVE OBSERVATIONAL STUDY", *10-03-2020*

	Item No	Recommendation	
**Title and abstract**	1	(*a*) Indicate the study's design with a commonly used term in the title or the abstract	*The study design, a prospective observational study, is stated in the title and abstract (p1).*
		(*b*) Provide in the abstract an informative and balanced summary of what was done and what was found	*The abstract is written according to the guidelines with all required information (p5-6).*
**Introduction**			
Background/rationale	2	Explain the scientific background and rationale for the investigation being reported	*The background and rationale are described in the first two paragraphs of the introduction section (p8).*
Objectives	3	State specific objectives, including any prespecified hypotheses	*The objective is stated in the last sentence of the introduction (p8). There were no prespecified hypothesis.*
**Methods**			
Study design	4	Present key elements of study design early in the paper	*Key elements of the study design are presented in the first paragraph of the methods (p9), and in detail explained in the consequent paragraphs under subheadings "Study settings and patient population", "Data collection" and "Data analysis".*
Setting	5	Describe the setting, locations, and relevant dates, including periods of recruitment, exposure, follow-up, and data collection	*The setting, location and relevant dates are described in paragraph 'Study settings and patient population', in the methods section (p9). A detailed description of all settings is added in supplemental information 2.*
Participants	6	(*a*) Give the eligibility criteria, and the sources and methods of selection of participants. Describe methods of follow-up	*All children under the age of 16 attending the ED during the study period were eligible, as reported in the paragraph 'Study settings and patient population' in the methods section (p9).*
		(*b*) For matched studies, give matching criteria and number of exposed and unexposed	*Not applicable*
Variables	7	Clearly define all outcomes, exposures, predictors, potential confounders, and effect modifiers. Give diagnostic criteria, if applicable	*The main determinant of the study is sex, defined as male or female. The determinant and all outcomes are described in the 'Determinants'*

For peer review only - http://bmjopen.bmj.com/site/about/guidelines.xhtml

			paragraph in the methods section (p10).
Data sources/ measurement	8*	For each variable of interest, give sources of data and details of methods of assessment (measurement). Describe comparability of assessment methods if there is more than one group	*All variables are routinely recorded in patients' electronic health records, and automatically extracted, as reported in the paragraph 'Data collection' of the methods section (p9).*
Bias	9	Describe any efforts to address potential sources of bias	*The study is based on extraction of routinely collected data (p9). ED staff involved in the data collection were not aware of the research question, thereby minimizing information bias The number of patients with missing data was very small (2%), thereby limiting bias due to missing data (p12).*
Study size	10	Explain how the study size was arrived at	*Because of the observational nature of the study, we did not perform a formal sample size calculation. As a general rule of thumb, a regression model should have at least 10-15 cases with the outcome for each of the predictors included in the model. As can be seen in table 1, all relevant outcomes occurred in more than 1000 patients, enough to study more than 66 determinants.*
Quantitative variables	11	Explain how quantitative variables were handled in the analyses. If applicable, describe which groupings were chosen and why	*The handling of variables is described in the 'Determinants' paragraph of the methods section (p10).*
Statistical methods	12	(*a*) Describe all statistical methods, including those used to control for confounding	*The statistical methods are described in the 'Data analysis' paragraph of the methods section (p10-11).*
		(*b*) Describe any methods used to examine subgroups and interactions	*Based on the reviewer comments, we performed subgroup analyses in the most prevalent clinical presentations (p10, appendix 3).*
		(*c*) Explain how missing data were addressed	*Because the number of patients with missing data was very small (2%) we used a complete case approach, as described in the paragraph 'Study settings and patient population'(p9)'.*
		(*d*) If applicable, explain how loss to follow-up was addressed	*Not applicable*
		(*e*) Describe any sensitivity analyses	*Not applicable*
**Results**			
Participants	13*	(a) Report numbers of individuals at each stage of study—eg numbers potentially	*The patient flow is described in Figure 1 and in the first paragraph of the*

For peer review only

		eligible, examined for eligibility, confirmed eligible, included in the study, completing follow-up, and analysed	*results section (p12). All tables include the numbers of patients that were analysed.*
		(b) Give reasons for non-participation at each stage	*Not applicable*
		(c) Consider use of a flow diagram	-
Descriptive data	14*	(a) Give characteristics of study participants (eg demographic, clinical, social) and information on exposures and potential confounders	*Characteristics of the study population are described in table 1 and supplemental information 4.*
		(b) Indicate number of participants with missing data for each variable of interest	*The number of participants with missing data is provided in the flow diagram (Figure 1) and in the 'Study settings and patient population' paragraph of the methods section (p9). Missing information was limited to only two variables: triage urgency and triage flowchart (presenting problem)*
		(c) Summarise follow-up time (eg, average and total amount)	*Not applicable*
Outcome data	15*	Report numbers of outcome events or summary measures over time	*The number of patients with the outcome are reported in table 1.*
Main results	16	(*a*) Give unadjusted estimates and, if applicable, confounder-adjusted estimates and their precision (eg, 95% confidence interval). Make clear which confounders were adjusted for and why they were included	*Unadjusted estimates, i.e. absolute numbers are presented in table 1. Adjusted estimates and 95% confidence intervals are presented in figures 1 and 2. Analyses were adjusted for age and triage urgency as mentioned in the "Data analysis" paragraph (p10-11).*
		(*b*) Report category boundaries when continuous variables were categorized	*The only continuous variable, age, was maintained continues, as described in the "Determinants" paragraph of the methods section (p10).*
		(*c*) If relevant, consider translating estimates of relative risk into absolute risk for a meaningful time period	*Not applicable*
Other analyses	17	Report other analyses done—eg analyses of subgroups and interactions, and sensitivity analyses	*Pre-specified subgroup analyses were performed and described in the "Data analyses" paragraph of the methods section (p10-11), in the results section (p12-13) and in supplemental information 6.*
**Discussion**			
Key results	18	Summarise key results with reference to study objectives	*Key results are stated in the first paragraph and discussed in the subsequent paragraphs of the discussion section (p14-15).*

For peer review only - http://bmjopen.bmj.com/site/about/guidelines.xhtml

Limitations	19	Discuss limitations of the study, taking into account sources of potential bias or imprecision. Discuss both direction and magnitude of any potential bias	*Limitations of the study are addressed in the a separate "Limitations" paragraph of the discussion, (p15).*
Interpretation	20	Give a cautious overall interpretation of results considering objectives, limitations, multiplicity of analyses, results from similar studies, and other relevant evidence	*The interpretation of the study is discussed in the 2nd to 4th paragraphs of the discussion (p14-15).*
Generalisability	21	Discuss the generalisability (external validity) of the study results	*Generalisability of the study is discussed in the 2nd paragraph of the discussion (p14).*
**Other information**			
Funding	22	Give the source of funding and the role of the funders for the present study and, if applicable, for the original study on which the present article is based	*This research received no funding. This is described on the title page (p3).*

*Give information separately for exposed and unexposed groups.

Note: An Explanation and Elaboration article discusses each checklist item and gives methodological background and published examples of transparent reporting. The STROBE checklist is best used in conjunction with this article (freely available on the Web sites of PLoS Medicine at http://www.plosmedicine.org/, Annals of Internal Medicine at http://www.annals.org/, and Epidemiology at http://www.epidem.com/). Information on the STROBE Initiative is available at http://www.strobe-statement.org.

For peer review only
